# Negative Gauge Factor Piezoresistance in Soft Strain and Pressure Sensors: Mechanisms, Materials, and Emerging Applications

**DOI:** 10.1002/advs.78571

**Published:** 2026-11-10

**Authors:** Su‐Hyeong Lee, Minjae Kim, Soosang Chae

**Affiliations:** ^1^ School of Energy Materials and Chemical Engineering Korea University of Technology and Education Cheonan South Korea

**Keywords:** conducting polymers, low‐power sensing, negative gauge factors, piezoresistive sensors, soft electronics, stretchable strain sensors

## Abstract

Soft strain and pressure sensors underpin wearable electronics, soft robotics, and human‐centered healthcare, yet most piezoresistive devices rely on a positive gauge factor (pGF), in which resistance increases as deformation disrupts conductive pathways and continuous power is needed to register an event. Negative‐gauge‐factor (nGF) materials, whose resistance decreases under deformation, offer a fundamentally different route. Importantly, a negative gauge factor does not by itself imply low power: continuously conducting nGF devices still require an applied bias and constant monitoring, whereas near‐zero‐standby‐power, event‐triggered operation is enabled specifically by switch‐type nGF devices that are insulating at rest and turn on only under strain. This review classifies reported nGF systems into four mechanistic categories: intrinsic nGF from strain‐induced band‐structure modulation (Si, MoS_2_, PtSe_2_/PdSe_2_, SiC); composite nGF that transfers this response into polymer matrices and textiles; polymer‐reorganization nGF in conducting polymers; and architecture‐induced nGF engineered through tunneling barriers, contact closure, and compliant geometries. We discuss how these mechanisms enable low‐power switch‐type sensors, wearable health and motion monitoring, electronic skins, and smart textiles, and outline the challenges to establishing nGF piezoresistance as a design principle for energy‐efficient soft electronics.

## Graphical abstract

Negative‐gauge‐factor materials reverse the usual rule of piezoresistive sensing: their resistance falls, rather than rises, under deformation. This review organizes such behavior into four mechanistic classes spanning intrinsic semiconductors, composites, conducting polymers, and engineered architectures. The unifying theme is that strain activates conduction, opening a path toward switch‐type, event‐triggered sensors that consume almost no standby power.

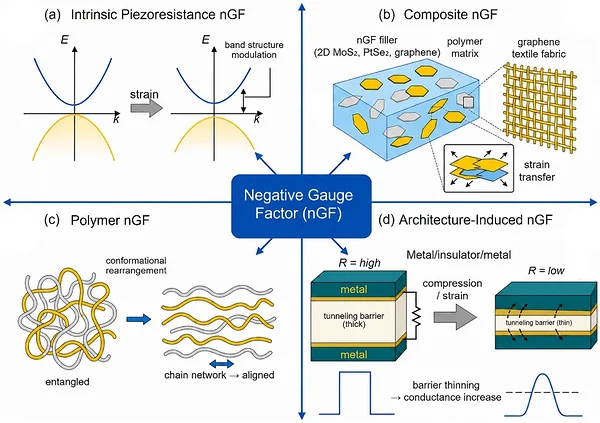

## 1 Introduction

The growing demand for wearable electronics, soft robotics, and human‐centered healthcare technologies has created a strong need for soft strain and pressure sensors that can detect subtle mechanical stimuli with high sensitivity, mechanical compliance, and long‐term reliability [1, 2, 3, 4]. Although conventional strain gauges have long exploited the principle that mechanical deformation induces changes in electrical properties, they are typically limited to small deformation ranges and are therefore insufficient for emerging soft and wearable systems that require stable sensing under large strains [5, 6, 7]. In this context, piezoresistive sensors, which convert strain or pressure into resistance changes, have become one of the most practical soft sensing platforms owing to their simple device architecture, straightforward resistance‐based readout, rapid response, cost‐effective fabrication, low‐power operation, and facile integration into flexible films, stretchable composites, electronic skins, textiles, and biomedical monitoring devices [8, 9].

In piezoresistive sensing, the sensitivity of a strain sensor is commonly evaluated using the gauge factor (GF), which is defined as the relative change in electrical resistance normalized by the applied mechanical strain: GF = (Δ*R*/*R*_0_)/ε, where *R*_0_ is the initial resistance, *R*_ε_ is the resistance under a given strain *ε*, and Δ*R* = *R*_ε_ − *R*_0_. A positive gauge factor (pGF) indicates that the resistance increases upon mechanical deformation, whereas a negative gauge factor (nGF) means that the resistance decreases under applied strain or pressure (Figure 1a,b). Most soft piezoresistive strain sensors developed to date have been designed to exhibit a positive GF, because their sensing mechanism is generally based on the deformation‐induced disruption of conductive pathways [10, 11, 12]. In conductive polymer composites, for example, stretching increases the distance between conductive fillers, weakens percolated networks, or suppresses tunneling transport, resulting in an increase in resistance [13, 14]. Similarly, in structurally engineered metallic conductors, mechanical deformation often leads to crack opening, junction separation, or geometrical elongation of conductive traces, which also produces a positive resistance response [6, 15]. As a result, positive piezoresistive behavior has been the dominant operating principle for soft and stretchable strain sensors, particularly when high sensitivity is required.

**FIGURE 1:**
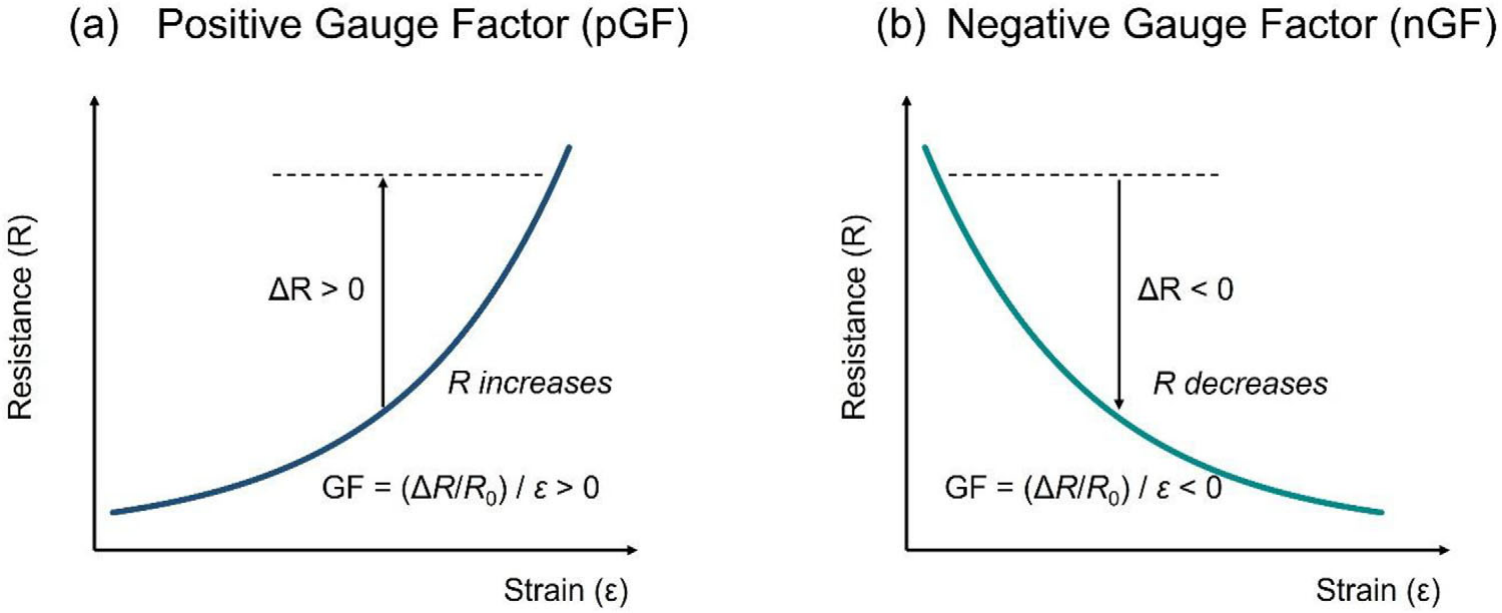
Positive and negative gauge‐factor responses in piezoresistive strain sensing. (a) In a conventional positive‐gauge‐factor (pGF) material, tensile strain increases resistance, giving ΔR > 0 and GF = (ΔR/R_0_)/ε > 0. (b) In a negative‐gauge‐factor material, tensile strain decreases resistance, giving ΔR < 0 and GF < 0. This sign reversal provides the operational basis for low‐power and switch‐type strain sensors.

To avoid ambiguity across the systems surveyed here — which include tensile, compressive, and pressure loading — we adopt a consistent sign convention and distinguish four related quantities. (i) Tensile nGF, the primary focus of this review, denotes a resistance that decreases under tensile strain, that is, GF = (ΔR/R_0_)/ε < 0 with ε > 0 defined as tensile. (ii) Compressive piezoresistance describes the response under compressive loading; because the sign of the gauge factor depends on whether compressive strain is taken as negative or positive ε, we state the loading sign explicitly wherever compression is discussed, and we note that a resistance decrease under compression corresponds to a positive conductance–strain response rather than to tensile nGF. (iii) Pressure sensitivity, used for pressure sensors, is defined as S = (ΔR/R_0_)/ΔP (or its conductance analogue) and carries units of inverse pressure. (iv) Conductance‐based sensitivity is used for switch‐type devices whose response is more naturally expressed as a conductance or current on/off ratio than as a differential gauge factor. Accordingly, compression‐mode systems such as conductive foams — in which conductance rises under compressive load — are identified throughout as the compressive analogue of tensile nGF and are not reported as tensile‐nGF results.

Because most nGF systems display a strongly non‐monotonic resistance–strain relationship, the numerical gauge factor depends on how it is extracted. We distinguish four definitions: the differential gauge factor, GF_diff_ = d(ΔR/R_0_)/dε, the local slope at a specified strain; the secant gauge factor, GF_sec_ = (ΔR/R_0_)/ε, evaluated between the relaxed state and a stated strain; the average gauge factor over a specified interval; and the peak gauge factor, the maximum |GF| reported. Values obtained under different definitions or over different strain windows are not directly comparable, and we therefore state the definition and the strain interval wherever a gauge factor is quoted (Table 2).

The exclusive reliance on positive resistance changes is not always ideal for emerging soft sensor applications, especially when power consumption, standby operation, and event‐triggered sensing become critical design requirements [16, 17]. In a conventional positive‐GF sensor, the device must typically remain electrically active or continuously monitored to detect a deformation event, which can lead to non‐negligible power consumption during long‐term operation. In contrast, a negative‐GF material or device offers a fundamentally different sensing strategy — but it is important to distinguish two operating regimes. A continuous nGF device, whose resistance decreases smoothly with strain from an already‐conductive relaxed state, still requires an applied bias and continuous monitoring and therefore does not by itself reduce standby power; its advantage lies instead in high‐sensitivity, sign‐selectable proportional sensing. Near‐zero‐standby‐power operation is enabled specifically by switch‐type nGF devices that possess a high‐resistance or nearly insulating relaxed state and undergo a switch‐like insulator‐to‐conductor transition under deformation: almost no current flows before the mechanical event, while the resistance drop produces a clear electrical signal only when needed [16, 17]. This concept is especially attractive for energy‐efficient soft sensors used in long‐duration monitoring systems, such as wearable health‐monitoring devices, soft robotic skins, implantable biomedical devices, and safety‐critical deformation or crack‐detection systems, where the target event may occur rarely but must be detected immediately.

Accordingly, negative piezoresistive effects have recently attracted increasing attention as an unconventional but promising route toward energy‐efficient soft sensing [16, 17]. Unlike conventional positive‐GF systems, negative‐GF materials require deformation to enhance, rather than suppress, electrical conduction. This behavior can arise from several distinct mechanisms, including strain‐induced modulation of electronic band structure in intrinsic semiconductors [18], strain transfer from a soft matrix to embedded two‐dimensional nanosheets that retain their intrinsic negative piezoresistance [19], conformational alignment of conducting polymer chains under stretching [20], formation of new conductive pathways through structural compression [21], and mechanically induced insulator‐to‐conductor transitions in tunneling‐barrier‐integrated architectures [22]. Although negative piezoresistance is less common than positive piezoresistance, recent studies have reported negative gauge factors in a wide range of material systems, including single‐crystal semiconductors [23], semiconductor nanowires [24], atomically thin transition metal dichalcogenides [19], two‐dimensional nanosheet/polymer composites [25], conducting polymer films and textiles [25], and structurally engineered soft devices [17]. These emerging examples suggest that negative piezoresistance is not merely an anomalous response, but a useful design principle for next‐generation soft sensors that require high sensitivity, mechanical compliance, and reduced energy consumption [16]. Therefore, understanding and engineering negative piezoresistive effects may open a new direction in soft sensor design, shifting the field from continuously powered resistance‐increase sensing toward mechanically activated, energy‐efficient resistance‐decrease sensing.

Despite these developments, negative piezoresistive effects have not yet been systematically reviewed from the perspective of soft strain and pressure sensors, particularly with respect to their potential for energy‐efficient sensing. Previous reviews on piezoresistive sensors have largely focused on positive‐GF materials, high‐sensitivity conductive composites, crack‐based sensors, or stretchable electrode designs, while negative‐GF behavior has usually been treated as a material‐specific anomaly or a secondary observation [9, 10, 11]. Therefore, this review aims to provide a comprehensive overview of negative piezoresistive effects and their relevance to next‐generation soft sensors. In this review, negative‐GF systems are classified into four major categories (Figure 2a–d) according to the origin of the resistance‐decreasing response under mechanical deformation: 1) Intrinsic piezoresistance nGF, where the negative response arises from bulk material properties such as strain‐induced modulation of band structure, carrier density, or carrier mobility, as observed in n‐type silicon, atomically thin MoS_2_, PtSe_2_ and PdSe_2_ films, and silicon carbide nanowires (Figure 2a); 2) Composite nGF, where the intrinsic negative piezoresistance of two‐dimensional semiconductors or other functional fillers is transferred into a soft polymer matrix or textile substrate through interfacial strain transfer, producing a tunable negative response that simultaneously retains mechanical compliance and processability, as reported in MoS_2_‐ and WS_2_‐polymer nanocomposites, solution‐processed PtSe_2_ nanosheet networks, graphene‐coated textile fabrics, and reduced graphene oxide‐functionalized smart fabrics (Figure 2b); 3) Polymer chain reorganization nGF, where deformation‐induced morphological or conformational rearrangement of conducting polymer chains enhances charge transport, as found in PEDOT:PSS, polypyrrole‐coated textiles, and other conjugated polymer systems (Figure 2c); and 4) Architecture‐induced nGF, where the negative response is not an intrinsic material property but is generated by structural design that mechanically modulates interlayer conduction or tunneling, such as Au/PDMS/Au sandwich configurations, tunneling‐barrier‐integrated gold nanofilms, and twisted conductive fibers with precision‐engineered cracking (Figure 2d). Based on this classification, we discuss the material origins, device mechanisms, and sensing implications of nGF behavior, with particular emphasis on its potential to realize low‐standby‐power, event‐triggered, and energy‐efficient soft strain and pressure sensors. This perspective is expected to establish negative piezoresistance as a distinct and useful design framework for future soft mechanical sensing technologies.

**FIGURE 2:**
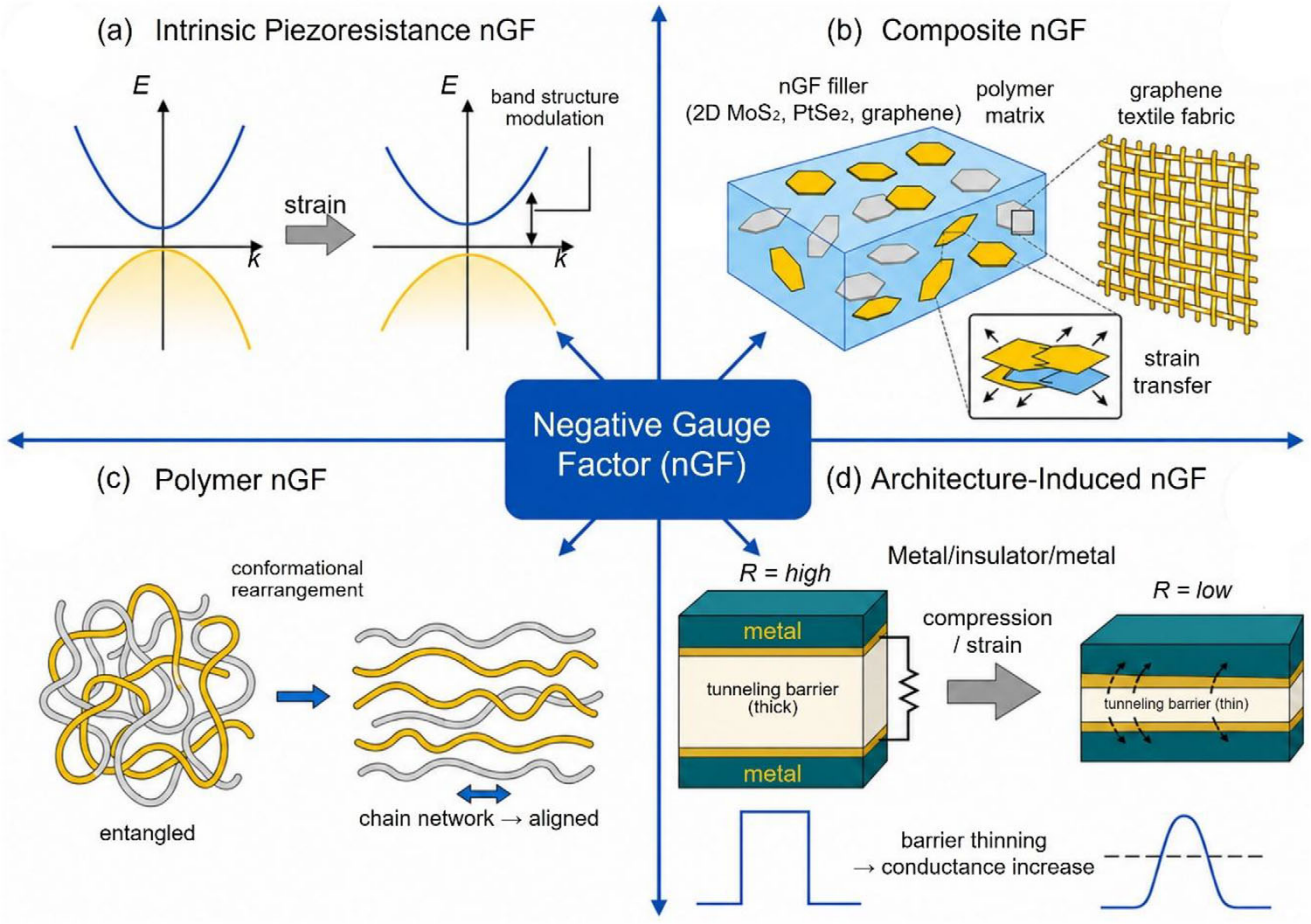
Classification of negative‐gauge‐factor mechanisms. (a) Intrinsic‐piezoresistance nGF originates from strain‐induced modulation of the electronic band structure and carrier transport in the conductor itself. (b) Composite nGF arises when strain transfer to nGF fillers — such as 2D nanosheets or graphene‐based networks — enhances charge transport despite macroscopic elongation. (c) Polymer nGF is driven by strain‐induced conformational or chain‐network reorganization in conducting polymers. (d) Architecture‐induced nGF is generated by engineered geometries — such as metal/insulator/metal tunneling barriers — that convert external strain into barrier thinning, local compression, or contact closure.

To assign a system to a mechanistic class we apply the following criteria in order, using the dominant resistance‐change mechanism: (1) if the negative response arises from the strain response of a single‐phase conductor's own electronic band structure, the system is intrinsic; (2) otherwise, if it arises from strain transfer to a dispersed conductive filler network in a distinct matrix, it is composite; (3) otherwise, if it arises from strain‐induced morphological reorganization of a single‐component conducting polymer, it is polymer‐reorganization; (4) otherwise, if the sign is set by an engineered geometry that re‐couples macroscopic tension into local compression, gap‐thinning, or contact closure, it is architecture‐induced. Boundary cases that satisfy more than one criterion (coated textiles, percolating nanosheet networks, hybrids) are classified by the dominant mechanism and flagged as such in the text.

## 2 Intrinsic Electronic‐Structure‐Driven Negative Gauge Factor Materials

### 2.1 Classical Foundations: Bulk Silicon and Nickel

Intrinsic electronic‐structure‐driven negative gauge factor materials originate from the classical understanding that mechanical stress can modify not only the geometry of a conductor but also its intrinsic electrical resistivity [26, 27, 28, 29, 30, 31]. Early studies on strained metals and single crystals established that resistance changes under mechanical deformation can include a conductivity contribution, not merely the dimensional change expected from elongation and Poisson contraction [28, 31, 32, 33, 34, 35, 36]. This distinction is essential for understanding negative gauge factor behavior, because a decrease in resistance under strain requires the intrinsic conductivity increase to overcome the ordinary positive geometrical contribution [26, 29, 30].

The development of piezoresistance theory progressed from early measurements in polycrystalline metals to direction‐dependent studies in single crystals [27, 28, 32, 33, 34, 35]. Rolnick quantified the tension coefficient of resistance in multiple metals, while Allen reported orientation‐dependent resistance changes in single crystals such as bismuth, antimony, zinc, cadmium, and tin [32, 33, 34, 35, 36]. Bridgman subsequently formulated the effect of homogeneous mechanical stress on the electrical resistance of crystals, providing a tensor‐based framework for later semiconductor piezoresistance [28]. Cookson further clarified the concept of the piezoresistive effect as a stress‐induced conductivity or resistivity change, distinct from the total resistance change that also includes geometrical deformation [29].

An early and widely cited study of semiconductor piezoresistance was Smith's 1954 study on silicon and germanium [30]. Smith experimentally determined the complete piezoresistance tensor of cubic Si and Ge and showed that uniaxial tensile stress produces pronounced resistivity changes in both n‐type and p‐type materials [30]. Importantly, Smith emphasized that the resistance change caused by stress‐induced dimensional deformation is small and can be corrected for, allowing the remaining response to be expressed as a true change in resistivity [30]. This conclusion directly supports the concept of intrinsic nGF: when mechanical deformation enhances carrier transport sufficiently, resistance can decrease even under tensile loading [26, 30, 37, 38].

The physical origin of this large semiconductor piezoresistance lies in strain‐induced modification of carrier transport [26, 30, 37, 38]. In semiconductors, stress can modify carrier mobility, carrier density, effective mass, and band structure, producing resistivity changes far larger than those expected from geometry alone [26, 30, 38, 39]. Barlian et al. describe the gauge factor as the fractional resistance change normalized by strain, GF = (ΔR/R)/ε, and separate the total resistance response into a geometrical term and a material resistivity term (Figure 3a–c) [26]. For most metals, the geometrical term gives a GF of approximately 1.4–2.0 and the resistivity contribution is relatively small, whereas in selected directions of silicon and germanium the resistivity contribution can be 50–100 times larger than the geometrical term [26].

**FIGURE 3:**
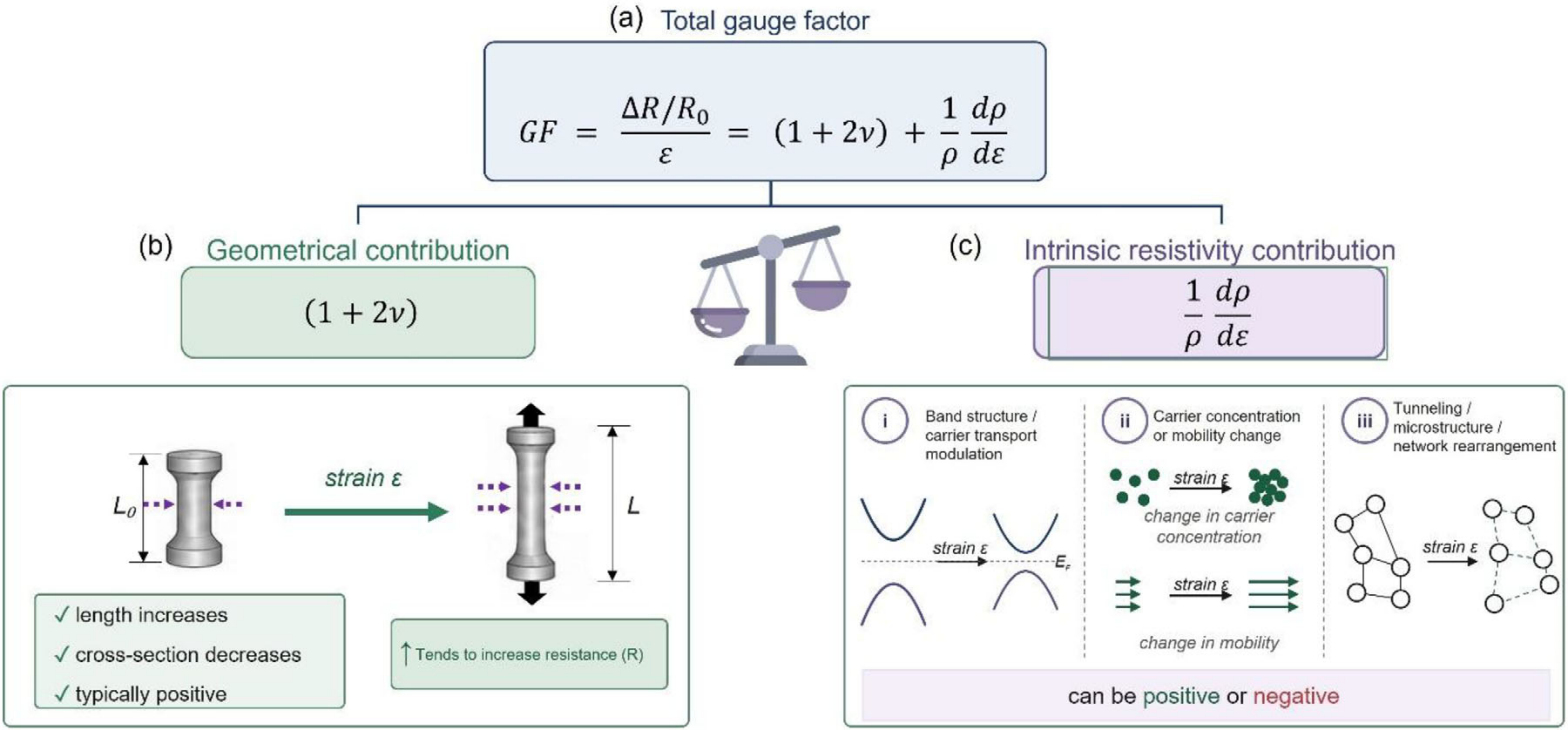
Decomposition of the gauge factor into geometrical and material contributions. (a) The total gauge factor can be separated into a geometrical contribution and an intrinsic resistivity contribution. (b) The geometrical term is usually positive because tensile strain increases length and decreases cross‐sectional area, both of which tend to increase resistance. (c) The intrinsic resistivity term may be positive or negative depending on strain‐induced modulation of band structure, carrier density, mobility, tunneling pathways, microstructure, or conductive‐network topology. Negative‐gauge‐factor behavior appears when the negative intrinsic or structural contribution exceeds the positive geometrical baseline.

In n‐type silicon, the classical explanation for negative piezoresistance is the many‐valley model (Figure 4a,b) [26, 38, 40]. Mechanical strain breaks the symmetry of the conduction‐band valleys and redistributes electrons among valleys with different effective masses and mobilities [26, 38]. Under uniaxial tension, electrons can be transferred into valleys that provide higher mobility along the strain direction, thereby increasing conductivity and decreasing resistance in that direction [26, 38]. This valley‐repopulation mechanism explains why n‐type silicon can exhibit a negative gauge factor, often cited to be approximately −135 depending on crystallographic orientation, doping level, and measurement geometry [24, 25, 30, 41].

**FIGURE 4:**
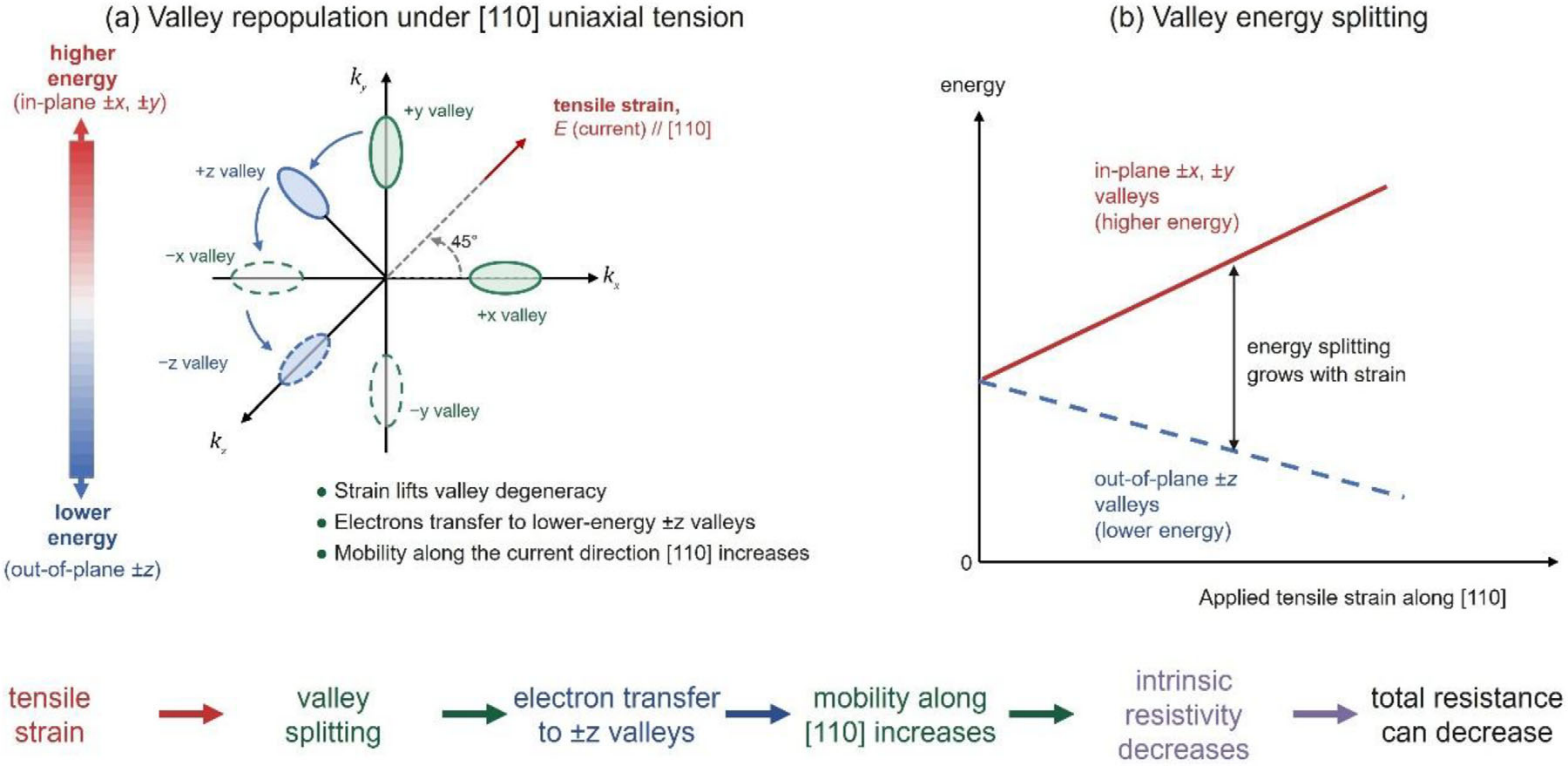
Many‐valley mechanism for negative gauge factor in n‐type silicon. (a) Under uniaxial tensile strain along the [110] direction, the degeneracy of the six conduction‐band valleys in n‐type Si is lifted. The in‐plane (±x, ±y) valleys shift to higher energy, while the out‐of‐plane (±z) valleys become energetically favored. Electron repopulation into the lower‐energy (±z) valleys increases mobility along the [110] current direction and decreases the intrinsic resistivity. (b) The corresponding valley energy splitting increases with applied strain, producing the sequence valley splitting → electron redistribution → enhanced mobility → reduced intrinsic resistivity → possible total resistance decrease. Reproduced/adapted with permission [30]. Copyright 1954, APS.

A key lesson from the classical Si and Ge studies is that the sign and magnitude of piezoresistance are not universal material constants [26, 30, 39, 42]. Instead, they depend on crystal orientation, carrier type, impurity concentration, temperature, and the relative directions of stress, current, and voltage [23, 26, 30, 39]. Smith's tensor formulation revealed exceptionally large shear coefficients in Si and Ge, and subsequent work translated these tensor relationships into practical silicon strain‐gauge and microelectromechanical systems (MEMS) design rules [23, 30, 39, 42, 43]. This body of work established silicon piezoresistance as one of the most important sensing principles in pressure sensors, accelerometers, cantilever force sensors, and integrated MEMS devices [23, 26, 42, 43, 44, 45].

In addition to semiconductors, nickel is often cited as a rare metallic material exhibiting a negative gauge factor [24, 25, 41]. While most metals show small positive gauge factors dominated by geometrical elongation, Ni has been reported to exhibit a modest negative GF of approximately −12, attributed to strain‐induced enhancement of electrical conductivity [24, 41]. This value is much smaller in magnitude than that of n‐type silicon, but it is conceptually important because it demonstrates that negative piezoresistance is not restricted to semiconductors [24, 25, 41]. Nevertheless, the role of Ni in this subsection should remain mainly historical and comparative: unlike silicon, where negative GF is strongly connected to band‐structure and valley‐transport physics, the Ni example primarily illustrates that a negative resistance response can also occur in metals when the intrinsic conductivity term dominates over the positive geometrical term [24, 41].

However, despite their importance, these classical materials are not ideal for emerging soft strain and pressure sensors. Bulk silicon is rigid and brittle, while metallic Ni does not provide the large deformability or soft mechanical compliance required for wearable electronics, electronic skins, and soft robotic systems. This limitation motivated the exploration of low‐dimensional and flexible materials, such as silicon nanowires, SiC nanowires, and atomically thin semiconductors, where mechanical deformation can be more effectively accommodated and electronic structures can be more strongly modulated [11, 18, 31, 46, 47, 48, 49, 50, 51, 52]. Thus, the classical piezoresistance of bulk Si and Ni provides the physical foundation for understanding modern intrinsic nGF systems, while also clarifying why new material platforms are needed for energy‐efficient soft strain and pressure sensors.

### 2.2 Low‐Dimensional Amplification: Silicon and Silicon Carbide Nanowires

While bulk silicon establishes the physical foundation for intrinsic negative piezoresistance, its rigidity, brittleness, and limited elastic deformability fundamentally restrict its applicability to soft strain and pressure sensors. To overcome these constraints while preserving—and even amplifying—the band‐structure‐driven negative piezoresistive response, recent research has turned to low‐dimensional silicon and silicon carbide nanostructures, where reduced dimensionality, surface effects, and elastically achievable strain levels combine to produce piezoresistive coefficients that are orders of magnitude larger than those of the corresponding bulk crystals [18, 53, 54, 55, 56]. In these quasi‐one‐dimensional systems, the carriers are confined and partially decoupled from the bulk band structure, while the mechanical compliance of nanowires permits elastic strain levels approaching three to five percent—far beyond the fracture limit of bulk silicon—thereby allowing the intrinsic resistivity term in the gauge factor to dominate over the geometrical contribution in regimes that bulk crystals can never access [12, 57].

An early and influential demonstration of giant piezoresistance in silicon nanowires was reported by He and Yang in 2006 (Figure 5a–c) [18]. Using vapor–liquid–solid (VLS) growth of p‐type ⟨111⟩‐oriented Si nanowires and four‐point electrical measurement under controlled uniaxial strain, they reported a longitudinal piezoresistance coefficient π_l_ reaching as high as −3550 × 10^−^^1^ ^1^ Pa^−^^1^, in comparison with the bulk value of approximately −94 × 10^−^^1^ ^1^ Pa^−^^1^—an amplification of nearly thirty‐seven fold [18, 30, 39]. Importantly, the magnitude of the piezoresistance coefficient was found to be strongly diameter‐dependent, increasing systematically as the nanowire diameter decreased, indicating that surface‐related electronic effects play a central role in the amplification [18]. Although the absolute gauge factor in this study was extracted in the linear, low‐strain regime where the response is well described by classical many‐valley physics modified by diameter‐dependent surface effects, this result fundamentally established that piezoresistance in nanostructured silicon is not merely a scaled‐down version of the bulk effect, but rather a distinct regime in which surface trapping, dimensional confinement, and modified carrier transport conspire to produce far stronger resistivity changes per unit strain [18, 56].

**FIGURE 5:**
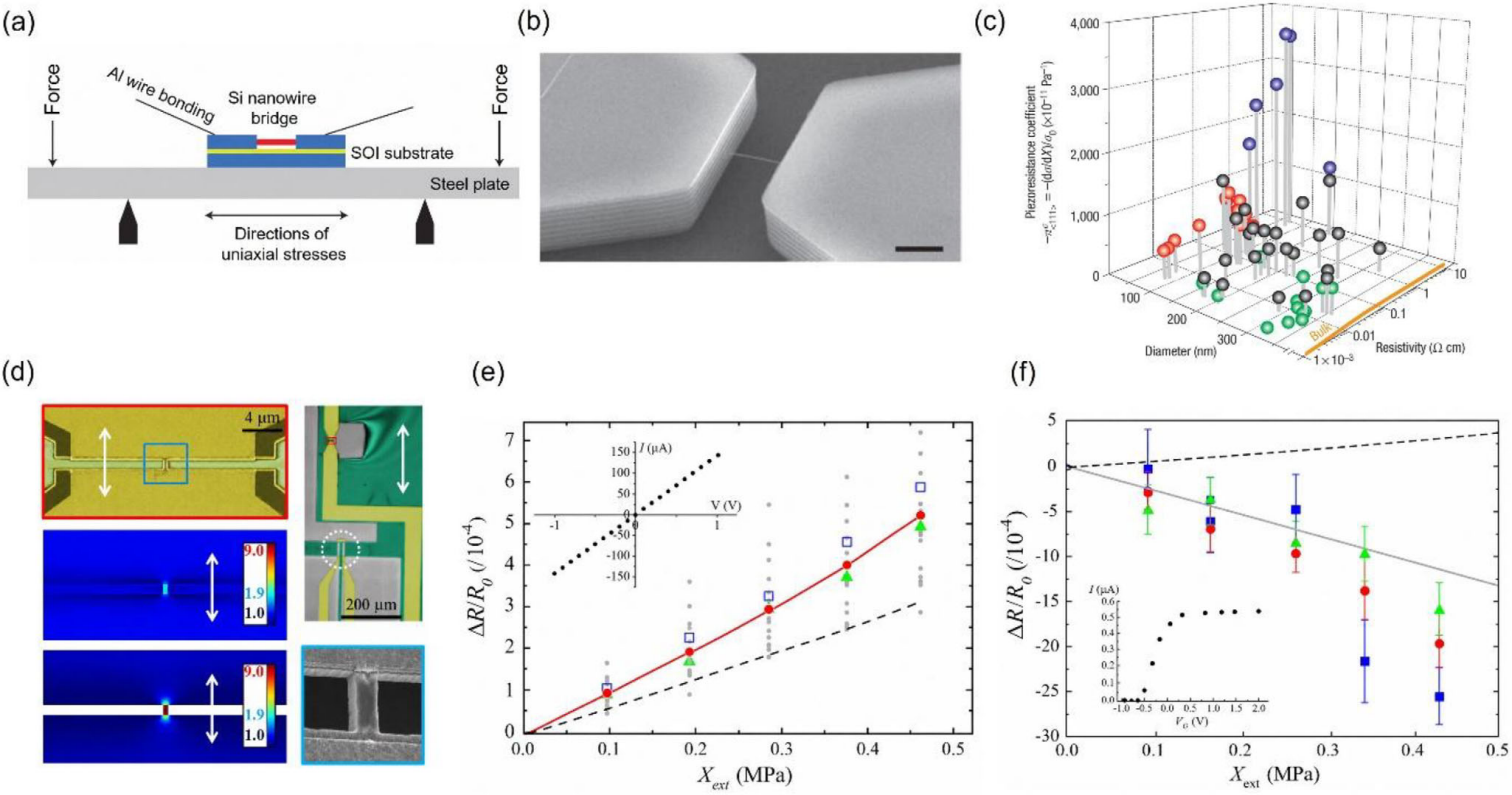
Experimental and analytical routes for low‐dimensional silicon‐nanowire piezoresistance. (a) Schematic of a uniaxial bending configuration used to apply tensile or compressive stress to a Si nanowire bridge on an silicon‐on‐insulator (SOI) substrate. (b) Representative micrograph of a suspended Si nanowire bridge connected by metallic contacts (scale bar is 2 µm). (c) First‐order longitudinal piezoresistance coefficients of p‐type Si nanowires plotted as a function of nanowire diameter and resistivity. Different colors indicate distinct nonlinear piezoresistive response types, and vertical drop lines serve as visual guides. Reproduced/adapted with permission [18]. Copyright 2006, Springer Nature. (d) Sample images and finite‐element stress‐concentration factors for a ⟨110⟩ Si nanowire under uniaxial tension (applied along the white arrows): β ≈ 1.9 before release and β ≈ 9 after release. (e) Positive piezoresistance of non‐released nanowires (gray points); the average response (red) has a slope ≈ 1.7× that of the co‐located strain gauge (dashed line), matching β and identifying stress concentration as the geometric origin of the apparent giant piezoresistance (PZR) (inset: linear I–V). (f) Anomalous negative piezoresistance of naturally re‐oxidized, released nanowires, with magnitude far exceeding bulk p‐Si (dashed line); gating (inset) shows the wires have become n‐type — consistent with the negative sign — and scaling the bulk n‐type response by β ≈ 9 (gray line) reproduces the measured data. Reproduced/adapted with permission [56]. Copyright 2016 AIP Publishing.

A second and equally important advance came from work that extended Si nanowires into the ultra‐high strain regime, where the piezoresistive response becomes strongly nonlinear and, remarkably, undergoes a sign reversal. Lugstein and co‐workers demonstrated that p‐type Si nanowires grown by VLS and integrated into a microelectromechanical loading module exhibit a conventional positive piezoresistance for elongations below approximately 0.8%—exactly as classical theory predicts—but transition to an anomalous negative piezoresistance at higher strain levels [55]. At a maximum tensile strain of 3.5%, a value unattainable in bulk silicon, the nanowire resistance decreased by a factor of ten, corresponding to an effective gauge factor of order 10^2^ and a clear nGF behavior of magnitude far exceeding the conventional pGF response in the same material. No fatigue or fracture was observed even after several hundred loading cycles, indicating that single‐crystal nanowire geometry allows sustained operation in a strain regime where the intrinsic resistivity term dominates over the geometrical contribution. This pGF‐to‐nGF transition under increasing tensile strain has subsequently been interpreted in terms of strain‐induced redistribution of carriers between valleys, modification of effective mass and mobility, and, in some interpretations, charge transfer between core and surface states of the nanowire [55, 56, 58].

Closely related work by McClarty and co‐workers further clarified the physical origin of the giant and anomalous piezoresistive effects in Si nanowires by performing systematic measurements that allowed separation of the geometric and chemical (electronic‐structure) contributions to the gauge factor (Figure 5d–f) [56]. By comparing nanowires with different surface terminations, doping levels, and diameters, they showed that the colossal piezoresistance reported in the smallest Si nanowires is dominated by a surface‐mediated electronic term rather than by purely bulk band‐structure modulation, with surface charge trapping and depletion‐layer modulation under strain accounting for the majority of the observed resistivity change [56]. This finding clarifies why the magnitude of the negative gauge factor in Si nanowires scales with diameter, and why ultrahigh strain can drive the sign reversal observed in earlier studies once the surface‐related resistivity term outweighs the geometric elongation contribution [55, 56]. Together, these studies establish a coherent picture in which the negative piezoresistance of Si nanowires can be tuned through three coupled handles: diameter, which controls the surface‐to‐volume ratio; strain magnitude, which determines whether the geometric or resistivity term dominates; and surface chemistry, which sets the density of strain‐sensitive trap states [18, 55, 56, 58].

Silicon carbide nanowires represent an even more striking platform for negative gauge factor behavior, with reported piezoresistive responses that exceed those of Si nanowires by an order of magnitude or more [53, 54, 59]. Li and co‐workers reported a giant negative piezoresistance effect in 3C‐SiC nanowires synthesized by polysilazane pyrolysis and doped with boron during growth, observing a transverse piezoresistance coefficient as large as π_⊥_ ≈ −103 × 10^−^^1^ ^1^ Pa^−^^1^ at moderate loading and an effective gauge factor of approximately −620 under increased tip force (Figure 6a–d) [54]. The measurements, performed by conductive atomic force microscopy under controlled tip loading, revealed a strongly nonlinear stress dependence of the piezoresistance coefficient, consistent with a regime in which strain‐induced modifications of the conduction band structure of cubic SiC produce dramatic resistivity decreases under increasing tensile load. This result already exceeded the maximum reported value for the best Si nanowires, and demonstrated that the wider band gap and more anisotropic effective mass of SiC offer a fundamentally larger headroom for strain‐induced band restructuring.

**FIGURE 6:**
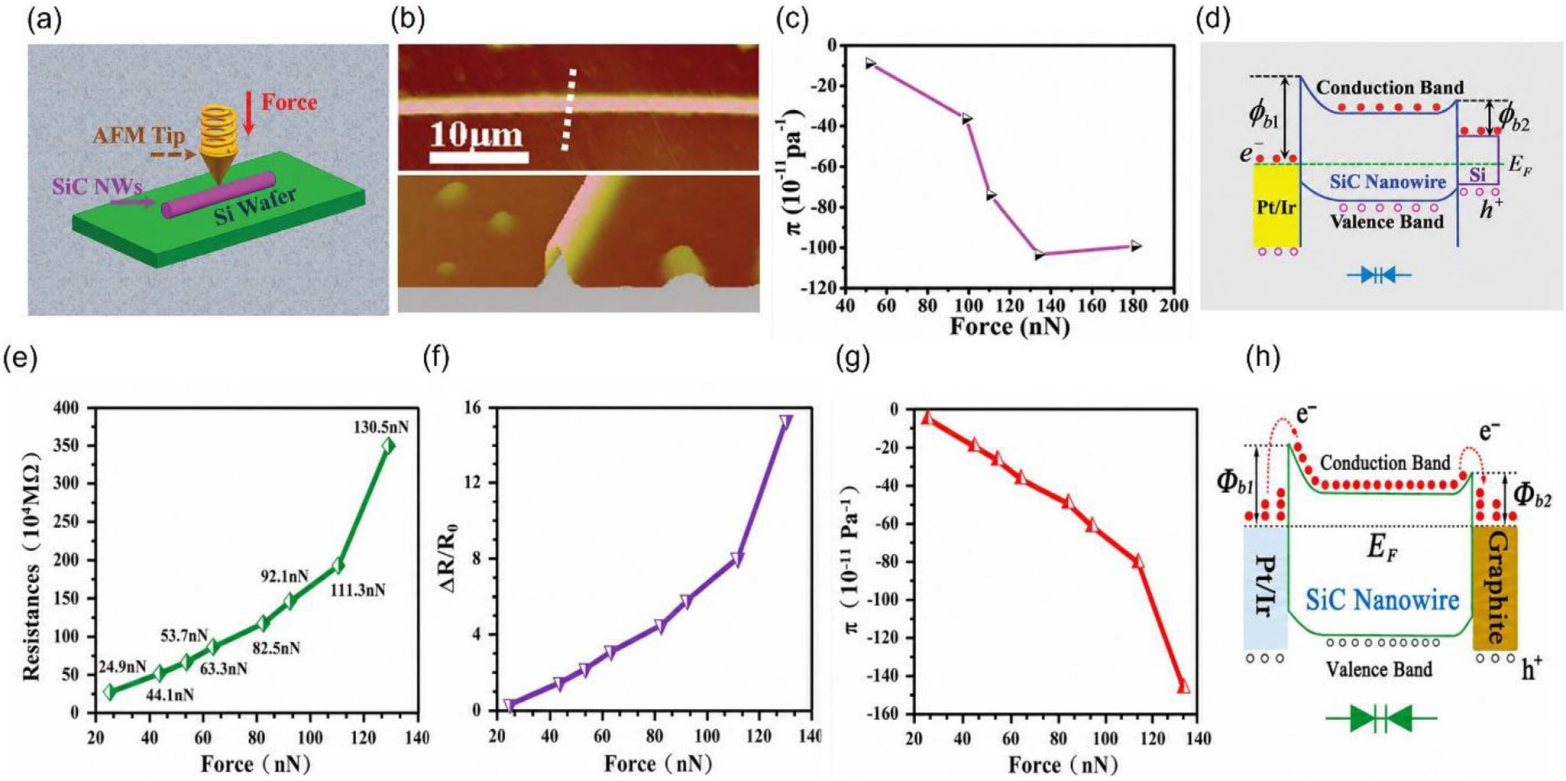
Giant negative piezoresistance in doped 3C‐SiC nanowires under nanoscale compressive loading (a–d: B‐doped; e‐h: N,P co‐doped). (a) Schematic of the conductive‐AFM measurement: a Pt/Ir tip applies a compressive force to a single SiC nanowire resting on a Si substrate. (b) Atomic force microscopy (AFM) topography images of the SiC nanowire (scale bar 10 µm). (c) Negative transverse piezoresistance coefficient π versus applied force for the nanowire on a Si substrate, decreasing from ≈ −8.83 to ≈ −103.42 × 10^−^^1^ ^1^ Pa^−^^1^ over ∼51.7–181.0 nN. (d) Metal–semiconductor–semiconductor band diagram of the Pt/Ir–SiC–Si junction, in which the Pt/Ir–SiC contact forms a Schottky barrier (φb1 ≈ 1.5 eV) and the SiC–Si contact a heterojunction (φb2 ≈ 0.85 eV), whose stress‐induced modulation underlies the response. The giant gauge factor of this B‐doped nanowire reaches −53.0 to −620.5. Reproduced/adapted with permission [54]. Copyright 2016, Royal Society of Chemistry. (e) Nanowire resistance as a function of applied AFM tip force on a graphite substrate, showing a monotonic resistance increase under increasing compressive loading. (f) Corresponding relative resistance change ΔR/R_0_ versus force, rising to ≈ 15.3. (g) Transverse piezoresistance coefficient π[1̄10] versus force, varying from −5.07 to −146.30 × 10^−^^1^ ^1^ Pa^−^^1^. (h) Band diagram of the Pt/Ir–SiC–graphite junction. The resistance increase under compression — that is, a negative piezoresistance, with a giant gauge factor up to −877.79 — is attributed to stress‐induced modulation of the Schottky barrier heights at the metal/SiC contacts. Reproduced/adapted with permission [53]. Copyright 2019, Royal Society of Chemistry.

Subsequent work by Cheng and co‐workers pushed this performance even higher by exploiting nitrogen and phosphorus co‐doping in SiC nanowires synthesized by polymer‐derived ceramic pyrolysis (Figure 6e–h) [53]. The co‐doping strategy was designed to introduce additional shallow donor states whose contribution to the carrier density and mobility could be modulated by strain, thereby amplifying the intrinsic resistivity term in the gauge factor expression beyond what single‐element doping could provide. The resulting nanowires exhibited a piezoresistance coefficient that varied continuously from −5.07 × 10^−^^1^ ^1^ Pa^−^^1^ at very low loading to −146.30 × 10^−^^1^ ^1^ Pa^−^^1^ at higher loading and reached a peak effective negative gauge factor of approximately −878, the largest value reported for any SiC nanowire at the time of publication [53]. More recent extensions of this approach have demonstrated that further architectural and doping refinements, including the use of B‐doped SiC nanobelts and core‐shell nanowire geometries, can push the negative gauge factor toward values of order −10^3^, suggesting that the absolute ceiling for nGF in SiC nanostructures has not yet been reached. Beyond nanowires, Nakamura et al. used first‐principles calculations to investigate piezoresistivity in SiC nanosheets [69], and Phan et al. measured the piezoresistive response of p‐type single‐crystalline 3C‐SiC thin film [70]. These representative gauge factors and piezoresistive responses across Si and SiC low‐dimensional structures are summarized in Table 1 [38, 60, 61, 62, 63, 64, 65].

**TABLE 1:** Representative gauge factors and piezoresistive responses of Si and SiC low‐dimensional nanostructures.

Material	Structure	Type / dopant	Size	Reported GF / key response	Reference / source
Si	Nanowire	p‐type	50–350 nm	Giant PZR π_l_ = −3550 × 10^−^^1^ ^1^ Pa^−^^1^; coefficient enhanced vs. bulk	He & Yang, 2006 [18]
Si	Ultrastrained nanowire	p‐type	∼100 nm	pGF → nGF transition; resistance decreases ∼10× at 3.5% tensile strain	Lugstein et al., 2010 [55]
Si	Nanowire	p‐type	—	Positive, negative, and giant PZR; π ≈ ‐—800–3000 × 10^−11^ Pa^−1^	McClarty et al., 2016 [56]
Si	Whisker	p‐type, B	—	GF ≈ 15 000 at 4.2 K	Druzhinin et al., 2015 [58]
3C‐SiC	Nanowire	p‐type, B	~540 nm	GF = −53.0 to −620.5	Li et al., 2016 [54]
3C‐SiC	Nanowire	—	150 nm	GF = −6.9 (PZR −1.15 × 10^−11^ Pa^−1^)	Shao et al., 2012 [68]
3C‐SiC	Nanowire	—	320 nm	GF = 14.1	Zeng et al., 2013 [65]
3C‐SiC	Nanowire	n‐type, N	~230 nm	PZR 0.75 𝑡𝑜 7.7 × 10^−11^ Pa^−1^	Bi et al., 2013 [62]
6H‐SiC	Nanowire	p‐type, Al	~170 nm	PZR 51.2 𝑡𝑜 159.5 × 10^−11^ Pa^−1^	Gao et al., 2011 [64]
SiC	Nanosheet / thin layer	—	3 nm thickness	—	Nakamura et al., 2011 [69]
3C‐SiC	Thin film	p‐type, Al	280 nm thickness	—	H. P. Phan et al., 2014 [70]
3C‐SiC	Thin film	p‐type, Al	245 nm thickness	GF ∼11	D. V. Dao et al., 2016 [63]
3C‐SiC	Nanowire	N/P co‐doped	∼260 nm	Gauge factors of longitudinal resistors: 9.5 to 12.5, Transverse resistors varied: ‐2 to ‐3.5	Cheng et al., 2019 [53]
3C‐SiC	Core–shell nanowire	SiO_x_ or Al_2_O_3_ shell	Al2O3‐shell SiCNW, core nanowire: 31nm shell: 342 nm, SiOx‐shell SiCNW, core nanowire: 40 nm, shell: 264 nm	GF tunable from −21 to 40; up to ∼100 with gate bias	Uesugi et al., 2021 [52]
SiC	Films / NWs / MEMS	various	various	Review of SiC GF values; high‐temperature MEMS context	Phan et al., 2015 [59]

Abbreviations: GF, gauge factor; MEMS, microelectromechanical systems. Dashes (—) denote values not reported; NW, nanowire; pGF/nGF, positive/negative gauge factor; PZR, piezoresistance; π, piezoresistance coefficient.

Despite strong metrics, Si and SiC nanowires are hard to contact and integrate as uniform films and share the band‐structure ceiling of bulk semiconductors; two‐dimensional semiconductors (MoS_2_, PtSe_2_, PdSe_2_; Section 2.3) instead strain‐tune the full bandgap over far larger ranges, enabling richer nGF mechanisms and easier soft‐sensor integration [19, 66, 67].

### 2.3 Atomically Thin Semiconductors: MoS_2_ and Strain‐Induced Bandgap Modulation

While silicon and silicon carbide nanowires demonstrate that low‐dimensionality can amplify the intrinsic negative piezoresistive response by orders of magnitude, the practical translation of these systems into flexible and wearable sensors remains constrained by the inherent difficulties of individually contacting and integrating nanowires across large device areas. Atomically thin two‐dimensional (2D) semiconductors offer a fundamentally complementary platform in which the active sensing layer is simultaneously mechanically compliant, scalable to large areas through chemical vapor deposition or wafer‐scale transfer, and—most importantly—possesses a band structure whose magnitude and character can be modulated by strain over a far wider fractional range than is possible in any bulk three‐dimensional crystal [12, 19, 57, 66, 71]. Among the many available 2D semiconductors, molybdenum disulfide (MoS_2_) has emerged as one of the most thoroughly studied systems, both because of the maturity of its synthesis and because its layer‐number‐dependent band structure provides a uniquely transparent platform in which strain‐induced bandgap modulation can be observed, quantified, and linked directly to the piezoresistive gauge factor [72].

The mechanistic foundation of negative piezoresistance in MoS_2_ rests on its highly strain‐tunable electronic structure, a property that was systematically characterized before the explicit piezoresistance measurements were performed. In a landmark study, Conley and co‐workers applied uniaxial tensile strain in the range of 0%–2.2% to monolayer and bilayer MoS_2_ flakes transferred onto flexible substrates and used a combination of Raman and photoluminescence spectroscopy to track the evolution of the optical bandgap [71]. They observed an approximately linear decrease in the optical bandgap of about 45 meV per percent strain for monolayer MoS_2_ and about 120 meV per percent strain for bilayer MoS_2_, accompanied at higher strains by a direct‐to‐indirect bandgap transition in the monolayer at roughly 1% strain. Subsequent work by other groups confirmed and extended these findings using biaxial strain, local indentation, and ultra‐large strain configurations, and established that the strain‐induced bandgap modification in MoS_2_ is robust, reproducible, and quantitatively predictable from first‐principles band‐structure calculations [66, 72, 73, 74, 75, 76]. Together these studies provide the essential mechanistic foundation on which any discussion of intrinsic nGF in MoS_2_ must build: the gauge factor of MoS_2_ is not the result of a small valley repopulation effect as in silicon, but rather of a continuous and substantial reshaping of the entire band structure under strain [66, 71].

The physical connection between this strain‐induced bandgap reduction and a negative gauge factor follows directly from semiconductor transport theory [66, 77]. In an undoped or lightly doped semiconductor, the intrinsic carrier concentration depends exponentially on the bandgap through the relation n ∝ exp(−E_g_ / 2k_B_ T), and the conductivity depends additionally on the effective mass and the carrier mobility, both of which are themselves functions of the underlying band structure [66, 77]. When tensile strain reduces the bandgap, the equilibrium carrier density increases and the effective mass typically decreases, both effects acting in the same direction to increase conductivity and therefore decrease resistance [57, 66, 71]. This is the qualitative origin of the intrinsic nGF in MoS_2_, and the magnitude of the effect is expected to scale with the magnitude of the strain‐induced bandgap shift, ∂E_g_/∂ε [66, 71]. Crucially, because the bandgap of MoS_2_ can be shifted by tens or even hundreds of millielectronvolts within a few percent strain—values orders of magnitude larger than the strain‐induced changes accessible in bulk silicon—the achievable gauge factor in 2D MoS_2_ is in principle comparable to or larger than that of nanostructured silicon, while being delivered in a film geometry that is naturally suited to flexible and wearable sensor integration [57, 66, 78].

A key experimental demonstration that this bandgap‐mediated negative piezoresistance is real, quantifiable, and layer‐number dependent was provided by Manzeli and co‐workers in 2015, as shown in Figure 7a–d [66]. In their study, mechanically exfoliated monolayer, bilayer, and trilayer MoS_2_ flakes were incorporated into a suspended nanoelectromechanical membrane configuration, in which uniform biaxial strain could be applied by pressurizing the back side of the membrane, and the resulting resistance change was simultaneously measured via four‐point I–V characterization. To extract the gauge factor unambiguously, finite‐element modeling was used to compute the spatially varying biaxial strain field across the membrane, allowing the locally averaged resistance change to be normalized by the locally averaged strain (Figure 7c,d). The resulting gauge factors were −148 ± 19 for monolayer MoS_2_, −224 ± 19 for bilayer MoS_2_, and −43.5 ± 11 for trilayer MoS_2_, all clearly negative and comparable in magnitude to those of state‐of‐the‐art bulk silicon strain sensors despite the entire sensing layer being only one to three atoms thick. These values are about two orders of magnitude larger than those obtained from strain sensors based on suspended graphene, in which the absence of a bandgap prevents the strain from accessing a comparable resistivity‐modulation mechanism. This gate‐tunability was further established by Tsai and co‐workers, who showed in flexible MoS_2_ field‐effect transistors that the negative gauge factor can be modulated by more than an order of magnitude through back‐gate biasing, reaching values up to approximately −40 in the subthreshold regime (Figure 7e–h) [76]. Complementary measurements on suspended few‐layer MoS_2_ ribbons under large uniaxial strain by Neri and López‐Suárez yielded gauge factors as high as 240, with the strain‐dependent conductance well described by a band‐gap‐closing model (Figure 7i–l) [75].

**FIGURE 7:**
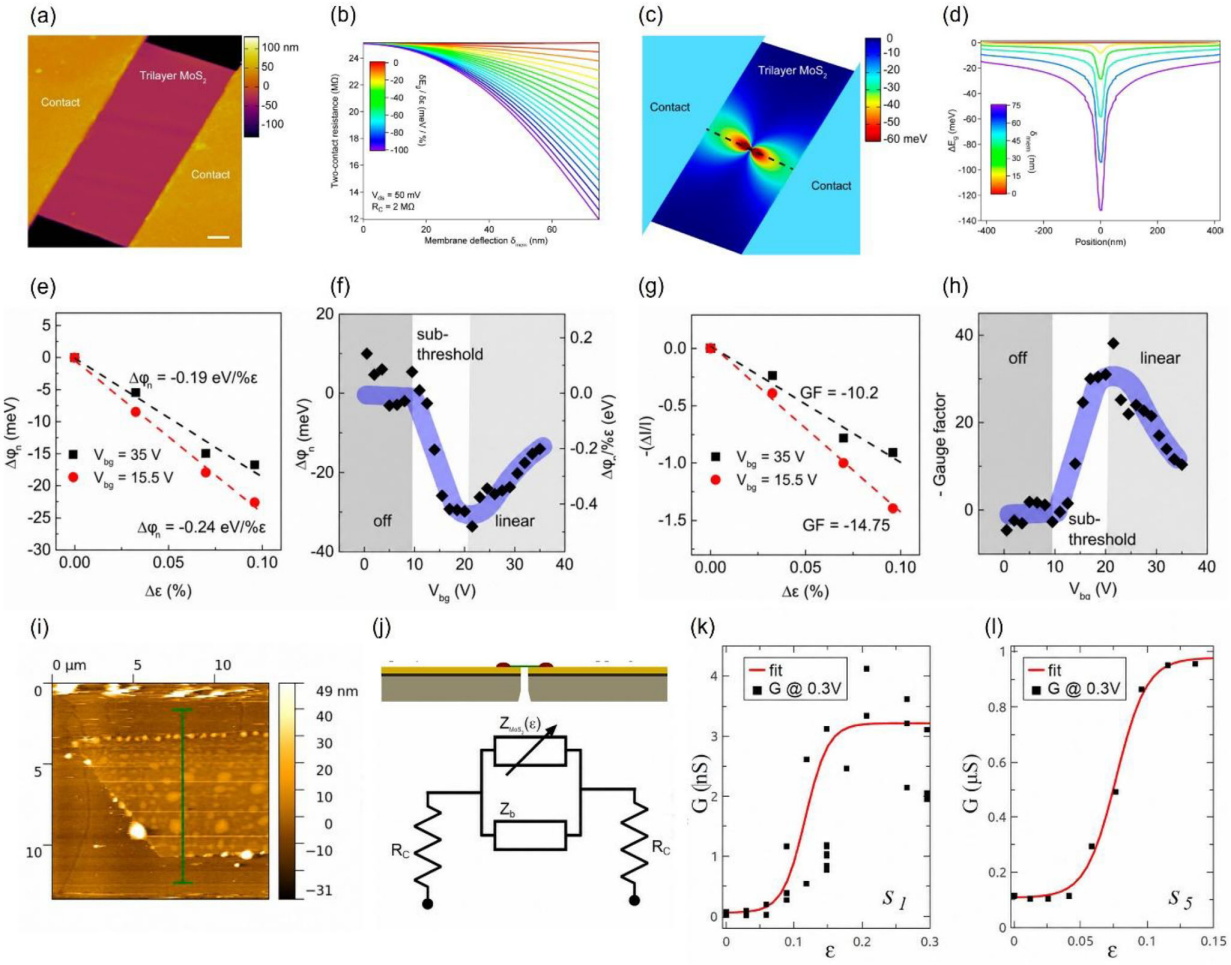
Strain‐modulated transport in atomically thin MoS_2_ and related 2D‐semiconductor devices. (a) AFM image of a contacted, suspended trilayer MoS_2_ membrane device. Scale bar is 200 nm. (b) Simulated resistance as a function of membrane deflection. (c) Finite element method (FEM) map of the strain‐induced band‐gap change ΔE_g_ across the deformed membrane. (d) ΔE_g_ profile along the dashed line in (c), with the largest band‐gap reduction near the indentation point [66]. Reproduced under the terms of the Creative Commons CC BY‐NC 4.0 License. Copyright 2015 The Authors, published by American Chemical Society. (e) Change in the (electron) Schottky‐barrier height Δφ_n_ of a flexible MoS_2_ field‐effect transistor (FET) as a function of applied strain. (f) Δφ_n_ as a function of back‐gate voltage V_bg_, showing that the strain response is gate‐tunable. (g) Relative current change −ΔI/I as a function of strain. (h) Gauge factor as a function of back‐gate voltage, demonstrating that the gauge factor is strongly gate‐tunable (sensitivity adjustable by more than an order of magnitude). Reproduced/adapted with permission [76]. Copyright 2015 American Chemical Society. (i) AFM image of a suspended few‐layer MoS_2_ ribbon. (j) Corresponding height profile. (k) Equivalent‐circuit model: two ohmic contact resistances R_C_, a background impedance Z_b_, and a strain‐dependent MoS_2_ impedance Z_MoS2_(ε). (l) Measured (V_p_ = 0.3 V) and modeled conductance for samples S_1_ and S_5_, yielding band‐gap modulation rates of ≈ −31 and −45 meV/% strain. Together, the panels show that tensile strain narrows the MoS_2_ band gap and, in gated devices, modulates the contact Schottky barrier — decreasing resistance and producing negative gauge factors whose magnitude depends on layer number and gate‐defined operating regime. Reproduced/adapted with permission [75]. Copyright 2018 American Physical Society.

The non‐monotonic dependence of the gauge factor on the layer number—with bilayer MoS_2_ producing the largest |GF|—is a particularly important observation, because it places direct experimental constraints on the microscopic mechanism [66], [71]. In monolayer MoS_2_, the bandgap is direct at the K point and is dominated by in‐plane orbital character, so that the strain‐induced bandgap change is determined primarily by changes in the in‐plane lattice constant. In bilayer and few‐layer MoS_2_, however, the bandgap is indirect and involves a substantial contribution from the out‐of‐plane Mo d_{z_^2^_}_ orbitals at the Γ point, which are particularly sensitive to the inter‐layer separation [66, 71]. When in‐plane tensile strain is applied, the Poisson contraction reduces the inter‐layer distance, which in turn modifies the Mo d_{z_^2^_}_–d_{z_^2^_}_ orbital overlap between adjacent Mo planes and produces an additional, geometry‐mediated contribution to the bandgap shift. Bilayer MoS_2_ benefits maximally from this effect because it contains exactly two Mo planes whose mutual interaction is strongest; in trilayer and thicker MoS_2_, the strain transferred to the inner layers is partially screened, and the average bandgap modulation per unit strain decreases. This layer‐dependent piezoresistive response therefore provides a microscopic fingerprint that the intrinsic nGF of MoS_2_ is genuinely band‐structure‐mediated rather than dominated by surface or contact effects, and that the response can be tuned through layer engineering.

The implications of the MoS_2_ piezoresistance results extend well beyond a single material system, and constitute the key conceptual bridge between intrinsic nGF in 2D semiconductors and the broader landscape of flexible and wearable strain sensors discussed in the later sections of this review. First, the fact that gauge factors of order −100 to −250 can be obtained in films only a few atoms thick demonstrates that intrinsic nGF is no longer restricted to either rigid bulk crystals or one‐dimensional nanowires, and is therefore directly compatible with the mechanical compliance required for soft sensing [66, 78]. Second, the demonstration that MoS_2_ retains its bandgap‐modulation behavior under repeated strain cycling—and that this behavior persists in larger‐area CVD‐grown films and in conformal tactile sensors integrated onto curved or non‐planar substrates—shows that the basic mechanism survives the practical fabrication and integration steps required to convert single‐flake measurements into device‐level platforms [66, 78]. Third, because the negative piezoresistance of MoS_2_ is fundamentally a property of its intrinsic band structure, it can in principle be transferred into composite or textile platforms by using MoS_2_ nanosheets as the active filler in a soft polymer matrix, a strategy that forms the central design principle for composite nGF discussed in Section 3 [25, 66].

Despite these strengths, several limitations of single‐flake MoS_2_ piezoresistance studies must be acknowledged, and they directly motivate the next steps in the materials development described in subsequent subsections [19, 66, 67]. The gauge factors quoted above are obtained from suspended, mechanically exfoliated, defect‐poor flakes operated in carefully controlled strain geometries; transferring this performance into wafer‐scale chemical vapor deposition (CVD) films often results in lower effective gauge factors due to grain boundaries, contamination, and contact‐resistance effects [66, 78]. In addition, the maximum elastic strain that monolayer MoS_2_ can sustain before fracture is much smaller than the strain ranges typically encountered in stretchable electronics and skin‐mounted sensors, so direct deployment of bare MoS_2_ films in highly deformable sensors is constrained [73, 78]. These considerations motivate the exploration of other 2D semiconductors with even larger ∂E_g_/∂ε—most notably the noble‐metal dichalcogenides PtSe_2_ and PdSe_2_, which are discussed in Section 2.4—and the use of 2D nanosheet networks dispersed in elastomeric matrices, which preserve the intrinsic nGF response while providing the mechanical compliance and processability required for real‐world soft strain and pressure sensors.

### 2.4 Noble‐Metal Dichalcogenides: PtSe_2_ and PdSe_2_ for Flexible nGF Sensing

While MoS_2_ established the conceptual framework for strain‐induced bandgap modulation as the origin of intrinsic negative piezoresistance in 2D semiconductors, its broader translation into practical sensor platforms remains limited by several material‐specific constraints: the requirement of high‐temperature chemical vapor deposition, the rapid degradation of monolayer films under ambient conditions, and the necessity of a polymer‐mediated transfer step that introduces wrinkles, contamination, and contact‐resistance heterogeneity. The noble metal dichalcogenides (NMDCs)—a subgroup of transition metal dichalcogenides in which the metal is replaced by a noble metal such as platinum or palladium—have emerged in recent years as a particularly attractive alternative for piezoresistive applications, because they retain the strong strain‐induced bandgap modulation that gives 2D semiconductors their large negative gauge factor while offering a combination of low‐temperature growth, ambient stability, and direct compatibility with both silicon complementary metal–oxide–semiconductor (CMOS) back‐end‐of‐line processes and flexible polymer substrates [19, 67, 79, 80]. Among the NMDCs, platinum diselenide (PtSe_2_) and palladium diselenide (PdSe_2_) have received the most attention as intrinsic nGF materials, and a coherent picture has now emerged in which these two compounds extend the bandgap‐modulation mechanism of MoS_2_ into a more scalable and integration‐ready material platform [24, 81, 82, 83].

An early demonstration of negative piezoresistance in PtSe_2_ was reported by Wagner and co‐workers in 2018, and is among the most cited references in the NMDC nGF literature [19]. As shown in Figure 8a–d, in their study, semimetallic PtSe_2_ films were grown by thermally assisted conversion of pre‐deposited platinum at a CMOS‐compatible temperature of 400°C, integrated into both a bending cantilever beam configuration and a freestanding poly(methyl methacrylate) (PMMA)/PtSe_2_ membrane structure, and measured under controlled mechanical loading [19]. The PtSe_2_ strain gauges fabricated in the cantilever beam configuration exhibited gauge factors as high as −85, clearly negative and substantially larger in magnitude than the gauge factors of commercial metal‐foil strain gauges (GF ≈ +2) (Figure 8a,b) [19]. When the same PtSe_2_ films were incorporated into nanoelectromechanical‐systems (NEMS)‐type pressure sensors with suspended membranes, the resulting devices reproducibly displayed pressure sensitivities that exceeded previously reported values by orders of magnitude per unit area, confirming both the magnitude and the sign of the intrinsic negative piezoresistive response [19]. The microscopic origin of the nGF in PtSe_2_ is qualitatively similar to that established for MoS_2_: tensile in‐plane strain reduces the effective bandgap and modifies the carrier transport, increasing conductivity and thereby decreasing resistance under elongation [71]. Density‐functional‐theory calculations confirm this mechanism directly, showing that in‐plane tensile strain increases the density of states at the Fermi level of semimetallic PtSe_2_, consistent with the experimentally observed decrease in resistance under elongation (Figure 8c,d). However, in PtSe_2_ the relevant electronic structure transition is more pronounced because the material undergoes a layer‐dependent semiconductor‐to‐semimetal transition, with monolayer PtSe_2_ exhibiting an indirect bandgap of approximately 1.2 eV while bulk PtSe_2_ is essentially semimetallic [19]. This thickness‐tunable electronic structure provides an additional design handle for negative gauge factor engineering that is not available in MoS_2_ [19, 80].

**FIGURE 8:**
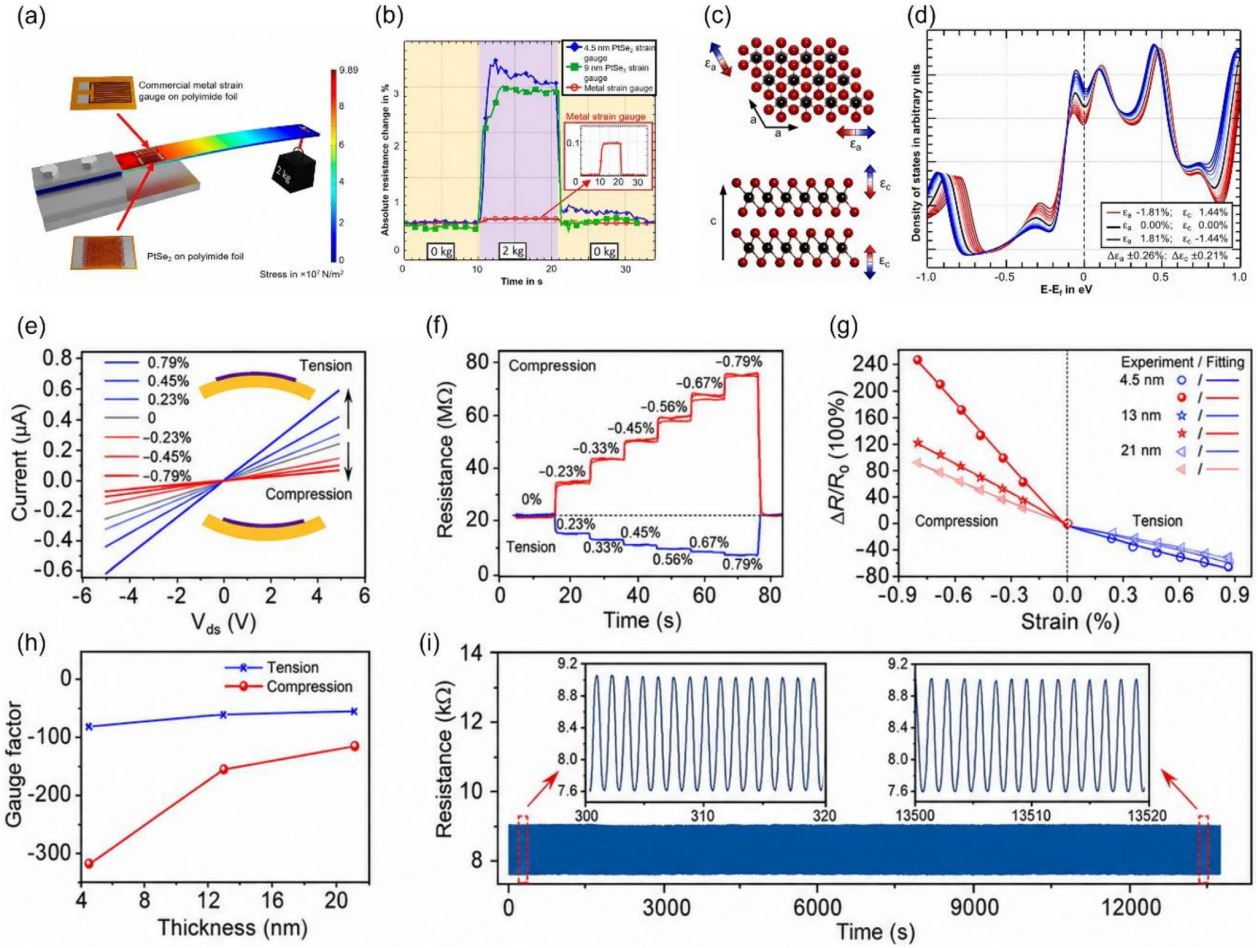
Intrinsic negative‐gauge‐factor behavior in PtSe_2_ and PdSe_2_ noble‐metal‐dichalcogenide strain sensors. (a) Bending cantilever‐beam setup comparing a PtSe_2_ strain gauge with a commercial metal strain gauge under a 2 kg load. (b) Absolute resistance change versus time (0 → 2 → 0 kg) for 4.5 nm and 9 nm PtSe_2_ gauges and a metal gauge, showing the much larger response of PtSe_2_ (gauge factor up to −85 at ε = 0.04%). (c) Atomic structure of bulk PtSe_2_ with the in‐plane (ε_a_) and out‐of‐plane (ε_c_) strain directions indicated. (d) Density functional theory (DFT) density of states near the Fermi level under combined in‐plane tensile and out‐of‐plane compressive strain, showing the increase in states at the Fermi level that underlies the negative gauge factor [19]. Reproduced/adapted under the terms of the Creative Commons CC BY 4.0 International License. (e) Current–voltage curves of a 4.5 nm PdSe_2_ film sensor under tensile and compressive strain (resistance decreases under tension, increases under compression). (f) Resistance versus time under stepwise tension and compression. (g) Relative resistance change ΔR/R_0_ versus strain for 4.5, 13, and 21 nm PdSe_2_ films. (h) Gauge factor versus film thickness under tension and compression, reaching a magnitude of ∼315 under compression and ∼−75 under tension for the 4.5 nm film. (i) Cyclic tension–release stability over more than 10^4^ cycles. Together, the panels show that strain‐induced band‐gap narrowing in layered noble‐metal dichalcogenides decreases resistance under tension, giving large intrinsic negative gauge factors [67]. Reproduced/adapted under the terms of the Creative Commons CC BY 4.0 International License.

The practical importance of PtSe_2_ was further reinforced by subsequent work that demonstrated direct growth of PtSe_2_ on flexible substrates without the need for any transfer step. Boland and co‐workers grew PtSe_2_ directly on polyimide foils using low‐temperature thermally assisted conversion of pre‐deposited Pt layers, and showed that the resulting strain gauges exhibit a robust negative gauge factor, withstand peel tests and water immersion, and remain functional through repeated cyclic loading [80]. More recent work by Lukas and co‐workers has further established that PtSe_2_ pressure sensors can be reproducibly integrated into CMOS application‐specific integrated circuit (ASIC) substrates with controlled membrane geometries and reliable high sensitivity, demonstrating that the path from a single 2D‐membrane laboratory device to a scalable, foundry‐compatible sensor platform is open [79]. Together, these works establish PtSe_2_ as a particularly integration‐ready nGF semiconductor, in which the intrinsic bandgap‐modulation mechanism that originally motivated 2D piezoresistance research is preserved while the material itself is delivered in a form that is air‐stable, scalable, and compatible with both rigid CMOS substrates and flexible polymer foils [19, 79, 80].

Palladium diselenide is a more recent and structurally distinctive addition to the NMDC nGF family, with several unique characteristics that distinguish it from PtSe_2_ both as a sensing material and as a model system [67]. Unlike PtSe_2_, which adopts a hexagonal 1T structure, PdSe_2_ crystallizes in a puckered pentagonal lattice with in‐plane anisotropy, in which each Pd atom bonds to four Se atoms and two adjacent Se atoms covalently bond with each other, producing a non‐centrosymmetric in‐plane structure that is markedly different from the layered structures of MoS_2_ and PtSe_2_ [67]. PdSe_2_ also exhibits a strongly layer‐dependent bandgap that varies from approximately 0 eV in bulk to 1.3 eV in the monolayer limit, providing a wide dynamic range over which strain‐induced bandgap modulation can produce large changes in carrier transport and therefore in resistance [67]. Critically, PdSe_2_ is remarkably air‐stable, allowing it to be used without the encapsulation strategies that are required for black phosphorus and many other anisotropic 2D semiconductors [67, 84, 85].

A recent demonstration of high‐performance piezoresistive sensing in PdSe_2_ was reported by Zhang and co‐workers in 2024 (Figure 8e–i) [67]. In their study, large‐area PdSe_2_ films were grown directly on polyimide substrates by plasma‐assisted selenization of sputtered Pd films at temperatures as low as 200°C, completely eliminating the conventional transfer step that has historically degraded the sensing performance of 2D‐based strain sensors and increased fabrication complexity. The resulting transfer‐free PdSe_2_/polyimide piezoresistive strain sensors exhibited gauge factors substantially larger than those of conventional metal‐foil strain gauges, with the negative sign of the response consistent with the strain‐induced bandgap modulation mechanism that is now well established across 2D semiconductors [19, 66, 67]. For the thinnest 4.5 nm films, the gauge factor reached a magnitude of approximately 315 under compression and about −75 under tension, with the device retaining a stable response over more than 10^4^ loading–unloading cycles and a response time below 25 ms (Figure 8e–i). In application demonstrations, the PdSe_2_ film sensors were used to detect human motion, voice vibrations, and arterial pulse signals when attached to the skin, and—when combined with deep‐learning analysis—were shown to enable the prediction of arterial temperature directly from pulse‐waveform measurements, with errors as small as two percent. These results illustrate that PdSe_2_ films grown directly on flexible substrates can serve simultaneously as the strain‐sensitive layer in a wearable bioelectronic device and as a substrate‐conformal element in a closed‐loop health‐monitoring system.

A parallel and conceptually important development in the NMDC field has been the demonstration that solution‐processed networks of exfoliated PtSe_2_ nanosheets retain the intrinsic negative gauge factor of their constituent 2D flakes, providing a direct bridge between the single‐film nGF measurements discussed above and the composite nGF systems that will be the subject of Section 3 [24, 25]. Ilhan and co‐workers performed electrochemical exfoliation of bulk PtSe_2_ into 2D flakes with aspect ratios exceeding 300, and assembled these flakes into solution‐processed networks deposited on flexible substrates [24]. The resulting networks exhibited a piezoresistive gauge factor of −5.45, smaller in absolute magnitude than the single‐membrane PtSe_2_ value of −85 but still clearly negative, demonstrating that the intrinsic nGF of PtSe_2_ can be transferred from the single‐flake regime into a scalable, processable, network architecture [19, 24]. Detailed modeling of the strain transfer between substrate and nanosheets indicated that the strain reaching the individual flakes was only about 8.5% of the macroscopically applied strain, implying that the apparent gauge factor reduction is largely a consequence of imperfect mechanical coupling rather than of a degradation of the intrinsic piezoresistive response of the constituent 2D material [24]. This insight is essential for understanding the broader landscape of 2D‐based composite nGF sensors, because it identifies strain transfer efficiency as a critical design parameter that, in principle, can be optimized through interfacial engineering, surface functionalization, and matrix selection.

Together these advances establish PtSe_2_ and PdSe_2_ as among the more developed noble‐metal 2D platforms for intrinsic nGF, sharing the bandgap‐modulation mechanism of MoS_2_ but with lower growth temperatures and better stability for CMOS and flexible‐foil integration, and retaining their intrinsic nGF from single flakes to nanosheet networks (Section 3). PdSe_2_‐on‐polyimide wearable health sensors further set a concrete application target for the later sections of this review [17, 67, 86].

## 3 Composite Negative Gauge Factor: Strain Transfer From Intrinsic nGF to Soft Matrices

### 3.1 Conceptual Framework: From Intrinsic to Composite nGF

The intrinsic nGF materials surveyed in Section 2—bulk silicon, Si and SiC nanowires, atomically thin MoS_2_, and noble metal dichalcogenide thin films—collectively establish that band‐structure‐mediated negative gauge factor behavior is robust and accessible across a wide range of material platforms. However, each of these single‐material systems shares a common set of practical limitations that restrict their direct use in soft electronic devices: the active sensing layer is rigid or brittle, the achievable elastic strain range is narrow, and the integration of individual nanowires or atomically thin membranes into device arrays remains challenging. The central question motivating the composite nGF literature is therefore conceptually simple: can the intrinsic nGF response of these materials be preserved while delivering the active sensing layer as a mechanically compliant, processable thin film or fabric [87, 88].

The composite nGF strategy that has emerged across the recent literature gives a strikingly affirmative answer to this question through a remarkably uniform design principle [24, 25, 88]. In a composite nGF system, an intrinsic nGF material—most commonly a 2D semiconductor nanosheet such as MoS_2_, WS_2_, or PtSe_2_, or in some cases a graphene layer or reduced graphene oxide flake whose effective gauge factor is engineered to be negative through interfacial coupling—is dispersed at or above the electrical percolation threshold within a soft polymer matrix or deposited as a conformal coating onto a textile substrate [24, 25, 88]. When the composite is mechanically strained, the deformation is transmitted from the macroscopic substrate, through the matrix, and ultimately onto the embedded filler nanosheets, where it modulates the intrinsic band structure in essentially the same way as in a free‐standing single‐flake measurement. The composite nGF response is therefore not a fundamentally new physical phenomenon, but a controlled transplantation of intrinsic nGF physics from a rigid single‐material platform into a soft, scalable composite architecture (Figure 9a–e) [24, 25, 88].

**FIGURE 9:**
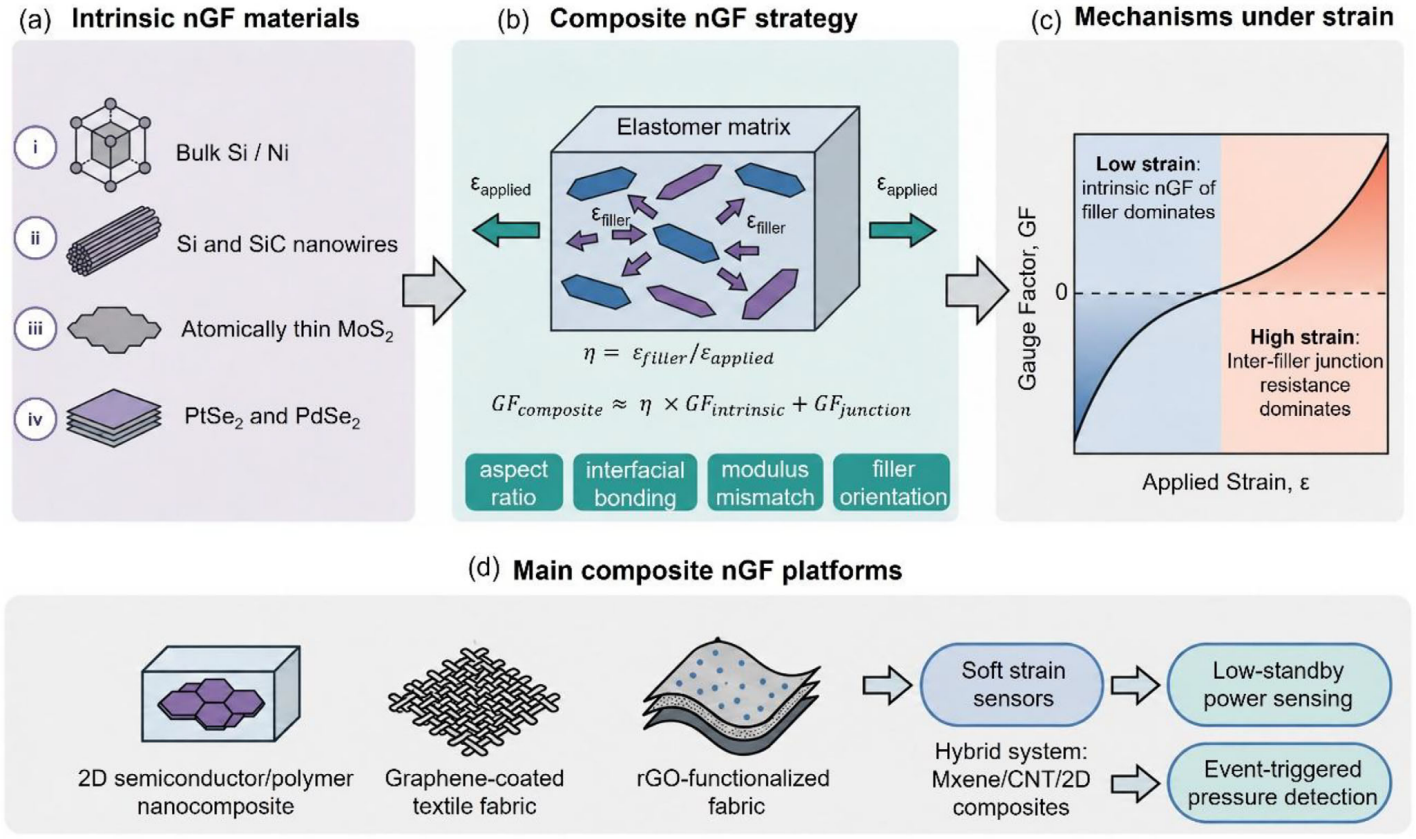
Transition from intrinsic nGF materials to composite nGF strategies. (a) Intrinsic nGF materials include bulk Si/Ni, Si and SiC nanowires, atomically thin MoS_2_, and PtSe_2_/PdSe_2_, where strain modifies the transport properties of the conductor it self. (b) Composite nGF strategies embed nGF fillers or strain‐sensitive conductive networks in an elastomer matrix. (c) Under low strain, the intrinsic nGF response of the filler can dominate, decreasing resistance; at high strain, inter‐filler separation and junction resistance often dominate, eventually producing a positive response or limiting the nGF window. (d) Composite performance depends on filler aspect ratio, orientation, interfacial bonding, modulus mismatch, and strain‐transfer efficiency. (e) Representative platforms include 2D‐semiconductor/polymer nanocomposites, graphene‐coated textiles, rGO‐functionalized fabrics, and hybrid MXene/CNT/2D composite systems.

The quantitative cornerstone of this picture is the strain transfer efficiency, denoted η, defined as the ratio of the strain experienced by an individual filler particle to the strain applied to the macroscopic composite, η = ε_filler_ / ε_applied_ [24, 25]. The effective gauge factor of a composite nGF system can be approximately expressed as GF_composite_ ≈ η × GF_intrinsic_ + GF_junction_, where the first term is the intrinsic nGF contribution scaled by the strain transfer efficiency and the second term is a generally positive contribution from changes in inter‐particle junction resistance [24, 25, 89]. The strain transfer efficiency itself depends on filler aspect ratio, interfacial bonding strength, modulus mismatch between filler and matrix, and the orientation distribution of the filler particles (Figure 9b). As a representative example, solution‐processed PtSe_2_ networks reported by Ilhan and co‐workers exhibited a measured η of approximately 8.5% despite the use of nanosheets with aspect ratios exceeding 300, demonstrating that the optimization of η remains a central open challenge [24]. This same principle explains why the absolute magnitude of the gauge factor in composite nGF systems—typically of order −2 to −25 in 2D‐semiconductor/polymer composites and −5 to −10 in graphene‐ or rGO‐based textiles—is consistently smaller than the intrinsic single‐flake values of order −100 to −250 reported in Section 2, even though the underlying physics is identical.

A second key feature of composite nGF systems, recognized explicitly by Biccai and co‐workers, is that the response is generally non‐monotonic with applied strain and reflects a competition between two opposing mechanisms [25]. At small strains, the dominant contribution is the intrinsic negative piezoresistance of the filler nanosheets, which is faithfully transferred to the composite through the strain‐transfer mechanism described above; the composite resistance therefore decreases with increasing strain, yielding a clear negative gauge factor [25]. As the applied strain exceeds a characteristic threshold—typically of order one percent—the inter‐nanosheet junctions widen significantly, the tunneling probability between adjacent flakes decreases, and the junction resistance contribution becomes dominant, producing a transition back to a positive gauge factor regime (Figure 9c) [25, 89]. Composite nGF systems are therefore naturally suited to low‐strain, high‐sensitivity sensing applications rather than to highly stretchable ones, a design constraint that aligns perfectly with the low‐standby‐power and event‐triggered sensing applications that motivate the entire negative piezoresistance framework of this review [17, 25, 86].

The remainder of Section 3 is organized around the two principal classes of composite nGF systems, presented in the order of increasing material complexity and applicational scope. Section 3.2 discusses 2D semiconductor/polymer nanocomposites, in which exfoliated MoS_2_, WS_2_, or PtSe_2_ nanosheets are dispersed in polymer matrices to produce thin‐film nGF sensors that directly validate the strain‐transfer framework. Section 3.3 turns to graphene‐coated textile fabrics, in which a continuous graphene network deposited on a woven substrate produces a flexible nGF e‐textile with a fundamentally different mechanical compliance profile from polymer‐based composites.

### 3.2 Two‐Dimensional Semiconductor/Polymer Nanocomposites

The most direct conceptual descendant of the intrinsic nGF physics discussed in Section 2 is the family of two‐dimensional semiconductor/polymer nanocomposites, in which exfoliated 2D semiconductor nanosheets such as MoS_2_, WS_2_, MoSe_2_, WSe_2_, or PtSe_2_ are dispersed at or above the percolation threshold within a soft polymer matrix to produce thin‐film strain sensors that exhibit clearly negative gauge factors. This category occupies the foundational position in the composite nGF literature for two reasons. First, it provides the cleanest experimental platform on which the strain‐transfer framework introduced in Section 3.1 can be quantitatively validated, because the constituent 2D semiconductors have well‐characterized intrinsic gauge factors from the single‐flake measurements of Section 2, and the polymer matrix can be chosen to provide controllable interfacial coupling and mechanical compliance. Second, the synthesis route—liquid‐phase exfoliation of bulk crystals into nanosheet dispersions followed by solution mixing with a chosen polymer—is fully solution‐processable, large‐area compatible, and amenable to standard thin‐film deposition techniques such as spin coating, spray coating, and inkjet printing, making this family of materials particularly well suited to the scalable fabrication of soft strain and pressure sensors. The seminal experimental demonstration and the quantitative theoretical framework that together define this subsection were both developed by the Coleman group at Trinity College Dublin, and the discussion below traces the progression of their work from the first explicit measurement of nGF in a 2D‐semiconductor/polymer composite to the predictive model that now underlies the design of next‐generation nanocomposite nGF sensors [24, 25, 89].

This first measurement, reported by Biccai and co‐workers in 2019, has informed much subsequent work in this category [25]. In this study, bulk MoS_2_ was exfoliated into nanosheet dispersions by liquid‐phase ultrasonication, and the resulting nanosheets were mixed with a polyethylene oxide (PEO) matrix at a range of filler volume fractions spanning the electrical percolation threshold and beyond (Figure 10a,b). A critical observation that motivated the choice of PEO as the matrix is that the polymer simultaneously serves two distinct roles(Figure 10c): it provides the mechanical compliance required for a soft, processable composite film, and it weakly electron‐dopes the MoS_2_ nanosheets, increasing the carrier density within each individual sheet and raising the overall composite conductivity to a level at which reliable resistance measurements can be performed even at moderate filler loadings (Figure 10d). Without this matrix‐mediated doping, percolating networks of pristine MoS_2_ nanosheets would exhibit conductivities too low to enable practical sensing; with the doping enabled by PEO, the resulting composites became conductive enough to function as transducers while retaining the intrinsic nGF response of the constituent nanosheets.

**FIGURE 10:**
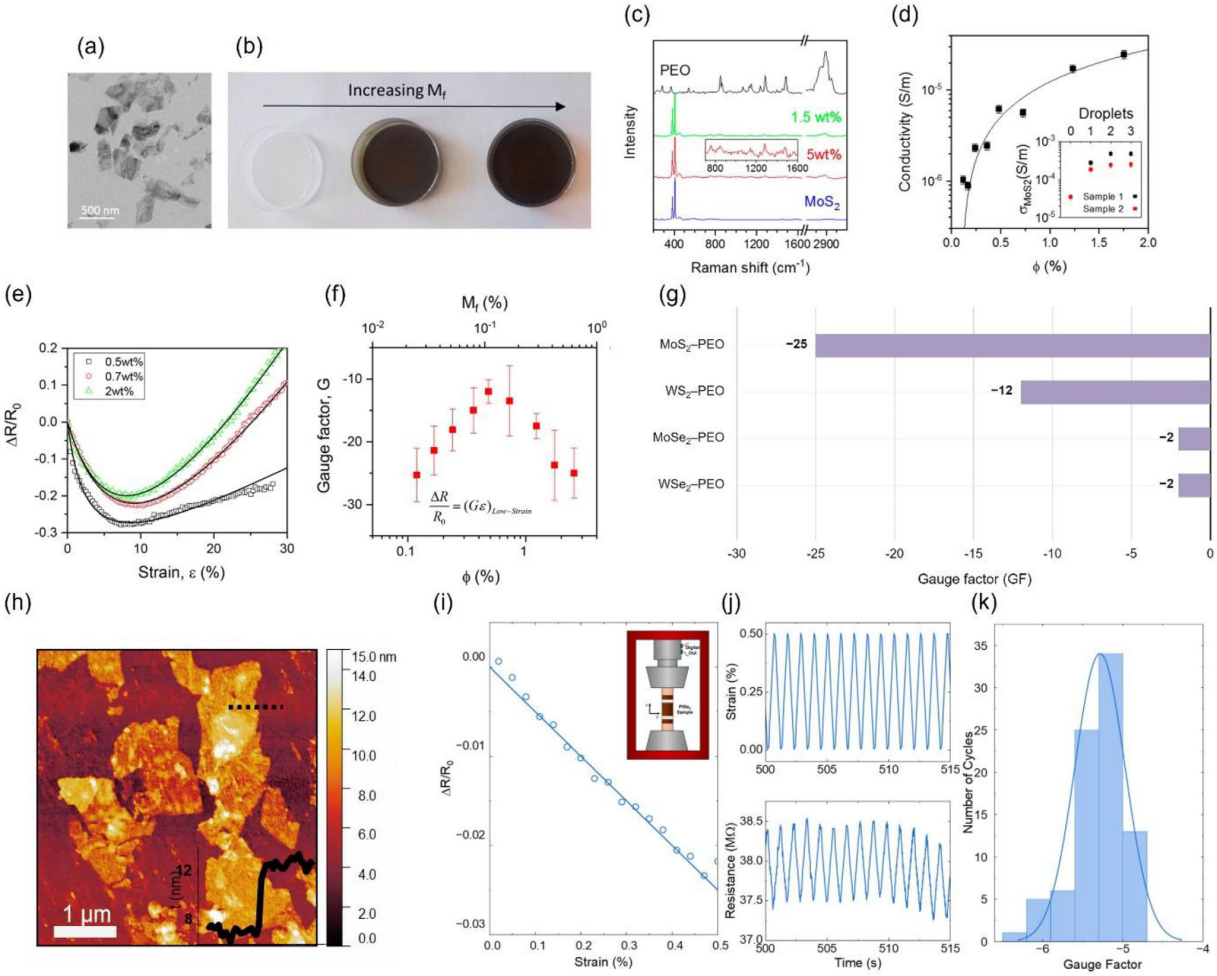
Negative gauge factor in 2D‐nanosheet/polymer composites and solution‐processed nanosheet networks. (a) Transmission electron microscopy (TEM) image of exfoliated MoS_2_ nanosheets. (b) Photographs of MoS_2_/PEO composite films with increasing MoS_2_ mass fraction Mf. (c) Raman spectra of PEO, MoS_2_, and the MoS_2_–PEO composite. (d) Composite conductivity as a function of MoS_2_ volume fraction φ. (e) Relative resistance change ΔR/R_0_ versus strain, showing the negative response at low strain. (f) Gauge factor as a function of filler loading φ. (g) Comparison of gauge factors for MoS_2_–PEO (≈ −25), WS_2_–PEO (≈ −12), and MoSe_2_–PEO / WSe_2_–PEO (≈ −2), showing the strongest negative response for MoS_2_–PEO. Reproduced/adapted with permission [25]. Copyright 2019 American Chemical Society. (h) AFM image of an electrochemically exfoliated PtSe_2_ nanosheet (apparent thickness ∼4 nm; scale bar 1 µm). (i) ΔR/R_0_ versus tensile strain for a Langmuir–Schaefer‐deposited PtSe_2_ network, showing the negative piezoresistive response with linear behaviour up to 0.5% strain. (j) Cyclic strain (top) and resistance (bottom) response under a 0%–0.5% sawtooth profile. (k) Histogram of gauge factors from repeated measurements, normally distributed about a mean of −5.45 ± 0.33, with the network remaining stable over more than 1000 cycles [24]. Reproduced/adapted under the terms of the Creative Commons CC BY 4.0 International License.

Under controlled tensile loading, the MoS_2_‐PEO composites exhibited a clearly negative gauge factor of approximately −25 in the small‐strain regime below the polymer yield strain (Figure 10e,f), confirming experimentally for the first time that the intrinsic negative piezoresistance established for atomically thin MoS_2_ in single‐flake measurements (Section 2.3) is preserved when the same material is incorporated into a percolating composite architecture. As the applied strain was increased beyond approximately one percent—the characteristic crossover regime where the yield strain of the PEO matrix is approached—the gauge factor underwent a smooth transition back to positive values, signaling that the junction resistance contribution from the widening of inter‐nanosheet gaps had begun to dominate over the intrinsic nGF response of the individual sheets (Figure 10e). This non‐monotonic strain dependence is precisely the qualitative behavior predicted by the conceptual framework introduced in Section 3.1 and provides the most direct experimental support for the picture in which composite nGF systems can be understood as the superposition of a strain‐transferred intrinsic nGF contribution and a positive junction‐resistance contribution that scales differently with applied strain.

The Biccai and co‐workers study went further by performing the same measurement on three additional 2D semiconductor systems—WS_2_, MoSe_2_, and WSe_2_—within the same PEO matrix, producing a comparative dataset that critically illuminates the underlying mechanism (Figure 10g). The WS_2_‐PEO composites exhibited a gauge factor of approximately −12, smaller in magnitude than that of MoS_2_‐PEO but still clearly negative, while the MoSe_2_‐PEO and WSe_2_‐PEO composites both exhibited gauge factors of approximately −2, almost an order of magnitude smaller in magnitude than the MoS_2_‐PEO baseline. The hierarchy of these gauge factors was attributed by Biccai and co‐workers not to differences in the intrinsic nanosheet gauge factors alone, but to the differing electrical conductivities of the nanosheet networks. As the nanosheet conductivity increases across the MoS_2_ → WS_2_ → MoSe_2_/WSe_2_ series, the inter‐nanosheet junction resistance increasingly dominates the composite resistance, so that strain‐induced changes in the negative nanosheet resistance have progressively less effect on the overall composite response. This conductivity‐dependent dilution of the intrinsic nGF, captured by their network model, identifies the nanosheet conductivity and the strain‐transfer efficiency—rather than the bandgap‐strain coefficient alone—as the key design parameters for composite nGF systems: a more conductive nanosheet yields a less negative composite gauge factor even when its intrinsic gauge factor is comparable to that of MoS_2_.

A subsequent and complementary contribution by Garcia and co‐workers in 2021 extended the empirical findings of Biccai et al. into a fully predictive model that quantitatively relates the composite gauge factor to the filler volume fraction and the composite conductivity, providing the first physical framework against which experimental nGF composite data can be systematically fit and optimized [89]. Their model treats the conductive nanocomposite as a percolating network whose macroscopic conductivity follows the standard scaling law σ ≈ σc,0 (ϕ − ϕc,0)^t above the percolation threshold ϕc,0, and decomposes the strain‐induced resistance change into a contribution from changes in the percolation parameters under strain and a contribution from the intrinsic piezoresistance of the individual filler particles [89]. The resulting analytical expression connects the experimentally measurable gauge factor G to the filler volume fraction ϕ, the composite conductivity σ, and a small number of physically meaningful parameters (the percolation threshold ϕc,0, the critical conductivity σc,0, the percolation exponent t0, and the intrinsic gauge factor of the filler). When fit to the experimental data of Biccai et al. and to additional measurements performed by Garcia and co‐workers themselves, the model was shown to reproduce both the magnitude of the gauge factor and its dependence on filler loading with quantitative accuracy, demonstrating that the composite nGF response can be predicted from a small number of well‐defined material parameters once the intrinsic gauge factor of the filler is known.

Two design implications of the Garcia model are particularly important for the present review. First, the analytical expressions for the composite gauge factor explicitly identify the percolation threshold ϕc,0 and the polymer‐induced doping of the filler (through its effect on σc,0) as the two most important experimental control parameters, providing concrete materials‐engineering targets for the optimization of composite nGF sensors. Second, the model demonstrates that the composite gauge factor depends on filler loading in a non‐monotonic way: at very low loadings the conductive network is too sparse to support reliable transport, at very high loadings the response becomes dominated by junction‐resistance changes that compete with the intrinsic nGF contribution, and there exists a well‐defined intermediate optimal loading regime in which the absolute gauge factor reaches its maximum. This insight rationalizes the relatively narrow range of filler loadings used in successful experimental demonstrations and provides explicit guidance for the development of next‐generation 2D/polymer nGF composites.

A third and conceptually important extension of the 2D‐semiconductor/polymer composite category, which already appeared in Section 2.4 in the context of NMDCs and which directly bridges Sections 2 and 3 of this review, is the solution‐processed PtSe_2_ network reported by Ilhan and co‐workers in 2025 (Figure 10h–k) [24]. Although the host material in this case is not a conventional flexible polymer but a substrate‐supported nanosheet network, the underlying experimental and conceptual structure is closely parallel to the Biccai‐Garcia framework: electrochemically exfoliated PtSe_2_ flakes with aspect ratios exceeding 300 were assembled into a percolating conductive network, the resulting film was deposited on a flexible substrate, and the macroscopic gauge factor of the network was measured under controlled tensile loading. The measured gauge factor of −5.45 is clearly negative, consistent with the intrinsic nGF of single‐flake PtSe_2_ established by Wagner and co‐workers (Section 2.4), and detailed mechanical modeling of the strain transfer between substrate and nanosheets indicated that the effective strain reaching the individual flakes was approximately 8.5% of the macroscopically applied strain. The combination of the Biccai‐Garcia framework for 2D/polymer composites and the Ilhan strain‐transfer analysis for solution‐processed networks therefore provides a unified quantitative picture in which the composite nGF of any 2D‐semiconductor system can be predicted from three measurable quantities—the intrinsic single‐flake gauge factor, the strain transfer efficiency, and the percolation parameters of the network—even as the precise material system and processing route vary widely.

Despite its maturity, the 2D‐semiconductor/polymer composite nGF approach faces open challenges that motivate alternative architectures: the negative‐GF regime typically holds only to ∼1–few % strain (above which junction resistance restores a positive GF), too narrow for tens‐of‐percent electronic‐skin platforms; cyclic durability under combined mechanical/environmental stress remains a concern; and the polymer matrix itself caps the achievable mechanical compliance and inter‐sheet coupling—motivating textile‐based architectures (Section 3.3), where the substrate provides compliance and fabric structure engineers the inter‐particle contacts.

### 3.3 Graphene‐Based Textile nGF Systems

The 2D semiconductor/polymer nanocomposites discussed in Section 3.2 demonstrate that the intrinsic negative piezoresistance of MoS_2_, WS_2_, and PtSe_2_ can be faithfully transferred into a soft polymer matrix, producing thin‐film nGF sensors whose performance is governed by the strain‐transfer framework and the percolation‐based gauge‐factor model of the Coleman group. A fundamentally different but complementary route to composite negative piezoresistance has emerged in parallel from the textile electronics community, in which graphene oxide is deposited onto woven or knitted fabric substrates (Figure 11a,b) and subsequently reduced to electrically conductive reduced graphene oxide (rGO) to produce a conformal coating that follows the surface of each individual fiber [87, 88, 90, 91]. In these graphene‐based textile architectures, the mechanical compliance and stretchability of the composite are provided not by the elastic deformation of a polymer matrix but by the structural reorganization of the textile yarns themselves, while the electrical conduction is provided by the conformal rGO layer that wraps the constituent fibers. This architectural distinction produces a mechanistically distinct family of composite nGF systems whose negative gauge factor originates from a combination of yarn‐level geometric reorganization, inter‐fiber contact enhancement, and inter‐flake hopping modulation, and which together constitute one of the most actively studied composite nGF platforms in the recent wearable‐electronics literature.

**FIGURE 11:**
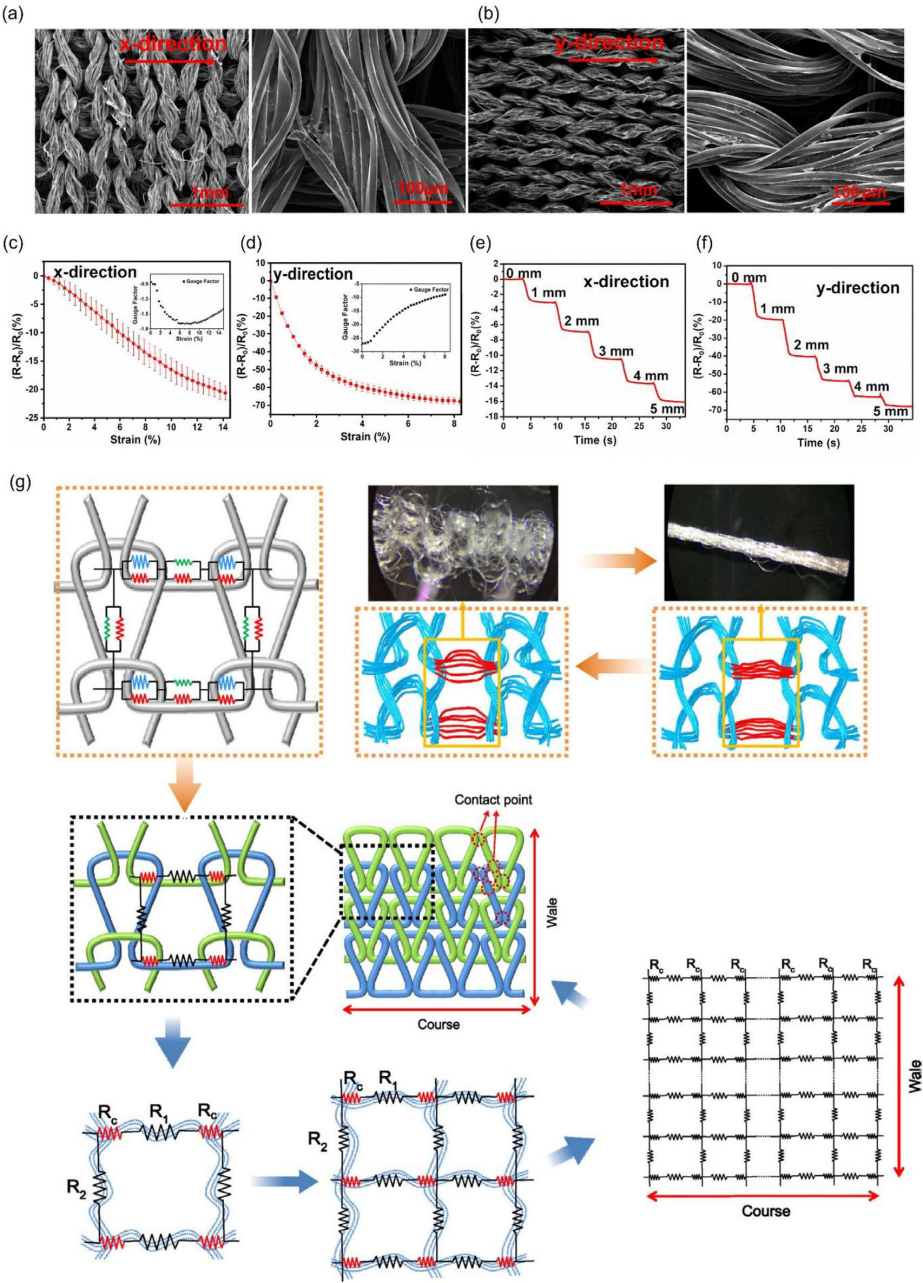
Direction‐dependent negative resistance variation and structure‐based mechanism in graphene/rGO textile strain sensors. (a,b) scanning electron microscopy (SEM) images of graphene‐coated textile strain sensors observed along the x‐ and y‐directions, showing the hierarchical knitted structure and conductive coating on individual fibers. (c,d) Relative resistance change, (ΔR/R_0_), as a function of tensile strain along the x‐ and y‐directions, respectively. The textile exhibits negative resistance variation in both directions, with a much larger response along the y‐direction, indicating strong structural anisotropy. (e,f) Stepwise stretching tests along the x‐ and y‐directions, showing stable and reversible decreases in resistance under incremental tensile deformation. Reproduced/adapted with permission [88]. Copyright 2018 American Chemical Society. (g) Schematic illustration of the knitted‐loop deformation mechanism, where stretching rearranges the loop geometry and increases fiber–fiber contact. Course–wale fabric geometry and contact‐point evolution in the rGO‐coated textile. Equivalent resistance‐network model composed of yarn resistances and contact resistances, explaining how tensile deformation lowers the overall resistance through improved conductive contacts. Reproduced/adapted with permission [87]. Copyright 2021 Springer Nature.

An early demonstration of negative gauge factor behavior in a graphene‐coated textile was reported by Yang and co‐workers in 2018, and continues to define the conceptual and methodological framework for this entire subfield (Figure 11a–f) [88]. In their study, polyester fabrics were dyed with graphene oxide dispersions using a procedure adapted from conventional textile coloration, then thermally reduced at high temperature to convert the graphene oxide into electrically conductive reduced graphene oxide while preserving the original fabric structure intact. The resulting graphene textile was a fully woven fabric in which each polyester fiber carried a conformal rGO coating, with no polymer encapsulation required, and was therefore mechanically indistinguishable from a conventional fabric in terms of bending, draping, and skin contact. When this graphene textile was subjected to uniaxial tensile strain, it exhibited a remarkable and reproducible negative resistance variation with increasing strain—a finding that was particularly notable in the context of contemporary wearable sensor research, in which the dominant piezoresistive textiles based on graphene or carbon nanotubes had previously been reported to display conventional positive gauge factor behavior dominated by crack opening, junction separation, or yarn elongation [11]. The Yang textile achieved this negative response while simultaneously maintaining excellent wearability, long‐term mechanical stability under repeated cycling, and intimate conformality to the contour of the human body—characteristics that are essential for the close‐fitting motion‐detection and physiological monitoring applications that motivated the work.

The microscopic origin of the negative gauge factor in graphene‐based textiles is conceptually distinct from that of the 2D semiconductor/polymer composites in Section 3.2, and was clarified in detail by Yang and co‐workers in a follow‐up study published in 2022 [88, 91]. Pristine graphene and reduced graphene oxide both exhibit only a small positive intrinsic gauge factor of order +2 to +6, originating from the strain‐induced reduction in Fermi velocity and the consequent decrease in carrier mobility under tensile loading [92]. If pure graphene or rGO were dispersed in a percolating network within a polymer matrix in the same way as MoS_2_ in the Biccai et al. study, it would therefore be expected to produce a positive gauge factor composite rather than a negative one [25, 88]. The negative gauge factor of the graphene‐based textile must therefore arise from a different mechanism that is specific to the fiber‐yarn‐fabric architecture, in which mechanical loading produces a coordinated structural reorganization of the textile network that overrides the intrinsic positive piezoresistance of the constituent rGO coating. Detailed measurements combined with in situ characterization of the tensile morphology, including the pioneering measurement of single fiber bundles, revealed that the dominant origin of the negative differential resistance is the relative displacement of fibers within the textile bundles under strain (Figure 11g). As the fabric is stretched, the constituent fibers within each yarn bundle shift relative to one another and come into closer contact, increasing the number of inter‐fiber contact points and the effective contact area while reducing the inter‐fiber junction resistance, so that the bundle resistance decreases even though the intrinsic gauge factor of the rGO coating on each individual fiber remains small and positive. The net effect is a clear negative gauge factor in the small‐to‐moderate strain regime, transitioning to a positive gauge factor at very high strains where the contact‐area enhancement saturates and individual fiber elongation begins to dominate [88, 91]. This mechanistic picture is consistent with the broader composite nGF framework of Section 3.1 in that the macroscopic negative response is produced by a coordinated reorganization of the composite architecture under strain, but it differs from the 2D‐semiconductor/polymer case in that the dominant resistance‐decreasing mechanism is geometric (contact area enhancement and yarn alignment) rather than electronic (intrinsic bandgap modulation).

A particularly important architectural extension of the graphene‐based textile nGF concept was reported by Han and co‐workers in 2021, who demonstrated that the negative resistance variation observed in conventional woven graphene textiles can be generalized to multiple loading directions through careful design of the textile knitting structure [90]. In their study, weft‐knitted graphene‐coated fabrics were prepared by electrospray deposition of reduced graphene oxide onto pre‐knitted polyester yarn substrates, producing a three‐dimensional knitted structure in which individual fibers were interlocked in two perpendicular directions rather than the single‐direction warp‐weft arrangement of conventional woven textiles. The resulting weft‐knitted graphene textile exhibited clear negative resistance variation not only under axial compression along the knitting direction—the loading mode in which graphene‐coated textiles had been previously characterized—but also under lateral tensile loading perpendicular to the knitting direction, indicating that the underlying yarn‐level structural reorganization mechanism is intrinsic to the textile architecture rather than to any specific loading geometry. This three‐dimensional nGF response has direct practical implications for wearable strain sensors, because it allows a single graphene textile patch to detect deformation along multiple axes simultaneously without the need for complex multi‐axis sensor arrays. More broadly, the Han study establishes that the textile architecture itself, not merely the choice of coating material or substrate fiber, is a central design parameter for composite nGF devices, and that engineering the three‐dimensional connectivity of the yarn network can produce qualitatively new sensing capabilities beyond what would be available in a simple two‐dimensional thin‐film composite.

A further and conceptually important evolution of the graphene‐based textile nGF concept involves the deliberate retention and functionalization of the residual oxygen‐containing groups on the rGO surface, exploiting the chemistry of partially reduced graphene oxide to enable simultaneously enhanced fiber adhesion, broader strain ranges, multimodal sensing capability, and integration with additional sensing functions within the same textile architecture [87, 93, 94, 95]. The decisive demonstration of this richer rGO‐based smart fabric concept was reported by Hong and co‐workers in 2021 (Figure 12), in a study that explicitly identifies and exploits the structure‐based origin of the negative piezoresistive response while expanding the operational envelope of textile nGF systems by approximately an order of magnitude [87]. In their work, polyester fabrics were dip‐coated with graphene oxide dispersions, dried, and then chemically reduced to rGO in a facile dip‐dry‐reduce procedure that preserved the original woven structure intact and produced a uniform conductive coating on each individual fiber (Figure 12a). The resulting rGO‐functionalized smart fabric exhibited a remarkable combination of mechanical and sensing characteristics that significantly extended the performance envelope established by previous graphene textile nGF systems (Figure 12b–e): the reversible strain‐sensing range reached up to 450% strain without fabric breaking—roughly an order of magnitude larger than the strain ranges typically accessed in pristine graphene textile nGF sensors—while the negative gauge factor measured at 10.7% strain was −5.92, with stable operation maintained over more than 1000 strain cycles (Figure 12f–i). The Hong sensor was also shown to quantitatively detect not only conventional tensile strain but also twist, shear, and poke deformation modes, with distinguishable response signatures for each loading mode, establishing the rGO‐functionalized smart fabric as a multimodal mechanical sensor rather than a single‐axis strain gauge. This combination of large reversible strain range, multimodal sensitivity, and clear negative gauge factor in a fully fabric‐compatible architecture has subsequently been cited as a representative benchmark for application‐oriented composite nGF wearable sensors [17, 87].

**FIGURE 12:**
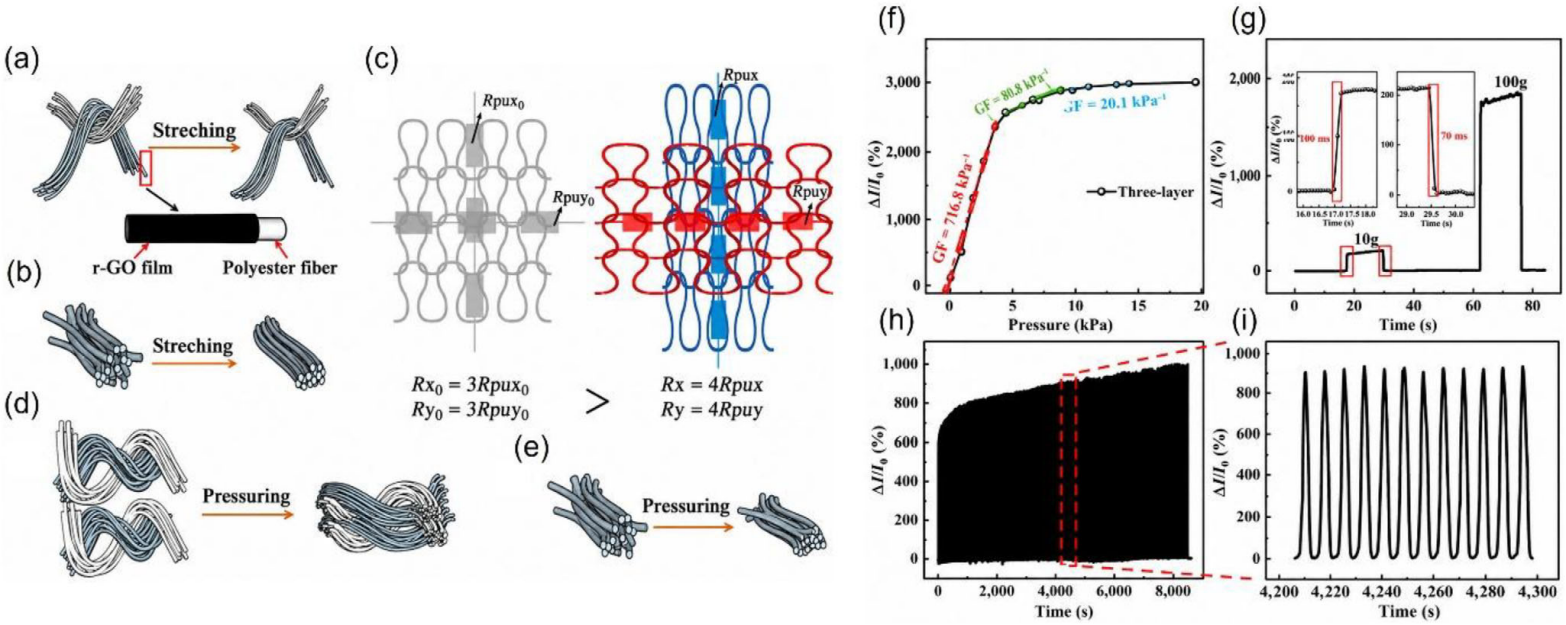
Structure‐based negative piezoresistivity in rGO‐coated smart fabric sensors. (a,b) Schematic illustration of the rGO‐coated polyester fiber and its deformation under tensile stretching, showing improved fiber–fiber contact in the conductive network. (c) Equivalent resistance model of the r‐GO textile under stretching, where Rpu denotes the resistance per unit length along the x‐ and y‐directions; despite the elongation, the total resistance decreases because Rpu drops as fiber–fiber contact improves. (d,e) Structural deformation of the knitted textile under pressing, illustrating contact‐area enhancement and resistance‐network reconfiguration. (f,g) Pressure‐dependent current response and loading/unloading behaviour of the multilayer rGO fabric sensor. (h,i) Long‐term cyclic pressure response and an enlarged view of repeated current modulation, demonstrating stable structure‐based negative piezoresistive behaviour. Reproduced/adapted with permission [87]. Copyright 2021 Springer Nature.

The richer functional palette enabled by the residual oxygen‐containing groups on rGO also opens routes to multimodal and multifunctional integration that are not readily available with pristine graphene textiles, and which have produced some of the most application‐relevant composite nGF demonstrations in the recent literature [93, 94, 96]. The hydroxyl, carboxyl, and epoxy groups on the rGO basal plane and edges provide direct chemical anchoring points for the incorporation of catalytically active metal nanoparticles, gas‐sensitive metal oxides, biorecognition elements such as antibodies or enzymes, and conducting polymers such as polyaniline or polypyrrole, each of which adds an independent sensing modality without disrupting the underlying composite nGF strain response [87, 93, 94]. Reduced graphene oxide‐coated linen fabrics have been demonstrated as combined gas and strain sensors capable of detecting both volatile organic compounds and tensile deformation through orthogonal electrical signal channels, while rGO/polydopamine/carbonized cotton fabric composites have been demonstrated as broad‐range strain sensors with workable strain ranges of 0%–120% and superb cyclic durability over 800 cycles [93, 96, 97]. These multifunctional rGO smart fabrics establish that the composite nGF concept is naturally compatible with the broader requirements of next‐generation wearable electronics, in which a single textile substrate must simultaneously perform mechanical sensing, environmental monitoring, and physiological signal acquisition while remaining comfortable, durable, and machine washable.

Comparison between the graphene‐based textile nGF systems discussed in this subsection and the 2D‐semiconductor/polymer nanocomposites of Section 3.2 reveals a complementary set of strengths and trade‐offs that has direct implications for the design of next‐generation composite nGF sensors. In the polymer‐based composites of Section 3.2, the negative gauge factor originates from a well‐understood intrinsic electronic mechanism whose magnitude is set by the strain‐induced bandgap modulation of the constituent 2D semiconductor, and whose dependence on filler loading, conductivity, and strain magnitude can be described quantitatively by the Garcia model; the principal limitation is the narrow strain range over which the response remains negative, typically below approximately one percent [25, 89]. In the graphene‐based textile composites of the present subsection, by contrast, the negative gauge factor originates from a geometric architecture‐mediated mechanism that operates over a substantially wider strain range—commonly up to ten percent in pristine graphene textiles and several hundred percent in chemically engineered rGO smart fabrics—because the underlying yarn reorganization process is mechanically continuous and does not saturate at the small‐strain percolation threshold of a polymer/filler system. The trade‐off, however, is that the magnitude of the negative gauge factor accessible in graphene‐based textiles is typically smaller in absolute terms than that of the best 2D‐semiconductor/polymer composites, because the underlying mechanism relies on the relatively weak geometric modulation of inter‐yarn contacts rather than on the strong electronic modulation of an intrinsic bandgap. In practical wearable applications, this trade‐off generally favors textile‐based nGF systems for large‐strain monitoring of human body motion, joint flexion, and respiration, and 2D‐semiconductor/polymer composites for high‐sensitivity detection of subtle pressure and physiological vibrations such as arterial pulse and voice.

## 4 Polymer Chain Reorganization nGF

### 4.1 Conceptual Framework

Whereas the composite systems of the preceding section produce nGF as a network phenomenon — a filler dispersed in a matrix, with strain modifying the inter‐particle network — this section concerns a fundamentally different scenario in which the conductor and the medium are one and the same. A single‐component conducting polymer film can decrease in resistance as it is stretched, not because particles rearrange within a matrix but because the polymer itself reorganizes: conducting domains grow, chains align, crystallites bridge, or localized states overlap more strongly under deformation. This morphology‐driven, single‐component response defines the polymer‐reorganization class.

Three observations are useful for orienting the discussion that follows. First, nGF in conducting polymers was recognized long before the recent interest in stretchable electronics: Naarmann's 1987 demonstration that mechanically drawn polyacetylene reaches conductivities above 20 000 S cm^−^^1^ along the draw direction [98] is a static endpoint of the same physics — stretching aligns chains, reduces disorder, and lowers resistance — with the 1990s–2000s literature reporting comparable draw‐induced conductivity gains; the modern nGF literature adds in situ measurement on compliant substrates that resolves the strain–resistance trajectory, reversibility, anisotropy, and substrate coupling. Second, within the conducting‐polymer field the existence of nGF is now treated as established rather than anomalous.

Recent reviews and primary studies frequently state, almost as an aside, that PEDOT:PSS is the principal exception among conducting films on flexible substrates whose resistance decreases rather than increases under strain [99], and that polyacetylene, polyaniline, and P3HT all show resistivity decreases upon stretching due to alignment‐driven conformational change [99]. The third observation is that the boundary between single‐component conductors and filler‐in‐matrix composites is genuinely fuzzy. A PEDOT:PSS film printed onto cotton twill is a single‐component conductor in the sense that no second conductive phase is added, but the strain transfers principally through the woven yarn architecture and the polymer's role is to provide a deformable conductive coating on the fibers. Such systems are best treated as boundary cases in which both polymer‐reorganization mechanisms and network mechanisms (improved fiber–fiber contact) operate in series. The framework adopted below does not artificially partition these cases; it follows the dominant physics of each report.

The conceptual scaffold for understanding nGF in single‐component conducting polymers rests on the recognition that essentially all of the conducting polymers of interest — PEDOT:PSS, polyaniline, polypyrrole, polythiophene — are morphologically heterogeneous on the nanometre scale. Within a typical film one finds dense regions of conjugated, doped polymer (PEDOT‐rich cores, PANI emeraldine salt domains, ordered PPy grains) embedded in or interleaved with a less conductive matrix (excess PSS shells, undoped PANI, amorphous PPy). The conductivity contrast between these two phases is typically three to five orders of magnitude, with the doped conducting domains exhibiting metallic or near‐metallic behavior and the insulating shells acting as barriers for charge transport between adjacent domains. The morphology of these phase‐separated systems has been characterized extensively by AFM phase imaging, grazing‐incidence X‐ray scattering, and transport spectroscopy, with the canonical picture for PEDOT:PSS being that of a “pancake” or “lasagna” structure in which approximately 20–80 nm PEDOT‐rich cores are partially surrounded by PSS shells of variable thickness, with the conducting cores connected through 20‐nm‐wide channels at 5%–10% of the core circumference [100, 101]. Charge transport across this disordered network is, in the great majority of cases, well described by Mott's variable range hopping (VRH) formalism, with

$$\rho \left(T\right)=\rho_{0}\exp\left[{\left(\frac{T_{0}}{T}\right)}^{\alpha }\right]$$

where α = 1/(1 + D) for D‐dimensional hopping and T_0_ scales inversely with the localization length ξ and the density of states at the Fermi level *N*(*E*_F_) according to T_0_ ∝ 1/[*N*(*E*_F_)ξ^3^] for 3D VRH. The localization length ξ itself can be expressed as ξ = ad/s, where a is the tunneling decay length in the insulating barriers, d is the conducting‐domain diameter, and s is the barrier thickness.

The localization length is, in this picture, the parameter most directly coupled to the polymer's morphology: it grows with the size of conductive domains, increases as the insulating barrier between domains narrows, and depends on the degree of intra‐chain conjugation in the conducting phase. Any strain‐induced morphological change that increases ξ — by growing the cores, by improving inter‐domain coupling, by bridging gaps with new crystallites, or by squeezing localized states into stronger overlap — produces an exponentially amplified decrease in ρ, since the resistivity depends exponentially on (T_0_/T)^α^ and T_0_ depends inversely on ξ^3^ for 3D VRH. This exponential amplification is the deep reason why even modest strain‐induced morphological changes can produce large changes in resistance: a 50% increase in localization length translates, in a 3D VRH system at fixed temperature, into a reduction of T_0_ by a factor of approximately 3.4 and an attendant exponential reduction in ρ that can easily exceed an order of magnitude. This single principle accounts, with appropriate specialization, for the great majority of these nGF observations reported to date. The differences between systems lie not in the principle but in which morphological feature is the rate‐limiting step for transport and in how strain modifies that feature.

Within the literature surveyed in the remainder of this section, five distinct mechanisms are identifiable (Figure 13a–e), and they are not mutually exclusive: a single film may exhibit one mechanism at small strains and a different mechanism at large strain, or two mechanisms operating in parallel with different time scales. Section 4.2 examines the PEDOT:PSS literature, which provides the most thoroughly characterized examples of two of these mechanisms — domain/chain alignment at small strain and core coalescence at large strain — together with the strain‐insensitive systems that illuminate the balance between morphological and geometric contributions. Section 4.3 then surveys the parallel observations in polyaniline, polypyrrole, and conducting‐polymer‐coated textiles, which introduce three additional mechanisms (crystallite bridging, surface‐state hopping, and yarn‐architecture‐mediated nGF) and which collectively establish that nGF is a general phenomenon of conducting polymers rather than a peculiarity of any single system.

**FIGURE 13:**
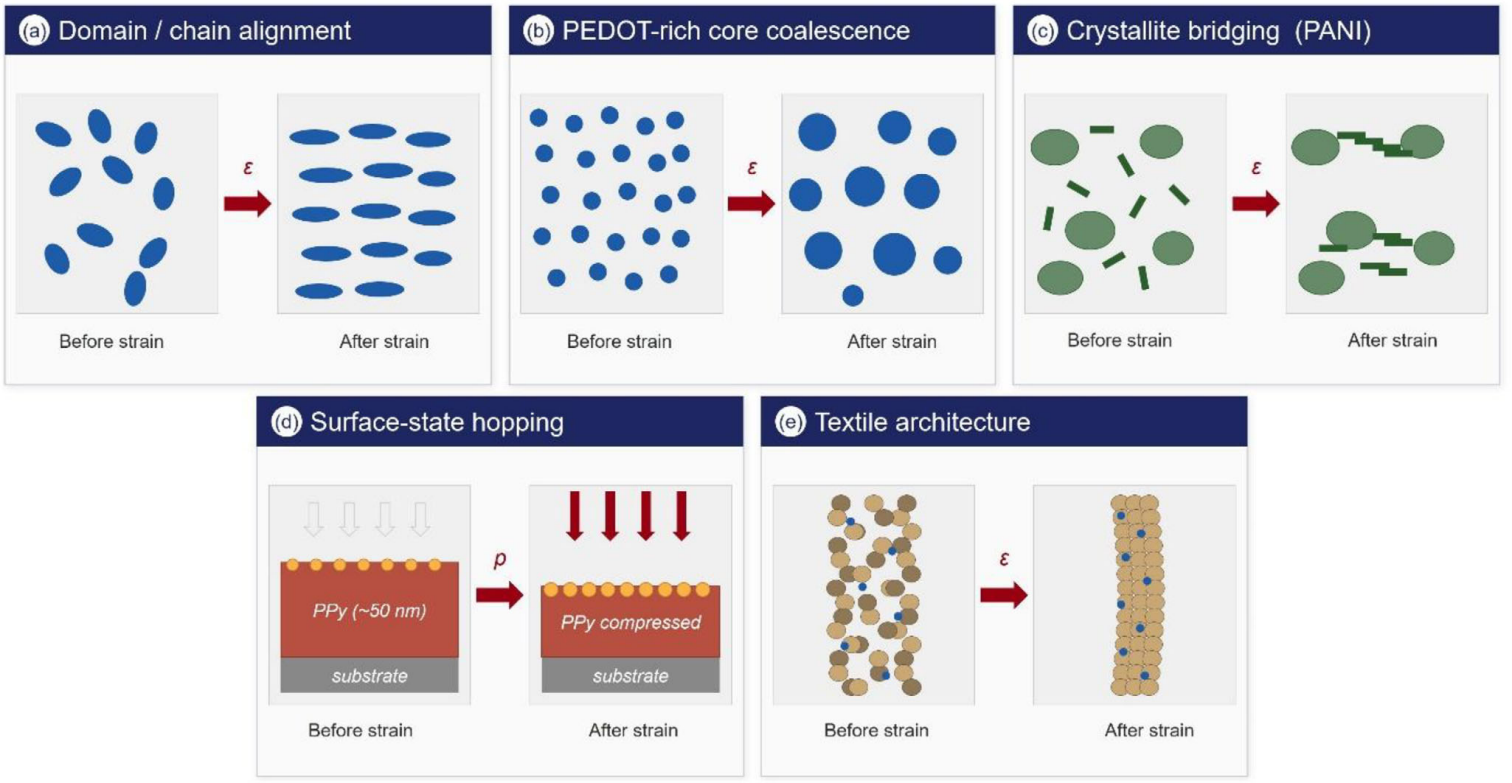
Five mechanisms of polymer‐chain‐reorganization nGF. (a) Domain or chain alignment under strain improves charge transport along the stretching direction. (b) PEDOT‐rich‐core coalescence (52 → 89 nm) increases the effective conductive‐domain size and reduces hopping barriers. (c) Crystallite bridging in polyaniline connects conducting domains during deformation. (d) Surface‐state hopping in ultrathin polypyrrole films (< 70 nm) can be enhanced by compression or interface‐mediated deformation. (e) Textile architecture combines a conducting‐polymer coating with yarn‐level reorganization, allowing tensile strain to improve conductive contacts. These five routes define the polymer chain reorganization nGF as a morphology‐driven response in conducting polymers and polymer‐coated structures.

### 4.2 nGF Mechanisms in PEDOT:PSS Systems

One of the more thoroughly characterized of these mechanisms is domain or chain alignment, in which strain biases the polymer's conductive domains to align preferentially along the loading direction and thereby improves intra‐domain charge transport along that direction at the expense of perpendicular transport. The clearest in situ demonstration is the 2011 study by Vijay, Rao, and Narayan on pristine PEDOT:PSS films deposited on styrene‐ethylene‐butylene‐styrene (SEBS) elastomeric substrates [102]. On non‐SEBS substrates (natural rubber, nitrile, poly(dimethylsiloxane) (PDMS)) those authors observed prompt film delamination and cracking at strains of 2%–5%, but on SEBS the PEDOT:PSS film maintained mechanical integrity through 28% strain without visible cracks. Within this crack‐free window, the surface resistance as a function of strain showed a characteristic non‐monotonic behaviour: a clear minimum of approximately −15% in R∥ at a strain of approximately 14%, followed by an increase at higher strains. The parallel and perpendicular resistances evolved in opposite directions, producing a strain‐induced electrical anisotropy that increased monotonically up to ∼10% strain. Vijay and coworkers modelled this response using a differential effective medium approximation (DEMA) in which spherical PEDOT‐rich granules embedded in a PSS matrix dynamically deform into prolate ellipsoids under strain. Treating the granule boundary area as the rate‐limiting step for transport and enforcing volume conservation, the model predicts σ∥(λ) ∝ λ^2^ and σ⊥(λ) ∝ 1/√λ for the conductivity parallel and perpendicular to the strain ratio λ, in excellent agreement with the experimental data below 15% strain. Temperature‐dependent measurements supported the alignment interpretation: the conductivity of unstrained films followed Mott's 0.5 exponent characteristic of disordered conducting polymers, while strained films exhibited a markedly weaker temperature dependence, consistent with a reduction in disorder.

As shown in Figure 14e–g, the same alignment mechanism reappears in a more recent and more dramatic form in the work of Volkert et al. [104]., who studied transfer‐printed PEDOT:PSS films on poly(vinyl alcohol) substrates infused with 15–55 wt% glycerol as a plasticizer. The glycerol diffuses from the substrate into the PEDOT:PSS layer, where it triggers a reorganization that brings PEDOT domains into closer contact and substantially improves intrinsic conductivity. Upon stretching, the films exhibit a three‐regime trajectory: an initial plateau, a deep minimum in sheet resistance occurring at strains between 65% and 144% (depending on the glycerol loading), and an increase at still larger strains. At the minimum, the sheet resistance falls to approximately one‐fifth of its initial value, and the films elongate crack‐free up to 160%. The authors attribute the resistance minimum to chain alignment in the plasticized PEDOT:PSS, with the plasticizer acting both to enable large‐strain deformability and to facilitate the alignment process by lowering the local viscosity of the polymer matrix. The strain at which the minimum occurs systematically shifts to higher values as the glycerol content increases, consistent with a kinetic interpretation in which more plasticized films require larger strains to fully align their domains before the geometric resistance increase begins to dominate. The Volkert result extends Vijay's 0%–15% strain window by an order of magnitude and demonstrates that, with appropriate plasticization and substrate engineering, the alignment‐driven nGF regime can persist to strains directly relevant to wearable applications.

**FIGURE 14:**
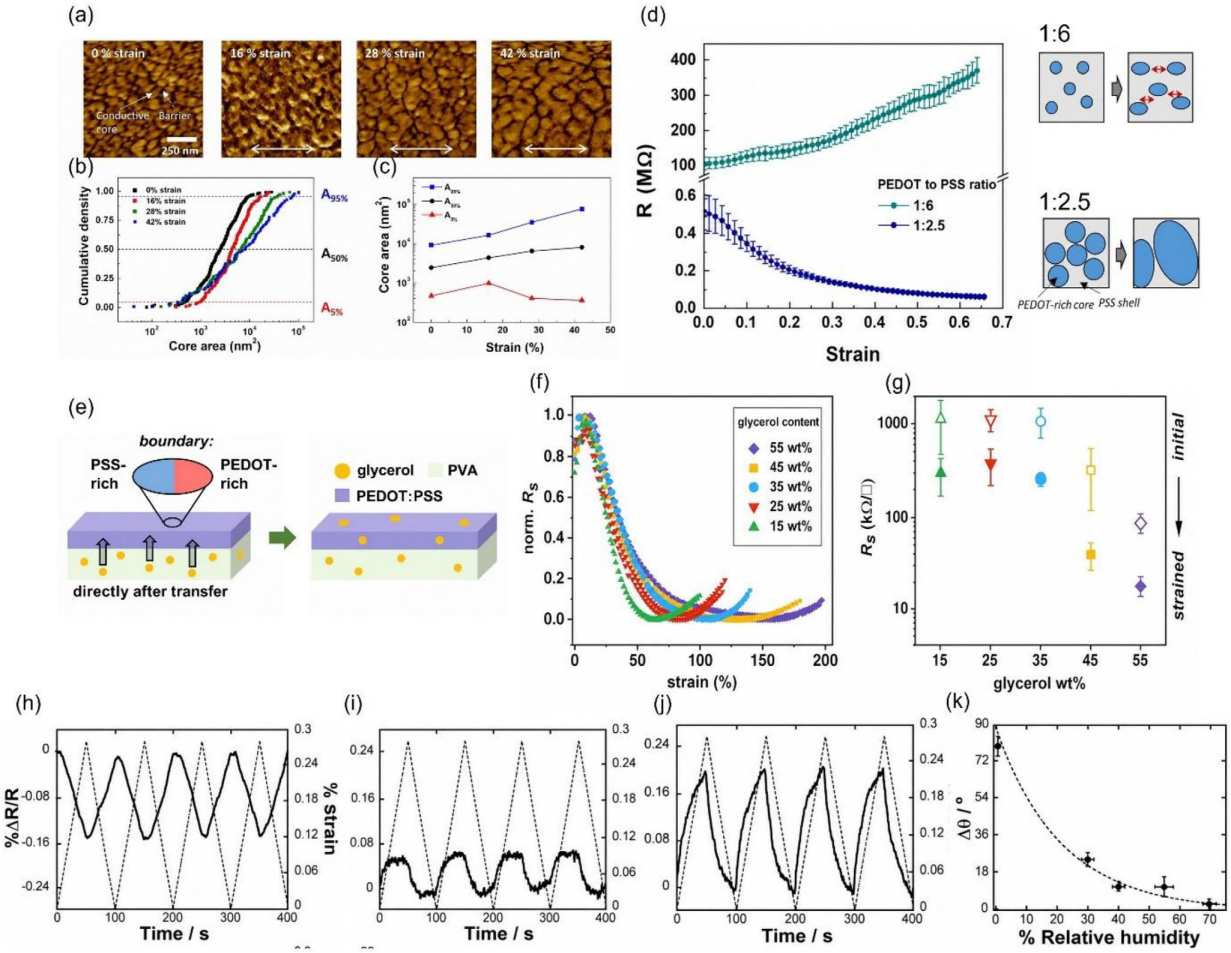
Polymer‐chain/domain reorganization mechanisms underlying negative piezoresistive responses in PEDOT:PSS. (a) AFM phase images of PEDOT:PSS films at 0%, 16%, 28%, and 42% strain, showing strain‐induced growth and coalescence of conductive PEDOT‐rich cores (bright) surrounded by insulating PSS shells/barriers (dark). (b) Cumulative‐density distribution of PEDOT‐rich core area at each strain. (c) Characteristic core areas (A5%, A50%, A95%) versus tensile strain, confirming conductive‐domain enlargement. (d) Resistance–strain behaviour for two PEDOT:PSS compositions with their core/shell schematics: the PEDOT‐rich 1:2.5 film shows a strong resistance decrease, whereas the PSS‐rich 1:6 film gives a positive response [103]. Reproduced/adapted under the terms of the Creative Commons CC BY 4.0 International License. (e) Schematic of glycerol diffusion from a poly(vinyl alcohol) (PVA)/glycerol substrate into PEDOT:PSS, modifying the PEDOT‐rich/PSS‐rich boundary. (f) Normalized sheet resistance versus strain for different glycerol contents. (g) Sheet resistance versus glycerol content (initial vs strained), showing that plasticizer‐assisted self‐alignment extends the negative response and stretchability to large strain [104]. Reproduced/adapted under the terms of the Creative Commons CC BY 4.0 International License. (h–j) Cyclic ΔR/R responses under triangular strain at different humidity and strain‐rate conditions, showing the transition from an in‐phase (resistance‐decreasing) to an out‐of‐phase response. (k) Phase‐shift angle Δθ versus relative humidity, decreasing as humidity rises. Together, the panels show that PEDOT:PSS nGF is governed by conductive‐core morphology, composition, plasticizer‐mediated chain mobility, and a humidity‐/rate‐dependent competition between PEDOT‐domain alignment (resistance decrease) and PSS‐matrix swelling (resistance increase). Reproduced/adapted with permission [105]. Copyright 2019 American Chemical Society.

Sezen‐Edmonds et al. [105]. reported a particularly revealing humidity‐controlled study of the alignment mechanism in PEDOT:PSS (Figure 14h–k). Under dry conditions the PEDOT‐rich domains remain mutually connected, and stretching biases the network toward enhanced alignment so that resistance decreases with strain. At relative humidities above approximately 50%, however, the PSS matrix swells with absorbed water and the connectivity between PEDOT domains is interrupted; under these conditions stretching merely separates the already‐isolated domains, and resistance increases with strain. At intermediate humidities the two competing tendencies — alignment and disconnection — coexist, and the response acquires a peculiar dependence on strain rate: at low strain rates (∼0.34% min^−^^1^) the resistance change is out of phase with the applied strain, while at high strain rates (∼1.12% min^−^^1^) it is in phase. The authors interpret this as a competition between two processes operating on different characteristic time scales. Postdeposition treatment with dichloroacetic acid removes excess PSS and renders the piezoresistive response invariant with humidity and strain rate. The humidity‐controlled study is important because it makes explicit the morphological condition that the alignment mechanism requires: the conducting domains must remain percolatively connected. When the matrix swelling separates them, alignment can no longer produce a network‐level resistance decrease.

A qualitatively different and arguably more remarkable mechanism — that of strain‐induced coalescence of conducting domains — was identified by Lee and coworkers study that has become the foundational reference for large‐strain nGF in PEDOT:PSS [106]. The defining experimental choice in that work was the use of a polyimide substrate, which matches PEDOT:PSS in Poisson's ratio (both approximately 0.34–0.35), in elastic strain limit (both approximately 2%), and approximately in elastic modulus (3.2 GPa for polyimide (PI) versus 2 GPa for PEDOT:PSS). The choice of substrate is critical: an earlier study by Lipomi and coworkers [107] on PEDOT:PSS deposited on PDMS substrates had established that the mismatched Poisson's ratios produce a sequence of mechanical failures — surface wrinkling at small strains, crack initiation around 12% strain, abrupt resistance increase around 30% strain as cracks accumulate — that obscure the intrinsic film response and result in a positive gauge factor dominated by the crack‐induced loss of percolation. On a PDMS substrate, by contrast, the large Poisson's‐ratio mismatch (0.5 for PDMS versus 0.35 for PEDOT:PSS) generates lateral compressive stress on the film during stretching, which produces dense buckles and subsequent cracking at strains above approximately 12%. Treatment of PDMS with O_2_ plasma or UV/O_3_ to improve PEDOT:PSS adhesion exacerbates the problem by generating thin SiO_x_ surface layers that fracture brittlely at even smaller strains. On PI, the matched mechanical properties of the substrate eliminate the local instability at the interface; the film and substrate deform uniformly, no cracks form, and the experimentally accessible strain window extends to 60% (limited only by rupture of the substrate itself). Within this window, pristine (undoped) PEDOT:PSS films exhibit a continuous, almost linear decrease in resistivity of approximately 80% from the initial value to that at 60% strain. Dimethyl sulfoxide (DMSO)‐doped films (5 or 10 wt%), by contrast, show invariant resistivity over the same strain range. The combination — pristine films show large nGF, doped films show none — uniquely separates the intrinsic morphological contribution from any geometric contribution and identifies a mechanism specific to the morphology of undoped PEDOT:PSS. This study thus established two critical points that have shaped subsequent work in this area: first, that substrate Poisson‐ratio matching is a prerequisite for observing intrinsic nGF in PEDOT:PSS at large strains; and second, that the absence of nGF in DMSO‐doped films, where the conducting morphology is already optimal in the unstrained state, identifies the morphological reorganization as the active mechanism.

The morphological evidence Lee and coworkers presented is striking (Figure 14a–c) [103]. AFM phase imaging of pristine films at strains of 0, 16%, 28%, and 42% reveals that the average surface area of PEDOT‐rich cores increases monotonically from 0.88 × 10^−^^2^ to 2.50 × 10^−^^2^ µm^2^, an enhancement of approximately 185%. By contrast, the surface area increase predicted from purely affine deformation of a single elliptical core under 42% uniaxial extension (with Poisson compression of 18% perpendicular to the strain direction) is only 16%. The order‐of‐magnitude discrepancy can be explained only by coalescence of multiple smaller cores into larger ones. Quantitative analysis of the AFM images, combined with the 3D variable range hopping framework, reproduces the experimental resistivity decrease to good accuracy: treating the core diameter as proportional to the VRH localization length ξ and using core diameters of 52, 64, 80, and 89 nm for strains of 0, 16%, 28%, and 42%, the predicted ρ(ε) curve coincides with the measured one. The mechanism, which Lee and coworkers term “mechanically induced annealing,” is that the elastic strain energy stored in the film provides the driving force for a thermodynamically favorable rearrangement of the morphology, in which small cores merge with larger ones through the connected channels (∼20 nm wide) that already exist in the as‐cast film. Energy minimization favors this coalescence because the PSS‐rich shells store more elastic energy per unit volume than the PEDOT‐rich cores (the cores being more compliant), and the merged morphology reduces the total shell volume per unit core volume.

The most consequential feature of the coalescence mechanism is that it is irreversible above approximately 17% strain. Lee and coworkers performed stretch–release cycles at maximum strains of 9, 17%, 26%, and 35%, finding that below 10% the resistivity change tracks reversibly along the same path during loading and unloading (with anisotropy between parallel and perpendicular directions consistent with elastic alignment of single cores), but above 17% the resistivity decrease is permanent: releasing the strain does not restore the initial resistance. Above 7% strain the resistivity change also becomes isotropic, with parallel and perpendicular slopes essentially identical, consistent with cores that have grown equally in all directions through coalescence rather than elongated by affine deformation. This irreversible isotropic enhancement of resistivity by mechanical strain is, as Lee and coworkers note, the first example of a mechanical analogue of chemical doping: a permanent change in the polymer's electrical properties induced by deformation alone.

This result was extended by the same group in a follow‐up study [103] that confirmed the coalescence mechanism using thicker films and a wider range of strains. The resistivity decrease in pristine PEDOT:PSS reaches 95% at strains above 60%, and the decreasing trajectory is independent of film thickness in the range studied. Lee and coworkers showed that the core growth proceeds by a “large‐eats‐small” mechanism in which larger cores absorb adjacent smaller ones, and that the local chemical environment within the cores changes upon coalescence (as evidenced by shifts in X‐ray photoelectron spectroscopy (XPS) binding energies), consistent with a denser packing of PEDOT segments and an attendant reduction in PSS character within the core. This morphological balance also controls the sign of the response: as shown in Figure 14d, PEDOT‐rich films (PEDOT:PSS ≈ 1:2.5) show a resistance that decreases by roughly an order of magnitude with strain as adjacent cores coalesce, whereas PSS‐rich films (≈ 1:6) show the opposite trend—resistance rising as the more widely separated cores are pulled further apart—directly demonstrating that the PEDOT‐rich, coalescence‐dominated morphology is what produces the negative gauge factor. The coalescence mechanism, in summary, provides a quantitatively validated VRH‐based account of large‐strain nGF in PEDOT:PSS that is morphologically and energetically distinct from the small‐strain alignment mechanism characterized by Vijay et al., and that operates in the strain regime (>17%) where alignment alone cannot explain the observations.

A useful counterpoint to the nGF studies is provided by the PEDOT:PSS systems engineered for strain insensitivity, in which the design objective is to balance the morphological and geometric contributions to the gauge factor so that the resistance remains nearly constant during stretching. Wang and coworkers [108] reported a highly stretchable, transparent, and conductive PEDOT:PSS formulation in which the addition of an ionic liquid and a fluorosurfactant produces films that retain transparency above 96% and conductivity above 3000 S cm^−^^1^ at strains of 100%, with only a modest increase in resistance under cyclic stretching. The mechanism, as characterized in that and subsequent work, involves a combination of plasticization and chain alignment that suppresses both the geometric resistance increase and the coalescence‐driven irreversibility. A more recent study by He and coworkers on salt‐induced ductilization of PEDOT:PSS with sodium perchlorate [109] achieved a similar outcome through a different chemical route: NaClO_4_ ions partially screen the Coulomb attraction between PEDOT^+^ and PSS^−^, increasing the elongation at break from 8.5% to 53.2% while leaving the tensile strength essentially unchanged. The treated films are nearly strain‐insensitive (resistance change of only ∼6% during cyclic tensile testing to 30% strain), and the authors explicitly attribute this to a balance between two competing contributions: a positive geometric contribution from longitudinal extension and lateral thinning, and a negative morphological contribution from strain‐induced orientation of PEDOT chains or aggregates. The strain‐insensitive systems, by demonstrating that the two contributions can be balanced against each other, indirectly confirm that the negative contribution identified in the nGF studies is real and is of a magnitude comparable to the geometric contribution. They also illustrate that the morphology‐based (VRH‐with‐strain‐dependent‐ξ) framework provides design rules in two opposite directions: by maximizing the morphological contribution (substrate matching, doping condition, plasticization), one can engineer large‐magnitude nGF for sensitive strain detection; by balancing it against the geometric contribution, one can engineer strain‐insensitive electrodes for stretchable interconnects in which the resistance must remain constant during deformation. The same underlying VRH‐with‐strain‐dependent‐ξ framework supports both design objectives.

### 4.3 nGF in PANI, PPy, and Textile‐Conducting Polymer Systems

The alignment and coalescence mechanisms identified in PEDOT:PSS are not unique to that polymer, and the broader single‐component conducting‐polymer literature contains parallel observations in polyaniline, polypyrrole, and conducting‐polymer‐coated textiles. These systems collectively introduce three additional mechanisms — crystallite bridging, surface‐state hopping, and yarn‐architecture‐mediated nGF — and they extend the empirical base of this category across polymer chemistries that span four decades of conducting‐polymer research, beginning with the classical drawing and doping studies of polyacetylene in the late 1980s, where mechanical orientation of conjugated polymer backbones was shown to produce highly anisotropic and nearly metallic charge transport.

Xiao and coworkers [20] demonstrated that polyaniline films undergo a large enhancement in conductivity upon cold stretching at room temperature (Figure 15f,g), with conductivity along the stretching direction increasing significantly as a function of applied strain. Structural characterization indicated that the cold‐drawing process forced both the polyaniline chains and the embedded crystallites to align with the draw direction, with attendant changes in morphology, molecular chain structure, crystallinity, and crosslinking of the films. While Xiao's experiments are post‐mortem rather than in situ — the conductivity is measured on the drawn film after release — the structural mechanism is the same as that operating in the in situ PEDOT:PSS measurements, providing an early demonstration that the alignment‐induced conductivity enhancement seen in modern in situ studies has a long‐standing structural precedent in the conducting polymer literature. The classical polyacetylene literature provides an even older precedent: stretching iodine‐doped (CH)x films produces a strong anisotropy σ∥/σ⊥ of order 10–100 and pushes the parallel conductivity into the metallic regime, again through alignment of the conjugated chains along the draw direction [98, 111]. The continuity of mechanism from Naarmann's 1987 work to Volkert's 2025 report is, by itself, a compelling argument that alignment‐driven nGF is not a peculiar artifact of any particular polymer system but a generic consequence of the morphological heterogeneity of conducting polymer films.

**FIGURE 15:**
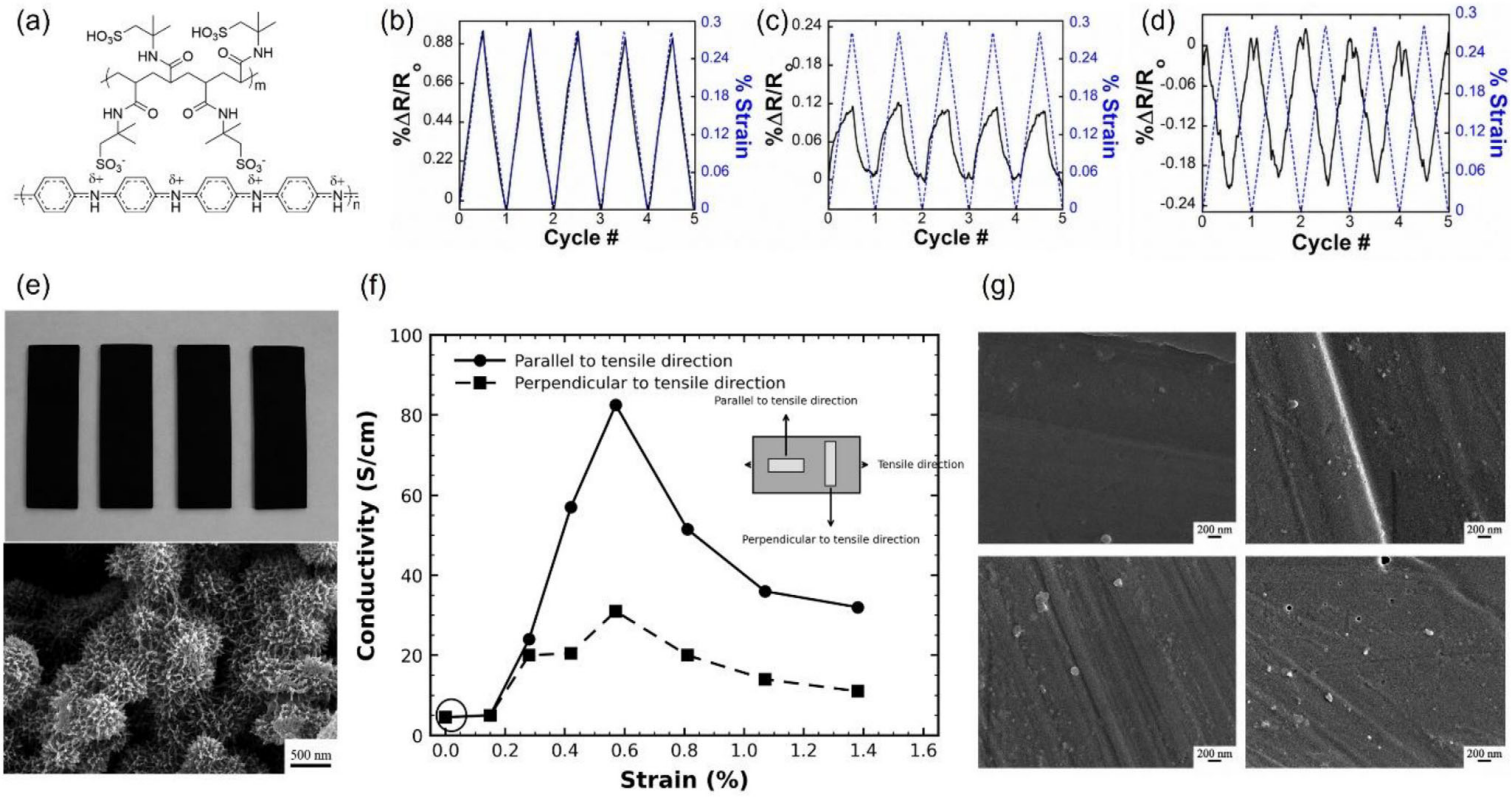
Polyaniline chain‐alignment and crystallite‐bridging mechanisms for polymer‐chain‐reorganization nGF. (a) Chemical structure of the electrically conducting PANI–PAAMPSA complex. (b–d) Cyclic piezoresistive responses (%ΔR/R_0_ under triangular strain) of PANI–PAAMPSA films with decreasing PAAMPSA molecular weight (PANI–PAAMPSA‐255, 106, 45), showing the gauge factor switch from positive (b) through weakly positive (c) to negative (d) as crystalline connectivity increases. Reproduced/adapted with permission [110]. Copyright 2017 American Chemical Society. (e) Photograph of free‐standing PANI emeraldine‐base films peeled from glass and the corresponding SEM of PANI powder (scale bar 500 nm). (f) Conductivity of cold‐stretched PANI films measured parallel and perpendicular to the tensile direction, peaking near the elastic‐limit strain (up to ∼18‐fold enhancement parallel to stretching). (g) SEM images of PANI films after cold stretching (scale bar 200 nm), evidencing strain‐induced chain/crystallite alignment as the structural origin of enhanced charge transport. Together, the panels show that polymer‐chain‐reorganization nGF in polyaniline arises when deformation increases crystalline connectivity and aligns conducting chains, lowering resistance along the strain direction. Reproduced/adapted with permission [20]. Copyright 2010 Wiley‐VCH GmbH.

A third mechanism, crystallite bridging, was identified by Sezen‐Edmonds et al. [110]. (Figure 15a–d) in a study of polyaniline–poly(2‐acrylamido‐2‐methyl‐1‐propanesulfonic acid) (PANI–PAAMPSA) thin films synthesized by template‐directed in situ polymerization. The defining observation in that work is that the sign of the gauge factor in PANI–PAAMPSA depends sensitively on the polymerization conditions, specifically on the aniline‐to‐PAAMPSA ratio that determines the crystalline content of the PANI phase. Films polymerized with low PANI crystallinity exhibit a conventional positive gauge factor of approximately +2, dominated by the geometric contribution. Films polymerized to produce higher PANI crystallinity, however, exhibit a negative gauge factor in cyclic strain testing. Structural characterization combining grazing‐incidence X‐ray diffraction with AFM identifies the operative mechanism as one in which PANI crystallites embedded in the amorphous PAAMPSA matrix serve as electrical bridges between previously disconnected conducting regions, with stretching aligning the crystallites preferentially and increasing the number of bridged pathways along the strain direction. The underlying physical picture is one in which the polymerization process determines whether the active PANI phase forms predominantly amorphous chains (which respond conventionally to strain) or organized crystalline aggregates (which can be rearranged into more effective bridging configurations). This crystallite‐bridging mechanism is mechanistically distinct from the alignment mechanism of Vijay and coworkers or the coalescence mechanism of Lee and coworkers: in the PANI–PAAMPSA case, the conducting domains themselves do not necessarily grow or align as a whole; rather, the connections between domains are reorganized through preferential alignment of the bridging crystallites. The same study examined PEDOT:PSS as a comparison material, finding that pristine PEDOT:PSS exhibits a positive gauge factor of +1.82 with a peculiar nonlinear plateau in the loading–unloading curve, and that dichloroacetic acid (DCA) treatment eliminates the plateau and reduces the gauge factor to +0.62, both still positive. The PANI–PAAMPSA result is significant because it demonstrates, perhaps more cleanly than any other study, that the sign of the gauge factor in a single‐component conducting polymer is a synthetic variable: by tuning the crystalline content of the active phase, the same polymer can be made to exhibit either +GF or −GF behavior. The broader implication is that the morphological details — domain size, crystalline content, matrix swelling state, substrate coupling — collectively determine the sign and magnitude of the response, and that the boundary between conventional positive piezoresistivity and nGF is a designable boundary rather than an intrinsic material property. This tunability is, in turn, the basis for the strategy adopted in much recent stretchable‐electronics work, in which the sign and magnitude of the response are selected by design choices rather than left to the intrinsic behavior of an off‐the‐shelf material.

A fourth mechanism, operating in single‐component conducting polymer thin films through surface‐state hopping enhancement under deformation, was characterized in detail by Barnoss et al., [112] in a study of chemically vapor‐deposited polypyrrole thin films exposed to inert gas (Ar, N_2_) and reactive gas (O_2_) atmospheres at controlled pressures. Although the experimental geometry is pressure‐on‐film rather than uniaxial stretch, the resulting piezoresistance response is the same physical phenomenon as the stretching‐induced response in the PEDOT:PSS systems discussed above — a strain‐driven change in the localization length of hopping states — and the Barnoss data therefore belong to this same category. The defining observation is a thickness‐dependent transition in the sign of the piezoresistance: films thinner than approximately 70 nm exhibit a pronounced negative piezoresistance, with the resistance dropping by approximately 5% upon application of 150 mbar of argon and saturating within seconds. Films thicker than approximately 70 nm exhibit a positive piezoresistance of comparable magnitude that develops on much slower timescales (minutes to hours) and does not saturate within reasonable experimental hold times. Films of intermediate thickness (∼70 nm) show a coexistence in which the polarity of the response can be switched by varying the gas pressure above approximately 500 mbar. The thickness scale at which the transition occurs coincides with the thickness scale at which the conductivity of these films saturates: the conductivity rises by nearly three orders of magnitude as the film thickness increases from 6 nm to 78 nm, then increases only weakly for thicker films. Barnoss and coworkers interpret this as evidence that the current‐carrying states in thin PPy films have two‐dimensional surface or interface character with a localization length that depends on the film thickness, and that the bulk conductivity emerges only when the film thickness exceeds approximately 70 nm. The negative piezoresistance is then attributed to mechanical compression of the surface layer, which increases the overlap between localized surface states and reduces the hopping resistance; the positive piezoresistance is attributed to gas‐molecule‐induced swelling of the bulk, which has the opposite effect. The interpretation is consistent with the temperature dependence of the conductivity of these films, which follows Efros–Shklovskii VRH at low temperatures and thermally activated hopping at higher temperatures [113]. The Barnoss thin‐film result is important for two reasons. First, it identifies a geometrically scaled polymer‐intrinsic mechanism — nGF appears only when the film is thinner than the dimensional crossover from surface‐dominated to bulk‐dominated transport — which is distinct from the bulk‐coalescence mechanism of Lee and coworkers and the bulk‐alignment mechanism of Vijay and coworkers. Second, it demonstrates that single‐component polypyrrole, in the appropriate geometric configuration, exhibits nGF without any added filler or composite architecture, which establishes polymer‐intrinsic nGF in a polymer system distinct from PEDOT:PSS and PANI.

A fifth group of observations belongs to the boundary between single‐component conductors and filler‐in‐matrix composites: nGF in conducting‐polymer‐coated textiles, where the polymer is a single‐component conductor but the strain transfer and the dominant resistance‐change pathway involve the woven or knitted yarn architecture rather than the polymer's own morphology. The most thoroughly characterized of these systems is the inkjet‐printed PEDOT:PSS on cotton fabric reported by Calvert and coworkers [49]. On cotton twill, cotton poplin, and combed‐cotton sateen, printed PEDOT:PSS exhibits a clear two‐stage strain response: an initial transient in which the surface PEDOT layer cracks (producing a brief resistance increase during the first few stretch cycles), followed by a stable, reproducible cyclic regime in which resistance decreases with applied strain and increases on release. The negative gauge factors reported for these systems are large: −5 for cotton poplin tested along the fabric axis, −9.50 for diagonal testing, −12 for cotton twill, −7 for combed‐cotton sateen, with the coarser twill and the diagonal‐direction tests giving the largest negative values. Calvert and coworkers interpret the mechanism as one in which the PEDOT:PSS that has penetrated into the yarn body provides the dominant conducting pathway after the cracking of the surface layer, and the lateral compression of the staple filaments within each yarn — which accompanies the untwisting of the yarn under tension — improves filament‐to‐filament contact and reduces the effective resistance. The same large negative gauge factors had been reported earlier by Wu et al., [114] for polypyrrole‐impregnated, Lycra and by Li and coworkers [115, 116] for polypyrrole‐coated nylon and cotton fabrics. Zhang and coworkers [117] extended the same architecture to knitted fabrics, where the deformation involves loop rearrangement in addition to yarn untwisting, and reported similar negative responses at very large strains. These textile‐based systems do not fit cleanly into either category as defined by the dominant mechanism: the conductor is a single‐component polymer, but the strain‐induced morphological change occurs at the fiber–fiber contact level, and the polymer's intrinsic chain reorganization plays at most a supporting role. The framework adopted here is to treat them as boundary cases in which polymer‐intrinsic and network mechanisms operate in parallel, with the textile architecture providing the dominant geometric mechanism and the polymer providing the conductive medium and contributing additionally through its own response at the fiber level. The pressure‐sensing analogue of these textile systems — polypyrrole‐coated polyurethane foam — has been demonstrated for compression rather than tension by Brady et al., [118] and reviewed in Dunne et al. [119]. Under compression, the PPy‐coated foam exhibits a positive (linear) conductance response with increasing pressure, that is, a decrease in resistance with increasing compressive strain, which is the compressive analogue of textile nGF and operates through the same fiber‐contact mechanism applied to a three‐dimensional porous architecture.

Across all five mechanisms — alignment, coalescence, crystallite bridging, surface‐state hopping, and textile architecture — strain modifies the conducting‐polymer morphology at the relevant length scale, changing the localization length or inter‐domain hopping resistance that governs charge transport in the VRH framework; because the geometric contribution is uniformly small (of order +2), even modest morphological responses produce net nGF. The active strain regime varies with the dominant mechanism, from reversible alignment at small strains (0%–10%) to irreversible coalescence at intermediate strains (10%–60%) and substrate‐ or plasticizer‐enabled operation at large strains. The sign can be tuned by environmental conditions [105](humidity), by synthetic conditions [110](PANI crystallinity), by film thickness [112](PPy <70 nm), and by substrate selection [106](PI versus PDMS) — design levers that distinguish morphological nGF from the more rigid sign behavior of intrinsic band‐structure mechanisms.

## 5 Architecture‐Induced nGF

### 5.1 Conceptual framework

The three preceding classes locate the origin of the negative response inside the conductor itself — in the band structure of a crystalline solid, the inter‐particle network of a filler dispersed in a matrix, or the morphological reorganization of a single‐component conducting polymer. The architecture‐induced class is defined instead by a displacement of that origin from the material to the geometry.

Here the negative gauge factor arises not because the conductor's intrinsic transport responds unusually to strain, but because a deliberately engineered architecture transforms the externally applied strain into a local strain of reversed sign. The conductor may be entirely conventional — a percolating composite, a near‐percolation metal film, or a conducting‐polymer coating — and would otherwise show the ordinary positive gauge factor of dimensional change or a crack‐induced positive response; it is the architecture that inverts this into nGF, independently of the conductor's chemistry.

The architecture‐induced class is best approached through its better‐known sibling: architecture used for strain accommodation rather than sign reversal. The foundational demonstrations of stretchable electronics — wavy single‐crystal silicon ribbons [120], controlled buckling of semiconductor nanoribbons [121], and biaxially stretchable wavy silicon nanomembranes [122] — all decouple a rigid material from the macroscopic strain field so that the substrate stretches while the active material deforms little, giving a gauge factor that approaches zero. Most architecture‐centric work (serpentine interconnects, kirigami, island‐bridge layouts) pursues this vanishing gauge factor. Architecture‐induced nGF instead occupies the regime in which the geometry re‐couples the strain with inverted sign — delivering macroscopic tension to the conductor as local compression, gap‐thinning, or contact closure. This distinction between strain‐accommodating (GF → 0) and nGF‐inducing (GF < 0) architecture organizes the section.

Two features connect the architecture‐induced class to the rest of this review. First, the geometric contribution remains, as in every class, uniformly positive and small (of order +(1 + 2ν) ≈ +2 for ν ≈ 0.5), so the architecture must supply a negative contribution exceeding this baseline for the net response to be negative. Second, these architectures couple the applied strain to a transport variable that depends exponentially on a geometric parameter — most clearly a tunneling gap, whose transparency depends exponentially on its width — so that a small architecture‐induced change produces a disproportionately large resistance change [16]. Because of this exponential coupling, the architecture‐induced class contains the largest nGF magnitudes documented in this review — conductivity increases of up to nine orders of magnitude and resistance‐change ratios approaching 10^8^ — far exceeding the Δρ/ρ_0_ of −15% to −95% characteristic of the polymer class, and often taking the form of a switch‐like insulator‐to‐conductor transition. Underlying all of this, the architecture does not itself generate charge transport but modulates the geometry within which a composite or polymer conductor carries current, so every architecture‐induced system is also a boundary case in which the geometry supplies the sign reversal and an underlying network or morphological mechanism supplies the resistance‐change physics.

### 5.2 Architecture‐Induced nGF Mechanisms

The most dramatic architecture‐induced nGF mechanism, and the one that most directly realizes the exponential coupling described above, is the strain‐thinned tunneling barrier (Figure 16b). Chae and coworkers [22] demonstrated a stretchable thin‐film device consisting of a sandwiched bilayer gold thin film separated by an ultrathin (≈12 nm) poly(dimethylsiloxane) elastomeric layer acting as a quantum‐mechanical tunneling barrier (Figure 17a–c).

**FIGURE 16:**
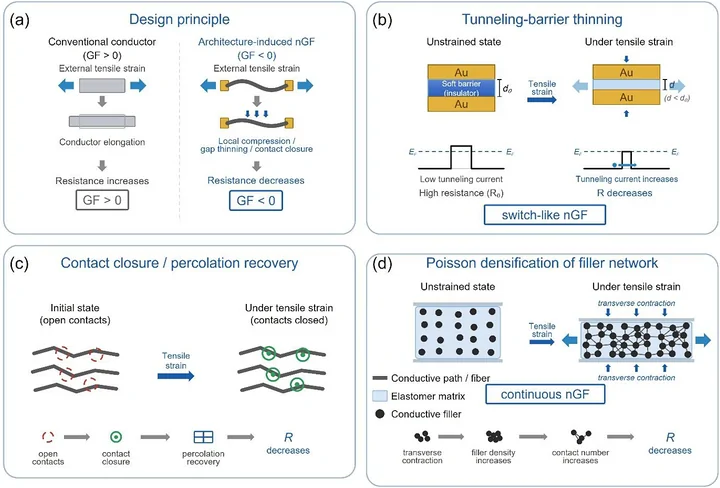
Architecture‐induced negative‐gauge‐factor mechanisms. (a) Design principle of architecture‐induced nGF: a conventional conductor under tensile strain elongates and usually shows GF > 0, whereas an engineered architecture converts macroscopic tension into local compression, gap thinning, or contact closure, producing GF < 0. (b) Tunneling‐barrier‐thinning mechanism in metal/soft‐barrier/metal structures, where tensile strain decreases the barrier thickness, increases the tunneling current, and lowers resistance in a switch‐like manner. (c) Contact‐closure / percolation‐recovery mechanism, where initially open conductive contacts close under tensile strain and restore percolating pathways. (d) Poisson‐densification mechanism in encapsulated filler networks, where transverse contraction increases filler density and contact number, producing a continuous negative resistance response. *Source*: Original schematic.

**FIGURE 17:**
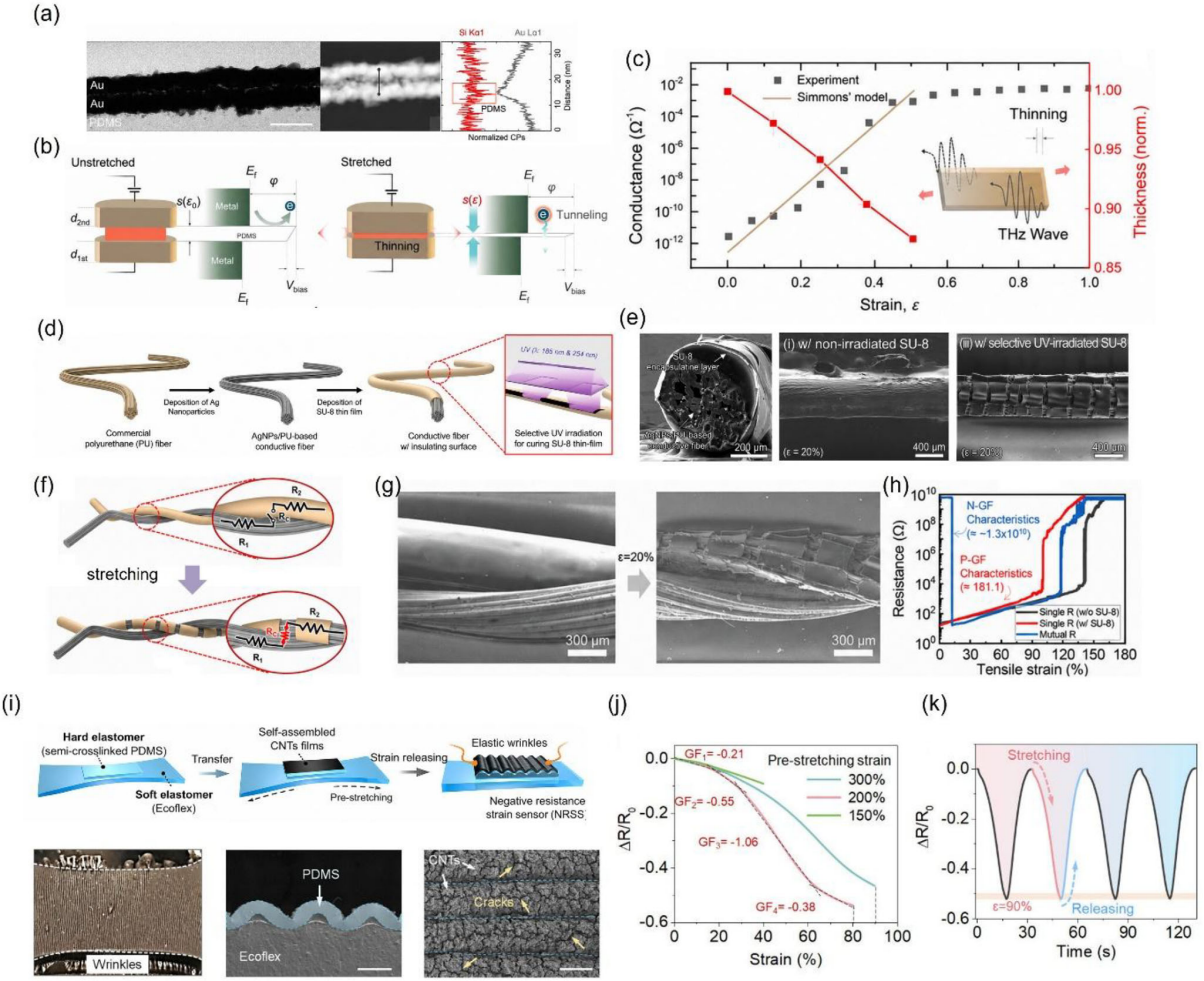
Representative architecture‐induced nGF systems based on tunneling, contact closure, and wrinkle engineering. (a) Cross‐sectional TEM/scanning transmission electron microscopy (STEM) and energy‐dispersive X‐ray spectroscopy (EDS) line profile of an Au/PDMS/Au junction, showing the soft PDMS tunneling barrier between two gold layers. (b) Energy‐level diagrams of the unstretched and stretched junction: tensile strain thins the barrier (s(ε_0_) → s(ε)), increasing electron tunneling. (c) Conductance versus strain with a Simmons'‐model fit, and the corresponding normalized barrier thickness measured by THz time‐domain spectroscopy, confirming strain‐induced barrier thinning. Reproduced/adapted with permission [22]. Copyright 2021 Wiley‐VCH GmbH. (d) Fabrication of an silver nanoparticle (AgNP)/PU conductive fiber coated with an SU‐8 thin film that is cured by selective UV irradiation (185 & 254 nm) to form an insulating, crack‐controlled surface. (e) SEM images of the SU‐8 encapsulation on the fiber and of non‐irradiated versus selective‐UV‐irradiated SU‐8 at ε = 20%. (f) Twisted‐fiber device and its equivalent‐circuit model (single‐fiber resistances R_1_, R_2_ and contact resistance R_C_). (g) SEM of a twisted fiber before and at ε = 20%. (h) Resistance versus tensile strain, showing the mutual resistance switching from a positive‐GF (≈ 181.1) to a negative‐GF (ΔR ≈ 1.3 × 10^10^) characteristic. Reproduced/adapted with permission [17]. Copyright 2023 Wiley‐VCH GmbH. (i) Soft–hard elastomer route (semi‐crosslinked PDMS on Ecoflex): transfer of self‐assembled carbon nanotube (CNT) films followed by strain release creates elastic wrinkles and nanocracks (negative‐resistance strain sensor, negative‐resistance switching (NRS)S); cross‐sectional and SEM images show the wrinkles, PDMS/Ecoflex interface, and CNT cracks. (j) ΔR/R_0_ versus strain for different pre‐stretching strains (150%–300%), with tunable negative gauge factors (GF ≈ −0.21 to −1.06). (k) Cyclic ΔR/R_0_ response under stretching/releasing at ε = 90%. Reproduced/adapted with permission [123]. Copyright 2025 American Chemical Society.

With the gold deposited near its percolation threshold, the unstrained device is electrically near‐insulating, because the intra‐film conduction path is incomplete and the soft barrier blocks inter‐layer transport. Upon stretching, the barrier is thinned at the quantum scale, the tunneling transparency rises exponentially, and the conductivity undergoes a transition from σ ≈ 10^−^^5^ S cm^−^^1^ at zero strain to σ ≈ 10^4^ S cm^−^^1^ at unity strain — a change of nine orders of magnitude, tunable over working strains as large as 130%. Because the deposition thickness of the metal and the thickness of the barrier can both be controlled, the same material system furnishes all three primitives of mechanical‐strain‐gated logic: an “on‐switch” (insulator‐to‐conductor transition upon stretching), an “off‐switch” (the conventional conductor‐to‐insulator transition), and a strain‐invariant interconnect, from which basic (AND, OR) and universal (NAND, NOR) logic gates were realized in a single stretchable thin‐film system. The tunneling‐barrier mechanism is the cleanest realization of the architecture‐induced sign‐reversal principle of Section 5.1: macroscopic tension is delivered to the barrier as a compressive thinning, and the exponential dependence of tunneling on barrier width converts this small geometric change into a switch‐type insulator‐to‐conductor transition.

A second mechanism achieves nGF not by thinning a tunneling gap but by closing a disconnected contact (Figure 16c) — engineering a crack or wrinkle architecture so that tensile strain brings conductive elements into contact rather than separating them, inverting the behavior of the ordinary crack‐based strain sensor in which widening cracks destroy percolation and produce a large positive gauge factor. Min and coworkers [17] realized this inversion in a twisted conductive fiber using a precision‐cracking technology (Figure 17d–h): selective ultraviolet irradiation produced a difference in the Young's modulus of the encapsulating layer along the fiber, engineered such that tensile strain regulates the mutual contact resistance between fiber elements downward rather than upward. The resulting negatively‐responsive switch‐type sensor exhibits an extremely large negative response — a resistance‐change ratio of approximately 3.9 × 10^8^, corresponding to a transition from insulating to conducting behavior — while retaining durability over more than 5000 stretching cycles, and its insulating quiescent state again yields near‐zero standby power, which the authors exploited in E‐fitness pants, smart masks, and posture‐correction T‐shirts. Beyond textiles, architecture‐engineered fused deposition modeling (FDM)‐printed conductive‐Tpolyurethane (PU) bend sensors have been demonstrated for hand‐rehabilitation gloves and finger‐joint monitoring [124, 125]. Li and coworkers [123] implemented the same contact‐closing principle in a planar geometry (Figure 17i–k) through an interfacial interlocked soft–hard elastomeric wrinkle: a hard CNTs/PDMS film was cross‐linked in situ on the surface of a pre‐stretched soft elastomer so that, on release, it buckled into micro‐wrinkles patterned with engineered nanocracks. Under tensile strain the wrinkle and crack faces are driven into closer contact, so that resistance decreases with elongation — the “anomalous” strain–resistance effect of the title — across the 0%–90% strain range and with stability over 5000 cycles. The superhydrophobic character of the interlocked surface additionally rendered the negative response stable underwater, extending the architecture to submerged electronics. In both the fiber and the planar cases the common principle is that the crack or contact geometry has been engineered to close under tension rather than to open, so that the percolation recovery characteristic of the compressive regime is delivered by a tensile load.

A third and milder mechanism is encapsulation‐mediated Poisson densification (Figure 16d), which applies the same compression‐under‐tension idea to a percolating filler network and yields a continuous, rather than switch‐type, nGF. The sandwich architecture itself has long been used for reasons unrelated to nGF — to protect a fragile conductive layer or to confer optical transparency, as in the transparent PDMS/carbon‐nanotube/PDMS strain sensors of Chen, Zhu, and Jiang [126], which operate as conventional positive‐gauge‐factor devices across a sensing range exceeding 250% strain. Dong and Xie [21] re‐engineered this sandwich specifically to exploit the compressive transmission, embedding a patterned carbon‐nanotube layer between two silicone‐rubber layers so that uniaxial extension of the encapsulant, accompanied by the Poisson contraction of its cross‐section, squeezes the nanotube layer and raises its packing density. The increased density multiplies inter‐tube contacts and drives the percolating network deeper into the connected regime, producing the negative resistance sensitivity coefficient the authors report, with a baseline resistivity tunable from approximately 12.7 to 403.2 Ω·m through the nanotube pattern. The conceptual significance is that an identical nanotube layer deposited without the compressive encapsulation would lose percolation under tension and exhibit a positive gauge factor; it is the sandwich geometry that reverses the sign. The Poisson‐densification mechanism is the continuous, modest‐magnitude analogue of the switch‐type tunneling and contact‐closing mechanisms: the same architecture‐supplied compression acts on a percolating filler rather than on a tunneling gap or an open crack, and the response is correspondingly graded rather than abrupt.

It is clarifying to contrast these nGF‐producing architectures with auxetic (negative‐Poisson's‐ratio) structures, which do not produce tensile nGF: an auxetic backing expands laterally under tension, separating conductive elements and driving the gauge factor positive, so such architectures serve to amplify a positive response or for compression sensing rather than sign reversal. This confirms the rule organizing the architecture‐induced class — tensile nGF requires a geometry that delivers compression, gap‐thinning, or contact closure under macroscopic tension, and is defeated by one that pulls the conductor apart. This class is the thinnest of the four in dedicated demonstrations, and its defining feature is that the negative contribution is a designable property of the structure rather than of the material: because the architecture cannot generate transport on its own, every architecture‐induced system also rides on a composite or polymer mechanism that the geometry drives in the favorable direction.

## 6 Application and Outlook

The four mechanistic classes developed in the preceding sections are not merely a taxonomy of physical origins; each class maps onto a distinct region of application space, and the boundaries between application domains track the boundaries between mechanisms more closely than is usually appreciated. The applications of nGF can be organized into four overlapping domains — high‐sensitivity strain sensing, strain‐insensitive electrodes and interconnects, switch‐type and logic devices, and ultra‐low‐power (Figure 18e) and environment‐specific monitoring — and each domain draws preferentially on the class whose characteristic gauge‐factor behavior matches its requirement. To place these application domains in the context of the underlying device physics, Table 2 compiles representative systems from all four mechanistic classes together with their material, dominant mechanism, loading mode, gauge‐factor definition (and its reporting basis), strain or pressure range, and original source, enabling a direct cross‐class comparison of the trade‐offs that shape the application domains discussed below.

**FIGURE 18:**
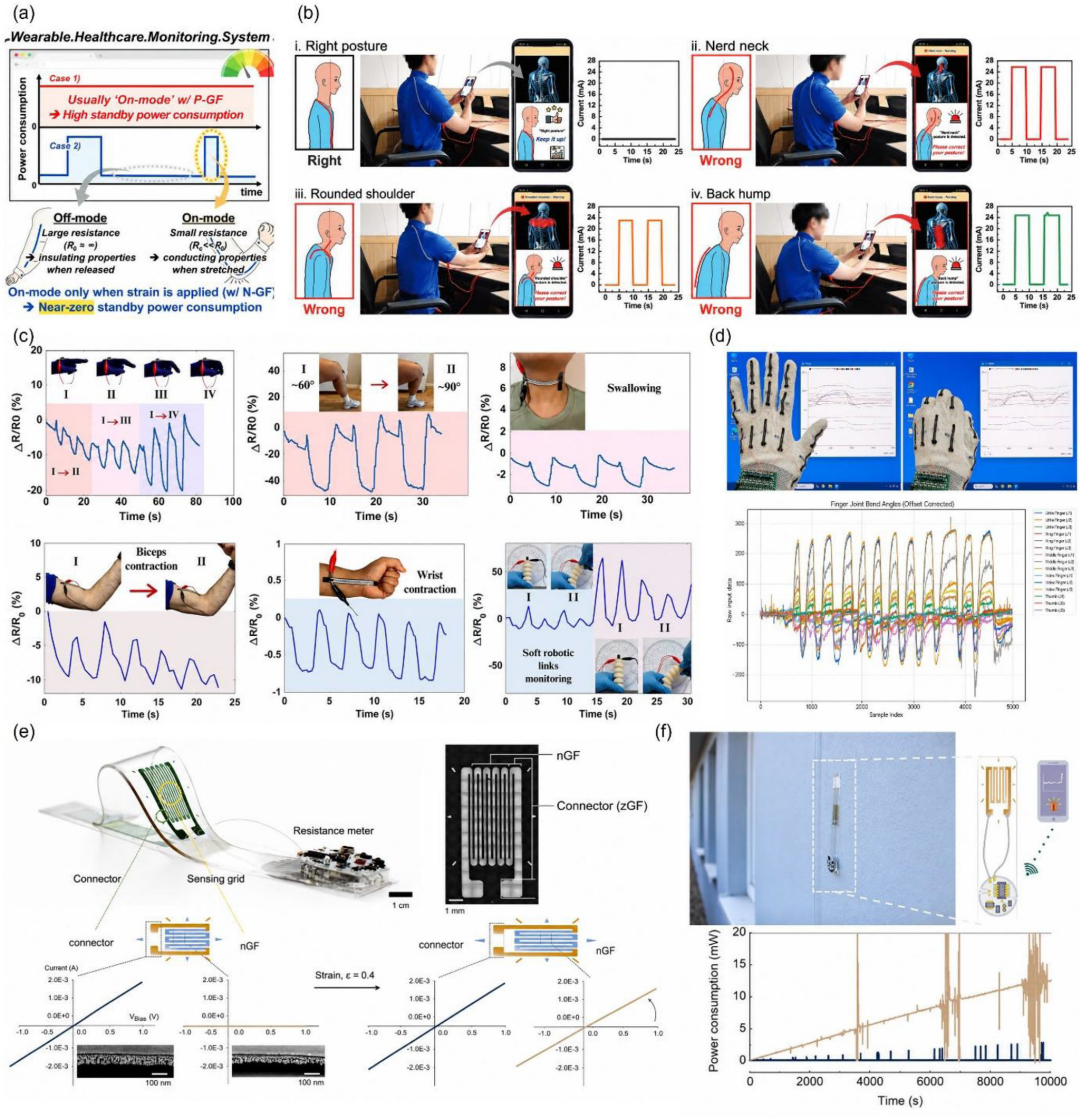
Emerging applications of negative‐gauge‐factor strain sensors in low‐power wearable, robotic, and distributed monitoring systems. (a) Operating principle for near‐zero standby power: a switch‐type negative‐resistance sensor stays in a high‐resistance “off” state (insulating, R_0_ ≈ ∞) when released and switches “on” (conducting, R_c ≪ R_0_) only when strain is applied — unlike a conventional positive‐GF sensor that is always on and continuously draws power. (b) Wearable posture‐monitoring demonstration (right posture, nerd neck, rounded shoulder, back hump) with the corresponding current responses and a smartphone‐app alert. Reproduced/adapted with permission [17]. Copyright 2023 Wiley‐VCH GmbH. (c) Body‐motion and physiological monitoring via ΔR/R_0_ traces: finger bending, knee flexion (∼60° → ∼90°), swallowing, biceps and wrist contraction, and soft‐robotic‐link sensing. Reproduced/adapted with permission [127]. Copyright 2025 Elsevier. (d) Smart data glove for finger‐joint tracking, with multi‐joint bend‐angle signals from an FDM‐printed conductive thermoplastic polyurethane (TPU) bend sensor [125]. Reproduced/adapted under the terms of the Creative Commons CC BY 4.0 International License. (e) Flexible sensing grid combining nGF sensing regions with near‐zero‐gauge‐factor (zGF) connectors; the connector stays ohmic while the nGF element switches under strain (I–V curves at ε = 0 and 0.4), with the cross‐sectional microstructure shown. (f) Wall‐mounted distributed structural/environmental monitoring and the corresponding power consumption over time, showing the much lower standby power of the nGF system versus a conventional sensor. Reproduced/adapted with permission [16]. Copyright 2025 American Chemical Society.

**TABLE 2:** Comparison of representative negative‐gauge‐factor (nGF) systems across the four mechanistic classes. Values are quoted with their reporting definition where determinable; “n.r.” = not reported. π = longitudinal piezoresistance coefficient.

Category	Material / system	Mechanism	Loading mode	Gauge factor (definition)	Strain / pressure range	Reference / source
**Intrinsic**	Bulk n‐Si	Many‐valley valley repopulation	Tensile	≈ −135 (peak; orientation‐dep.)	Elastic (<∼1%)	Smith, 1954 [30]
**Intrinsic**	Si nanowire, ⟨111⟩ p‐type	Giant PZR, surface‐state‐mediated	Tensile	π_l_ up to −3550 × 10^−^^1^ ^1^ Pa^−^^1^ (diam.‐dep.)	Low‐strain linear	He & Yang, 2006 [18]
**Intrinsic**	Si nanowire (ultra‐high strain)	pGF→nGF valley redistribution	Tensile	≈ −26 (estimated secant GF)	Nonlinear (sign reversal)	Lugstein et al., 2010 [55]
**Intrinsic**	3C‐SiC nanowire (B‐doped)	Schottky / band restructuring	Compressive (C‐AFM)	π ≈ −103 × 10^−^^1^ ^1^ Pa^−^^1^; GF up to −620.5	Strongly nonlinear	Li et al., 2016 [54]
**Intrinsic**	SiC nanowire (N,P co‐doped)	Co‐doping donors + Schottky	Compressive (C‐AFM)	π ‐5.07→−146.3 × 10^−^^1^ ^1^ Pa^−^^1^; GF up to −877.8	AFM tip force: 24.95–130.51 nN	Cheng et al., 2019 [53]
**Intrinsic**	MoS_2_ (mono/bi/tri, membrane)	Strain‐induced bandgap narrowing	Biaxial (membrane)	−148 / −224 / −43.5 (differential)	n.r.	Manzeli et al., 2015 [66]
**Intrinsic**	MoS_2_ FET (gate‐tunable)	Bandgap + Schottky, gate‐tuned	Tensile	up to −40 at V_g = 20 V (gate‐tunable)	n.r.	Tsai et al., 2015 [76]
**Intrinsic**	PtSe_2_ film	Bandgap modulation	Tensile (cantilever)	up to −85	n.r.	Wagner et al., 2018 [19]
**Intrinsic**	PdSe_2_ film (transfer‐free)	Bandgap modulation	Tensile & compressive	up to −315	n.r.	Zhang et al., 2024 [67]
**Composite**	MoS_2_/PEO composite	Strain transfer to nanosheets	Tensile	−25 (MoS_2_); −12 (WS_2_); −2 (MoSe_2_/WSe_2_)	Non‐monotonic	Biccai et al., 2019 [25]
**Composite**	Solution‐processed PtSe_2_ network	Strain transfer (η≈8.5%)	Tensile	≈ −5.45	n.r.	Ilhan et al., 2025 [24]
**Composite**	Graphene‐coated textile	Yarn reorganization / contact	Tensile (anisotropic)	−1.7 (x); −26 (y)	Direction‐dependent	Yang et al., 2018 [88]
**Composite**	rGO smart fabric	Structure‐based contact enhancement	Tensile (+twist/shear)	≈ −5.92 at 10.7%	n.r.	Hong et al., 2021 [87]
**Polymer**	PEDOT:PSS (alignment)	Domain/chain alignment (VRH ξ↑)	Tensile	Δρ/ρ_0_ ≈ −15% at ∼14% (secant)	Non‐monotonic	Vijay et al., 2011 [102]
**Polymer**	PEDOT:PSS/PVA:glycerol	Plasticizer‐facilitated alignment	Tensile	R_s → ∼20% of R_0_ (min 65–144%)	Three‐regime	Volkert et al., 2025 [104]
**Polymer**	PEDOT:PSS (coalescence)	Irreversible core coalescence	Tensile	Δρ/ρ_0_ ≈ −90% to 60% (secant)	Quasi‐linear	Lee et al., 2016 [103]
**Polymer**	PANI–PAAMPSA	Crystallite bridging (polarity‐tunable)	Tensile	Sign‐tunable (+→−) via MW	n.r.	Sezen‐Edmonds et al., 2017 [110]
**Polymer**	Polypyrrole thin film (<70 nm)	Surface‐state hopping	Compressive (gas pressure)	ΔR/R ≈ −5% at 150 mbar	n.r.	Barnoss et al., 2009 [112]
**Architecture**	Au/PDMS/Au tunneling barrier	Barrier thinning (SWITCH)	Tensile	σ 10^−^^5^→10^4^ S cm^−^^1^ (9 orders)	Switch‐like	Chae et al., 2021, 2025 [16, 22]
**Architecture**	Twisted conductive fiber (SU‐8)	Contact closing (SWITCH)	Tensile	ΔR ratio ≈ 3.9 × 10^8^	Switch‐like	Min et al., 2023 [17]
**Architecture**	Soft–hard CNT wrinkle (NRSS)	Contact closing (wrinkle/crack)	Tensile (+underwater)	−0.21 to −1.06 (secant)	Tunable	Li et al., 2025 [123]
**Architecture**	CNT / silicone sandwich	Poisson densification	Tensile	Negative resistance‐sensitivity	Continuous	Dong & Xie, 2021 [21]

Gauge‐factor definitions: differential = local slope d(ΔR/R_0_)/dε; secant = ΔR/R_0_ over the stated interval; peak = maximum reported value. Full citations and DOIs are given in the reference list.

The first and most direct application domain is high‐sensitivity strain sensing with sign selection. The defining advantage of an nGF sensor over a conventional positive‐gauge‐factor device is not magnitude alone but the access it gives to the opposite sign of response, which expands the available design space for human‐motion capture, soft robotics, and electronic skin (Figure 18c,d) [125, 127]. The polymer‐intrinsic morphological mechanisms supply continuous, graded negative responses suitable for proportional sensing: the strain‐induced coalescence of PEDOT‐rich cores produces resistivity decreases of up to 95% over large strains [103, 106], and the synthetic tunability of the PANI–PAAMPSA system [110] demonstrates that the sign of the response can be selected at the materials‐synthesis stage rather than imposed by device geometry. Where a graded response is not required and a threshold‐like response is preferable — for instance, in event detection — the architecture‐induced switch‐type mechanisms provide resistance‐change ratios reaching 3.9 × 10^8^ [17] and conductivity transitions of nine orders of magnitude [16, 22], far exceeding any continuous mechanism.

The second domain is the inverse design objective of strain‐insensitive electrodes and interconnects, in which the goal is a vanishing rather than a large gauge factor, achieved by balancing the uniformly positive geometric contribution of order +(1 + 2ν) against a negative non‐geometric contribution of comparable magnitude. This balancing is the explicit design principle behind the strain‐tolerant PEDOT:PSS formulations of Wang and coworkers [108] and the salt‐ductilized films of He and coworkers [109], in which a polymer‐intrinsic morphological negative contribution is tuned to cancel the geometric increase, and behind the PEDOT:PSS/acrylamide organogel conductors of the Joo group [99]. The same cancellation is achieved architecturally in the compliant‐mechanism conductor of Lu and coworkers, whose resistance change ratio remains within 0.05% over the entire 0%–200% strain range [128]. That the identical balance can be reached either morphologically (polymer‐intrinsic) or architecturally (architecture‐induced) is a direct illustration of the cross‐category design continuum emphasized throughout this review.

The third domain, and the one most exclusively dependent on a single class, is mechanical switching and logic. Because the architecture‐induced tunneling‐barrier architecture can be configured as an on‐switch, an off‐switch, or a strain‐invariant interconnect within a single material system, it furnishes all the primitives required for material‐embedded logic without conventional transistors, and Chae and coworkers [22] used this to realize basic (AND, OR) and universal (NAND, NOR) mechanical‐strain‐gated (Figure 19a,b) logic gates. No mechanism in the other three categories can produce the abrupt, high‐contrast, insulator‐to‐conductor transition that logic operation requires; this application is the unique province of architecture‐induced switch‐type nGF. A closely related extension of this strain‐gated switching concept is the use of negative‐resistance switches to program pixel activation in stretchable displays (Figure 19c,d). Min and co‐workers demonstrated a fabric‐based passive‐matrix LED array in which hidden pixels remain electrically disconnected in the relaxed state but are selectively activated when tensile strain is applied [17]. In this design, normal pixels are directly connected to the power supply through conductive fibers and therefore remain visible regardless of deformation, whereas hidden pixels are routed through x‐axis and/or y‐axis NRS fiber strain sensors. As the textile is stretched, the relevant NRS fibers undergo an insulator‐to‐conductor transition, completing the circuit only for the pixels assigned to that deformation direction. Consequently, x‐axis stretching, y‐axis stretching, and biaxial stretching activate different hidden‐pixel groups, compensating for the increased inter‐pixel spacing and helping maintain apparent pixel density and luminance under deformation. This example shows that architecture‐induced nGF can function not only as a binary mechanical switch or logic element, but also as a strain‐addressable circuit component for adaptive stretchable displays.

**FIGURE 19:**
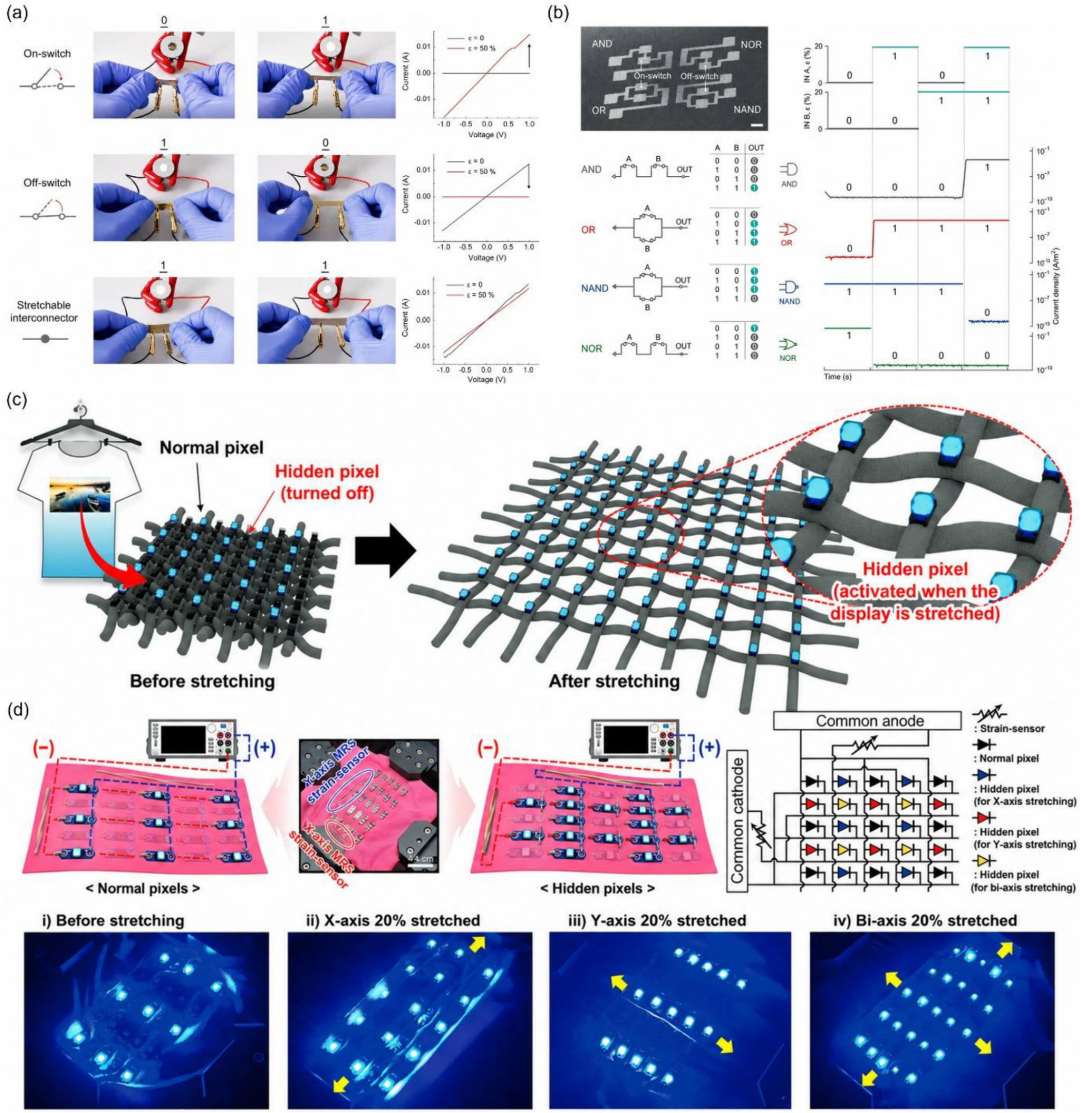
Mechanical‐switching and hidden‐pixel display applications enabled by negative‐resistance strain sensors. (a) Soft tunneling‐barrier devices operated as an on‐switch, an off‐switch, and a stretchable interconnector, with light‐emitting diode (LED) demonstrations and the corresponding current–voltage characteristics at ε = 0 and 50%. (b) Stretchable mechanical logic gates (AND, OR, NAND, NOR) built from strain‐gated on/off switches, with device images, circuit diagrams, truth tables, and time‐resolved current‐density outputs for the input combinations of A and B. Reproduced/adapted with permission [22]. Copyright 2021 Wiley‐VCH GmbH. (c) Concept of a stretchable woven display whose hidden pixels remain off before stretching and are selectively activated when the display is deformed. (d) Biaxially stretchable 5 × 5 LED array integrated with x‐ and y‐axis negative‐resistance‐switching strain sensors, showing the hidden‐pixel equivalent circuit and the display under no strain and under x‐axis (20%), y‐axis (20%), and biaxial (20%) stretching. Reproduced/adapted with permission [17]. Copyright 2023 Wiley‐VCH GmbH.

As emphasized in the Introduction, this low‐power advantage is a property of the switch‐type architecture rather than of a negative gauge factor in general: the continuous nGF mechanisms of the intrinsic, composite, and polymer classes still draw power continuously and are valued instead for graded, high‐sensitivity response. The fourth domain is ultra‐low‐power (Figure 18e) and environment‐specific monitoring, which again exploits the insulating quiescent state of the architecture‐induced switch‐type devices. Because such a device draws current only after a strain threshold closes its conduction path, its standby power consumption is approximately 10^−^^5^ mW — more than six orders of magnitude below a conventional always‐on resistive sensor — which makes it suited to long‐term, energy‐limited wearable and structural monitoring (Figure 18a,b,f) [16, 17], and the latter authors demonstrated this in E‐fitness pants, smart masks, and posture‐correction garments. Where the operating environment is itself the constraint, the superhydrophobic interlocked‐wrinkle architecture of Li and coworkers [123] extends stable negative‐resistance operation underwater, and the compressive‐mode foam systems discussed in Section 4.3 [118, 119] extend it to three‐dimensional body‐contour monitoring. Across all four domains, the recurring lesson is that the application requirement — graded versus threshold response, large versus vanishing gauge factor, continuous versus switch‐type behavior, power budget, operating environment — selects the mechanism, and that this framework therefore functions as a design map rather than only as a classification.

## 7 Perspective and Conclusion

Three problems stand out as the principal obstacles to the maturation of nGF as a design resource. The first is reversibility and hysteresis. Several of the most powerful mechanisms are intrinsically irreversible — the strain‐induced coalescence of PEDOT‐rich cores becomes permanent above approximately 17% strain [103], constituting a mechanical analogue of chemical doping rather than a recoverable sensor response — and even the switch‐type architecture‐induced devices, although demonstrated over thousands of cycles, require careful crack and barrier engineering to suppress drift. A predictive understanding of when a morphological or architectural change is recoverable, and how to engineer recoverability without sacrificing magnitude, remains incomplete. The second is the absence of a single quantitative model spanning the categories. The variable‐range‐hopping framework with a strain‐dependent localization length accounts for single‐component conducting polymers, the exponential dependence of tunneling on barrier width accounts for the architecture‐induced tunneling mechanism, and percolation theory accounts for composite networks; but these are presently separate accounts, and a unified treatment that places the positive geometric term and the category‐specific negative term on a common footing would substantially strengthen the field's predictive power. The third is the lack of reporting standardization: nGF results are reported with inconsistent sign conventions, strain ranges, and decompositions of the geometric versus non‐geometric contributions, which complicates cross‐study comparison and obscures the boundary cases that are often the most instructive.

To enable fair comparison, we recommend that future nGF reports specify: (1) the gauge‐factor definition (differential, secant, average, or peak); (2) the strain or pressure interval over which it was extracted; (3) the loading direction; (4) the current (measurement) direction; (5) the strain rate; (6) the substrate mechanical properties; (7) the initial resistance R_0_; (8) the environmental conditions (humidity, temperature); (9) hysteresis; (10) recovery/reversibility; (11) drift; (12) cyclic durability; and (13) the measured electrical power consumption, including standby power. Adopting at minimum items (1), (2), and (13) would substantially improve the comparability and reproducibility of nGF sensor reports.

The most promising forward directions follow from the cross‐category continuum. Hybrid designs that deliberately couple an architecture‐induced design to an intrinsic, composite, or polymer conductor — for example, an architecture engineered to deliver compression to an intrinsically negative‐piezoresistive 2D filler — could in principle combine the large magnitude of the architectural sign reversal with the graded controllability of the material response. Mechanical computing, foreshadowed by the strain‐gated logic gates of Chae and coworkers, points toward material‐embedded logic of greater complexity. Energy‐autonomous and event‐driven sensing, enabled by the insulating quiescent state of switch‐type nGF, is well matched to the power constraints of long‐term wearable and implantable monitoring. Operation in demanding environments, demonstrated underwater by the interlocked‐wrinkle architecture, suggests extension to other harsh settings. Finally, the high dimensionality of the design space — filler chemistry, matrix compliance, polymer crystallinity, substrate matching, barrier thickness, crack and wrinkle geometry — makes nGF an attractive target for data‐driven and inverse‐design approaches that search the property space for a specified gauge‐factor sign, magnitude, strain range, and reversibility, rather than discovering these behaviors one system at a time.

In closing, the negative gauge factor, long regarded as an anomaly or a curiosity, emerges from the survey presented here as a coherent and designable family of phenomena. The framework developed here — intrinsic band‐structure response, composite network reorganization, single‐component polymer morphological reorganization, and architecture‐induced strain‐field transformation — organizes a literature spanning four decades and several materials systems around a single accounting principle: a uniformly positive geometric contribution set against a negative contribution whose physical seat, coupling variable, and magnitude define the category. The boundaries between the categories are real but porous, and the most capable devices exploit that porosity. With the central problems of reversibility, unified modeling, and reporting standardization addressed, the negative gauge factor is positioned to become not an exception to be explained but a design degree of freedom to be exploited across stretchable electrodes, conformal and proportional sensors, mechanical logic, ultra‐low‐power monitoring, and environments where conventional positive‐gauge‐factor devices cannot operate.

## Author Contributions

**Su‐Hyeong Lee**: methodology, investigation, writing – original draft. **Minjae Kim**: methodology, investigation, writing – original draft. **Soosang Chae**: methodology, validation, supervision, resources, funding acquisition, writing – review and editing, writing – original draft.

## Funding

Ministry of Education (MOE) and Chungcheongnam‐do, Republic of Korea (ANCHOR program, 2026‐ANCHOR‐12‐022); Education and Research Promotion Program of KOREATECH (2024, 2025). Korea government (MSIT) (No. RS‐2026‐25549986).

## Use of Generative AI and AI‐Assisted Technologies in the Writing Process

The authors used ChatGPT, a large language model developed by OpenAI, to assist with language editing, structural organization, and refinement of selected figure captions during manuscript preparation. An artificial‐intelligence (AI)‐based image‐generation tool was also used to prepare a draft Table‐of‐Contents graphic. All scientific content, interpretations, references, figure selections, and final manuscript text were critically reviewed, verified, and approved by the authors. The AI tools were not used as authors and did not make independent scientific conclusions.

## Conflicts of Interest

The authors declare no conflicts of interest.

## Data Availability

Data are available from the corresponding author upon reasonable request.
